# High expression of new genes in trochophore enlightening the ontogeny and evolution of trochozoans

**DOI:** 10.1038/srep34664

**Published:** 2016-10-04

**Authors:** Fei Xu, Tomislav Domazet-Lošo, Dingding Fan, Thomas L. Dunwell, Li Li, Xiaodong Fang, Guofan Zhang

**Affiliations:** 1 Key Laboratory of Experimental Marine Biology, Institute of Oceanology, Chinese Academy of Sciences, Qingdao 266071, China; 2 Laboratory for Marine Biology and Biotechnology, Qingdao National Laboratory for Marine Science and Technology, Qingdao 266071, China; 3 National & Local Joint Engineering Laboratory of Ecological Mariculture, Institute of Oceanology, Chinese Academy of Sciences, Qingdao 266071, China; 4 Laboratory of Evolutionary Genetics, Ruđer Bošković Institute, Bijenička cesta 54, P.P. 180, HR-10002, Zagreb, Croatia; 5 Catholic university of Croatia, Ilica 242, HR-10000, Zagreb, Croatia; 6 BGI-Shenzhen, Shenzhen, 518083, China; 7 Department of Zoology, University of Oxford, South Parks Road, Oxford, OX1 3PS, UK

## Abstract

Animals with trochophore larvae belong to Trochozoa, one of the main branches of Bilateria. In addition to exhibiting spiral cleavage and early cell fate determination, trochozoans typically undergo indirect development, which contributes to the most unique characteristics of their ontogeny. The indirect development of trochozoans has provoked discussion regarding the origin and evolution of marine larvae and is interesting from the perspective of phylogeny-ontogeny correspondence. While these phylo-onto correlations have an hourglass shape in Deuterostomia, Ecdysozoa, plants and even fungi, they have seldom been studied in Trochozoa, and even Lophotrochozoa. Here, we compared the ontogenetic transcriptomes of the Pacific oyster, *Crassostrea gigas* (Bivalvia, Mollusca), the Pacific abalone, *Haliotis discus hannai* (Gastropoda, Mollusca), and the sand worm *Perinereis aibuhitensis* (Polychaeta, Annelida) using several complementary phylotranscriptomic methods to examine their evolutionary trajectories. The results revealed the late trochophore stage as the phylotypic phase. However, this basic pattern is accompanied with increased use of new genes in the trochophore stages which marks specific adaptations of the larval body plans.

The reconstruction of the Metazoan ancestor has been challenging due to the existence of indirect developmental life cycles, which are common in the main branches of bilateral animals. Some marine phyla consist of animals that undergo biphasic development characterized by a free-swimming larval stage during ontogenesis^1^. For instance, annelids and mollusks are characterized by ciliated trochophore larvae (Fig. 1), and are both placed within the Trochozoa^2^, a group of spiralian animals which account for at least one-third of all marine species^3^. Trochozoans are often included in studies that discuss the origin of the metazoan body plan^4^,^5^. The increasing number of sequenced trochozoan genomes has facilitated the investigation of their developmental patterns^3^,^6^. For example, Hox genes are present in all of these animals^3^ and in some species start to be expressed during mid-embryogenesis, consistent with observations made in other phyla^7^. These findings further suggest the possible existence of common regulatory mechanisms that are ancestral to all animals. Indeed, the phylotypic stage, a phylum specific ontogenetic period, has been assigned to a broad range of phyla^8^, and the common phylogeny-ontogeny correlation has an “hourglass” or “egg timer” shape^9^. In vertebrates, the timing of Hox gene expressions was considered to be an important molecular indicator of phylotype^9^, this has also been well studied on the basis of transcriptomic data that consistently indicate the presence of phylotypic stages^9^,^10^. Taking gene regulatory networks into consideration, the hourglass model of embryogenesis can be explained by a hypotheses in which the early stages of embryogenesis are highly diversified as a result of variations in egg construction, which are driven by adult adaptation to the environment. This results in low similarity between species at this early developmental stage. As the embryo develops, phylogenetically conserved regulatory mechanisms become fully operative and assert increasing developmental control, resulting in phylotypic stages with high similarity. Finally, the fully developed diversified gene networks begin assert their influence resulting in adult bodies with low similarity^11^.

While the hourglass profiles in ontogenic transcriptomes has been well described in Ecdysozoa (*Drosophila*, *Caenorhabitis*)^10^,^12^, plants (*Arabidopsis*)^13^, and fungi (*Coprinopsis*)^14^ it has however been poorly explored whether the convergence of mid-ontogeny is also present in trochozoans^8^,^15^,^16^. Moreover, it is unclear whether the trochophores of different phyla are subject to similar molecular regulatory mechanisms. To help answer these questions, we analyzed the transcriptome characteristics of the main developmental stages in three trochozoans: the Pacific oyster, *Crassostrea gigas* (Bivalvia, Mollusca), the Pacific abalone, *Haliotis discus hannai* (Gastropoda, Mollusca), and the sand worm *Perinereis aibuhitensis* (Polychaeta, Annelida). We also conducted genome re-sequencing in ten individual *C. gigas* oysters to analyze the relationships between genetic variation and the expression patterns observed during embryogenesis.

## Results

### The transcriptome age index of three trochozoans

The transcriptome age index (TAI) was calculated for different development stages of the three trochozoans. When all genes are considered, the results revealed that the trochophore larvae show the youngest transcriptome of all ontogentic stages (Fig. 2). However, this pattern was mainly affected by species-specific genes, *i.e.* the youngest phylostratum (ps11 oyster, ps11 abalone and ps10 sand worm, Fig. 2 and Supplementary Fig. S1). We found that only about 56 genes (51 from ps11 and 5 from ps10) contribute to increased TAI in trochophores in oyster, and oyster trochophores show the lowest TAI when these genes were excluded from the analysis (Supplementary Fig. S2, S3 and Supplementary Table S1). The overall pattern was robust when we calculated TAI for the oyster dataset with all the 38 developmental samples and all the annotated genes included (see Supplementary Fig. S4). Thus, the selected development stages used in comparative analyses of the three trochozoans reflect well the basic transcriptome pattern.

To get a more detailed picture of TAI differences we have depicted the relative expression levels (REL) for each phylostratum across the oyster ontogeny. It was evident that two groups of phylostrata exhibited opposing trends in expression after the trochophore stage (Fig. 3). The first group of phylostrata (ps1, 3, 6 and 8) show a rapid increase in relative expression values immediately following the trochophore stage. By contrast, the relative expression values of the other group (ps4, 5, 10 and 11) show a striking decrease. These differences reach their maximum at the late trochophore stage. Genes in phylum phylostratum (ps9) also has a quick increased REL during trochophore. In contrast, expression of ps2 genes shows decreasing pattern along the development, while ps7 has the lowest REL at the trochophore stage.

### The transcriptome similarities between trochozoans

As the TAI catches only one aspect of evo-devo correlations it is good to complement it with other phylo-transcriptomic measures^17^,^18^. Therefore, we further analyzed the similarities between the transcriptomes of the three species over the course of embryogenesis. According to one view the adaptive pressures for changes in the phylotypic stage of development are minimized^15^. For this reason, this stage is most likely to retain features found in the last common ancestor; thus, similarities in the transcriptomes of the three trochozoan species should be highest during the phylotypic stage^9^. We identified orthologous genes between the species pairs and compared the transcriptomes of the different embryonic stages in an all-to-all manner. This analysis shows a pretty good correspondence between developmental stages of the three species (Fig. 4). We generally found the highest similarity during the trochophore period between the two mollusks. The high similarities were also seen between the metatrochophore of sand worm and veliger of abalone. For sand worm and oyster, we detected the highest similarity in the gastrula stage that precedes the trochophore period (Fig. 4).

### The transcriptome polymorphism index of oyster

So far we tested profiles at the macroevolutionary scale. To further explore the conservation level within the population we also developed the novel transcriptome polymorphism index (TPI). This measure estimates, akin of TAI and TDI^13^, the mean sequence diversity of a transcriptome by weighting the nucleotide diversity (*θ*) of each gene by its expression level. By obtaining genome re-sequencing data for 10 oyster individuals we found that the TPI was lowest during the trochophore stage (Fig. 5a), which indicated that the transcriptome at this stage is more conserved.

### The expression pattern of oyster Hox family genes

The conservation of genes expressed during trochophore stage can also be seen in Hox gene expression pattern. As molecular characteristics of vertebrate phylotype, the timing of Hox gene expression coincides with the pharyngula stage^9^,^19^,^20^. Here we surveyed the expression pattern of the ten Hox genes in oyster (Fig. 5b). The expression timing for oyster Hox genes follows a similar temporal pattern between each group of Hox genes; the Hox1-Hox2-Hox3 and Hox4-Hox5 anterior group, Lox5-Lox4-Lox2 central group, and Post2-Post1 posterior group. In total, nine of the ten Hox genes are expressed during trochophore stage, indicating the full activation of this gene family, which is essential for the proper organization of the body plan.

## Discussion

**Figure 1:**
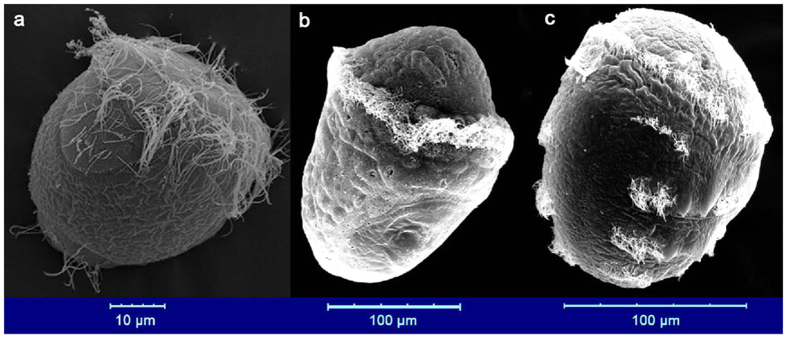
Electron microscopy images showing the trochophores. From left to right: The Pacific oyster, *Crassostrea gigas*, trochophore (14 hpf, in seawater at approximately 26 °C; photo from Zhang *et al*.^6^). The Pacific abalone, *Haliotis discus hannai*, trochophore (15 hpf, in seawater at approximately 22 °C). The sand worm *Perinereis aibuhitensis* trochophore (31 hours post-fertilization (hpf), in seawater at approximately 25 °C).

**Figure 2:**
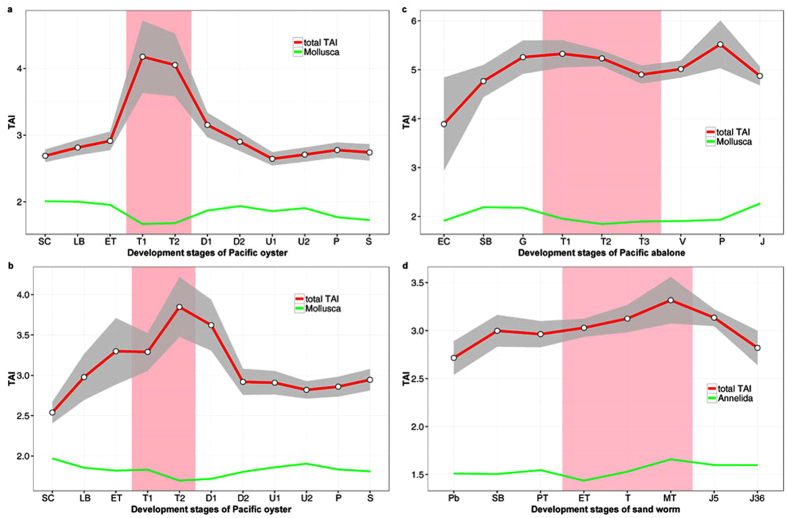
Transcriptome age indices across the ontogeny of the Pacific oyster, *Crassostrea gigas* (**a,b** indicate the two experimental replicates), the Pacific abalone, *Haliotis discus hannai* (**c**), and the sand worm *Perinereis aibuhitensis* (**d**). The red lines indicate the total cumulative value of the TAI. The green lines in panels a, b, and c show TAI for phylostrata older than Mollusca (ps1–ps9), while the green line in panel d represents TAI for phylostrata older than Annelida (ps1–ps9). The developmental stages and their timelines are defined in Supplementary Table S2–S5. The pink area designates the trochophore stage of each species. See the supplementary information for more details. The grey area designates the 95% confidence interval for TAI with a bootstrapping strategy. The overall patterns of the TAI profiles are significant as measured by the permutation tests (**a**. *P* = 2.1 × 10^−11^; **b**. *P* = 1.2 × 10^−10^; **c**. *P* = 3.3 × 10^−16^; **d**. *P* = 2.6 × 10^−6^).

Our study marks the trochophore as the phylotypic stage in both Mollusca and Annelida. In the three assayed species the lowest phylum-level TAI value was found during the trochophore stage, indicating this stage having the oldest transcriptome (Fig. 2 and Supplementary Fig. S1). During this stage, the shell field has formed in mollusks and the three body segments can be observed in sand worm (Fig. 1). As few studies have focused on phylotypic stage analyses in trochozoans, speculations have been primarily made based on the main body plan characteristics of the taxon. For instance, it has been proposed that the segmentation stage should be considered the phylotype for annelids, whereas the veliger is a candidate for the phylotype stage in mollusks^8^. However, other authors employing phenotypic analysis have proposed the trochophore as the phylotypic stage in mollusks^15^,^16^. In that line, high-density sampling and electron microscopy studies confirm that the molluscan shell field starts forming by trochophore stages^6^. Additionally, some annelid species form segments during the trochophore stage^21^. The timing at which these main phylum body plan characteristics arise is coincident with the observation that the lowest phylum-level TAIs occurred during the trochophore stage in the three trochozoans examined in this work. The highlighted similarities and low transcriptome polymorphism observed between the transcriptomes during the molluscs trochophore stage resembled the molecular hourglass model reported in vertebrates^9^, *Drosophila*^22^, *Caenorhabditis*^12^, plants^13^, and fungi^14^. A previous study on the expression pattern of oyster specific Homeobox genes, especially the observation that nine of the ten Hox cluster genes in oyster are expressed during trochophore stage, also suggested that the trochophore stage should be considered the phylotype^7^. However, the pattern for trochozoans we report here is less clear than in other organisms given that we found strong influence of some very young genes on the transcriptome composition in trochophores.

A recent report has indicated that transcriptome differences were minimal at trochophore stage in annelid *Platynereis dumerilii*, and thus this should be the phylotypic stage^16^. In this study, the highest levels of transcriptome similarity was also identified in the trochophore stage of the two mollusks. Among the common traits between mollusks and annelids, the trochophore larvae shows plesiomorphic characters such as the prototroch^23^,^24^. However, trochophore larvae can never the less be highly diverse^23^. In this study, the transcriptomes between the Annelida and the two mollusks did not show the highest similarity during the trochophore stage, which indicated substantial divergence between the two types of trochophores (Fig. 4). Indeed, the trochozoans are a group of animals showing wide body plan diversification, and some studies have suggested that many of the assembled features, such as the metatroch, have different embryonic origins or evolutionary histories^24^. The complex genomic context of trochozoans includes high rates of genome polymorphism and large amounts of repeated content, making the molecular evolution of trochozoans intricate^25^. The transcriptome divergence of trochophore between the two phylum (Mollusca and Annelida) may be a result of integrating new genes into regulatory networks through long-term evolution. The differences in trochophore transcriptomes between these two closely related phyla resembles the inverse hourglass pattern observed between other more distant animal phyla^16^. In this regard it was suggested that ‘phylum’ could be defined as a set of species sharing the phylogenetically conserved molecular regulatory network during the mid-development^16^.

**Figure 3:**
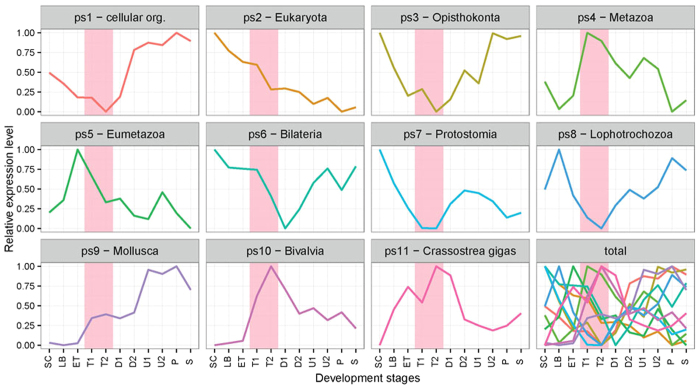
The mean relative expression levels (REL) of genes in each phylostratum. Phylostratum name is shown at the top label of each panel. The phylostrata (ps) 1, 3, 6, 8 relatively highly expressed at late development, while ps 4, 5, 10, 11 relatively lowly expressed at late development. Pink shaded area designates trochophore stage when the two types of phylostrata have the largest difference (the panel marked with “total”).

**Figure 4:**
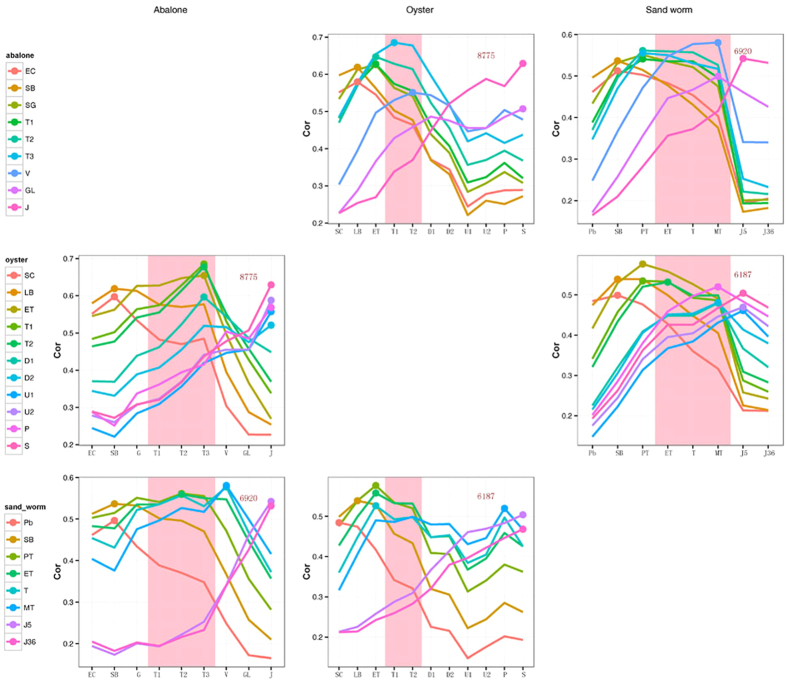
Transcriptome similarities of the different development stages between species. The Spearman correlation coefficients (ρ) of the transcriptome profiles of the different development stages were calculated in a pairwise manner between species. The x-axis indicates the development stages stated in the corresponding column header. The differently colored lines indicate the different development stages of the species stated in the corresponding row header. The pink area designates trochophore stage. The highest ρ value throughout the developmental stages is indicated by a point on the corresponding line. The numbers in each panel show the gene numbers used for the analysis.

**Figure 5:**
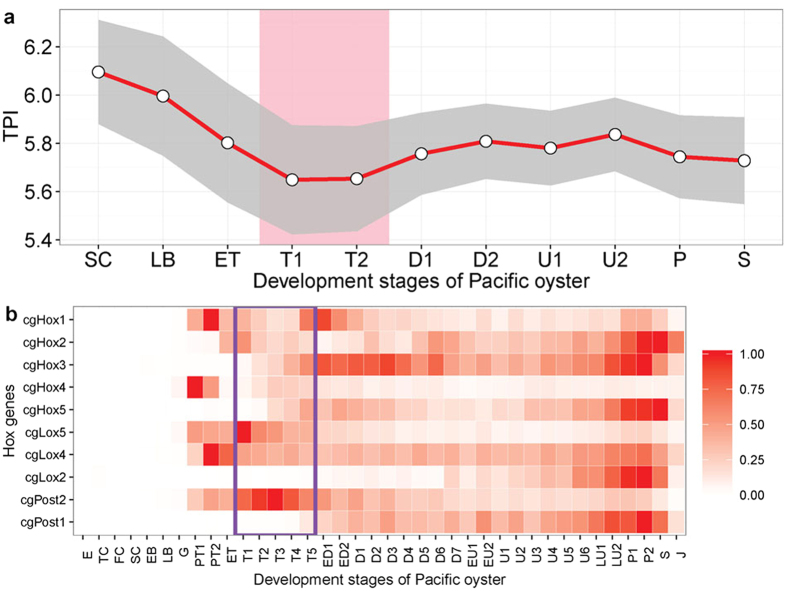
The transcriptome diversity indices over embryo stages (**a**), and the expression pattern of oyster Hox family genes (**b**). (**a**) The transcriptome diversity indices (TPI) were calculated based on the expression and the nucleotide diversity of the genes among 10 individuals. The area between pink lines designates the trochophore stage. The grey area designates the 95% confidence interval for TPI with a bootstrapping strategy. The overall pattern of the TPI profile is significant as measured by the permutation tests (*P* = 5.3 × 10^−38^). Pink shaded area designates trochophore stage. (**b**) RPKM Expression values were normalized between 0 and 1 for each gene across all stages. A value of 0 indicates no/lowest express and 1 indicates the stage where each gene was maximally expressed. Purple rectangle designates the trochophore stages.

While phylum level TAI and transcriptome similarity analyses reveal the transcriptome characteristics of genes widely present in multiple species, new genes may also play key roles in developmental plasticity and evolution of novel body plans^26^. In this study, “new genes” were defined as those that are mapped to the shallowest node on the phylogeny used in the phylostratigraphic analyses^26^,^27^. For TAI analysis, these genes were assigned to ps11 in oyster and abalone, and ps 10 in sand worm. These new genes were found to have the highest expression levels in trochophore stage (Fig. 3), indicating the high degree of regulation of the activity of new genes during this stage. A phylostratigraphic analysis of oyster miRNAs also showed that new miRNAs were also highly expressed during the trochophore stage^28^. One possible explanation is that new genes arise as a result of the adaptive change of the species in response to the environment^29^. As a link between the gastrula and adult-like stages (the veliger in mollusks and the nectochaete in annelids), the trochophore may be an important stage determining the development of the adult body plan. Furthermore, the population dispersion of benthic marine animals mainly relies on swimming larvae^30^. Thus, the larvae will encounter more complex environments, and the high expression of new genes during trochophore could be the result of both the need of environmental adaption and full development of adult-like organs at the larvae stage. Indeed, the development of juvenile structures in larvae is believed to be one of the most important adaptive mechanisms for the rapid metamorphosis of marine invertebrate larvae^31^. These findings support the view that evolutionary processes in the stem of a phylum should be investigated by assessing the wide range of extant species within the phylum^32^, as expression patterns of individual species could give a biased picture.

Phylo-transcriptomic index used in this study (TAI) rely on the phylostratigraphic approach which maps genes on the consensus phylogeny based on the sequence similarity inference using BLAST^10^,^27^. Two recent papers questioned reliability of the BLAST algorithm in phylostratigraphic analyses by claiming that in tracing remote homologs false negative BLAST error rate, which they estimated at 15%, could impact phylostratigraphic inference^33^,^34^. Interestingly, a reanalyses of these simulation studies found unrealistic parameter choices, irreproducibility, statistical flaws and partial representation of results which all together suggest that BLAST is an appropriate and sufficiently sensitive tool in phylostratigraphic analysis^35^. However, it is important to understand that phylostratigraphic approach is based on the model of punctuated evolution of protein families and is therefore designed to trace significant shifts in protein sequence, which are relevant for biological adaptations, and not remote homologs^35^,^36^,^37^. Therefore, remote homolog BLAST error rates, regardless of their magnitude, are not directly relevant for TAI calculations which rely on gene age estimates according to the model of punctuated evolution of protein families^35^.

The larval transition from the trochophore to the D-shaped veliger stage is probably the most dramatic event to occur during the development of oysters. During this transition, most of the organs form in approximately 10 hours. The footprint of this transition could be clearly seen at the expression levels when the detailed contributions of genes from all phylostrata were simultaneously depicted. In this context, it was also indicative that the expression of Bivalvia- (ps10) and oyster-specific genes (ps11) peaked at the trochophore stage, as well as the relative expression levels (REL) for each phylostratum experienced significant change during the trochophore stage (Fig. 3). However, more detailed studies on the gene functions are needed to bridge this general trend of the transcriptome and the real developmental mechanisms and morphological outcomes.

The expression waves of phylostrata-specific genes are less conclusive in relation to a long-standing debate about the origin of indirect development in marine invertebrates. According to the ‘larvae-first’ hypothesis, the original metazoan life cycle included pelagic adults, which were similar to the modern trochophore larvae^4^. An opposing view is presented by the ‘intercalation’ hypothesis, which proposes that the pelagic larvae evolved secondarily in different phyla and became intercalated multiple times into a life cycle that was originally direct^5^,^38^. Our analysis shows elements of both scenarios. For instance, the exclusive wave of Metazoa-specific genes (ps4) during the trochophore stage suggests that this larvae relies on ancient processes in accordance with ‘larvae-first’ scenario. However, the high expression of phylum-, class- and species-specific genes (ps9, ps10 & 11) during the trochophore stage suggests massive recent adaptive elaborations that go in favor of the ‘intercalation’ idea. These elements may be the main cause of phylum level difference (Annelida vs. Mollusca) between the trochophore larvae. As marine larvae bear both development and population dispersal function their evolutionary history might be far more complex than the simple ‘intercalation’ vs. ‘early larvae’ hypotheses dichotomy could explain.

## Methods

### Sampling and developmental staging

The Pacific oyster *Crassostrea gigas* was sampled in two parallel experiments. The RNA-seq data from one replicate have previously been reported^6^. We designed the second replicate according to the main stages sampled previously^39^. Briefly, the oysters used for the first experiment were the F_2_ mass-mating progenies of 51 females and 1 male from the “G3” family, which was constructed from 1 farm-cultured female and 1 farm-cultured male that were collected from Yantai, China. As the sampling time intervals were short in this experiment, the transcriptome age indices of some adjacent stages were similar. We thus only chose a few typical stages in this study, which were the sixteen cells (SC), late blastula (LB), early trochophore (ET), two trochophore stages (T1 & T2), two D-shape stages (D1 & D2), two umbo larvae stages (U1 & U2), the pediveliger (P) and the spat (S) (see Supplementary Table S2). The oysters used for the second experiment were F_1_ progenies produced by individuals from a wild population around Zhangzidao Island, Liaoning, China. Twenty females and one male were used. The embryos and larvae were cultured at 22 °C in seawater. The same developmental stages were sampled as in the first repeat (see Supplementary Table S3). The gamete collection, insemination, larval culturing, and sampling methods used for RNA extraction and electronic microscopy examination were the same as those employed in a previous report^6^.

The gonads of mature Pacific abalone *Haliotis discus hannai* were induced at the Laodong Aquaculture Breeding Company, Qingdao, China, via air exposure and using sand-filtered ultraviolet-irradiated seawater, as described previously^40^. Each induced individual was then placed separately into a 10 L bucket for spawning. The eggs from ten females were inseminated with the sperm from ten males. The embryos and larvae were cultured at 22 °C in seawater using the previously described methods^40^. Staging was conducted on-site under light microscopy according to a previous report^41^ and was confirmed through electronic microscopy examination (see Supplementary Fig. S5). The details for each of the sampled developmental stages are listed in Supplementary Table S4.

The gonads of mature and spawn-ready sand worms *Perinereis aibuhitensis* were collected from a sand worm culture farm at Rushan, Weihai, China. The matured worms were dry-transported at a low temperature to the aquarium of the Institute of Oceanology, Chinese Academy of Sciences. Approximately 30 worms were then placed into a bucket containing 30 L of 25 °C seawater. The worms were spawned soon after they were placed into the seawater. The mixture of eggs and sperm was collected every 10 min by transferring the worms into a new bucket. Thus, a total of three buckets of zygotes were produced with developmental age intervals of approximately 10 min. Developmental staging was conducted on-site through light microscopy according to a previous report^42^,^43^ and was confirmed via electronic microscopy examination of representative embryos (see Supplementary Fig. S6). The details for each of the sampled developmental stages are listed in Supplementary Table S5. As sand worm eggs have thick envelopes, the volume of the zygote is quite large. As a result, a normal 1.5-mL centrifuge tube can only be used to collect a few zygotes, and the thick envelope has a negative influence on RNA extraction. To solve this problem, we first removed the egg envelopes and then extracted RNA from fresh samples. Briefly, approximately 1.2 mL of an embryonic or larval sample was first mixed with 0.2 mL of guanidinium thiocyanate-phenol-chloroform (TRIzol, Invitrogen, Beijing, China). The samples were then collected immediately by centrifugation (8,000 × *g*) for 30 s. Four tubes of samples were treated at the same time to ensure sufficient quantity of RNA. The embryos or larvae were then released from the envelope and precipitated to the bottom of the centrifuge tube. After the envelope was removed, a portion of the retained embryos or larvae was sampled for electronic microscopy examination. The RNA of the remaining embryos or larvae was extracted using TRIzol according to the manufacturer’s protocol.

To analyze the DNA polymorphism and variance of the oyster genes, we conducted genome re-sequencing in ten individuals. The oysters were collected from different sites in northern China, and DNA was isolated from the gills of each individual using standard molecular biology techniques.

### Illumina-based RNA and DNA sequencing

Because there are no reference transcriptomes for Pacific abalone and sand worms, we conducted paired-end RNA-seq on the two species and assembled their transcriptomes. The transcriptome of the Pacific abalone was sequenced using the Illumina Genome Analyzer IIx platform at Biomarker Technologies CO., LTD, while that of the sand worm was sequenced using the Illumina HiSeq 2000 platform at BGI-Shenzhen. RNAs from each developmental stage were first mixed in equal quantities. Then, sequencing libraries were constructed according to the Illumina’s recommended protocol.

Single-end RNA-seq was conducted to obtain the number of gene reads for each developmental stage of the three species. Single-end RNA sequencing data for the oyster and abalone samples was generated using the Illumina Cluster Station and Illumina Genome Analyzer IIx, while the sand worm was sequenced using the Illumina HiSeq 2000 platform. The sequencing libraries were constructed according to Illumina’s recommended protocol.

A short insert library with 300-bp fragments was constructed for resequencing of the oyster genome using a standard protocol recommended by Illumina. The DNA was sequenced using the Illumina HiSeq 2000 platform.

### Transcriptomic analysis and SNP calling

RNA-seq reads can be from any site within the total mRNA, in contrast to the fixed tag detection employed in microarrays. Thus, we needed to normalize the number of reads by dividing the reads by the length of the count region. This allows the normalized number of reads to be regarded as representative of the absolute number of gene transcript molecules. At the same time, the trimmed mean of M values (TMM) normalization was applied to adjust for the RNA composition effect^44^. To date, most global gene expression analyses based on RNA-seq have assumed that the total mRNA abundance does not differ between different experimental samples. The problem with this assumption is that mRNA abundance is not equal, as expected. This assumption is irrelevant to TAI analysis, which only investigates how the partial concentrations of individual genes contribute to the overall expression levels in the transcriptome. However, the TAI value is sensitive to a few highly abundant mRNAs. The partial concentrations of transcripts of certain genes can account for more than 1% of the assayed transcriptome. When the expression level fluctuates across developmental stages, the TAI pattern is highly influenced by these extreme genes. We found that in the oyster, some genes showed very similar expression patterns (with Pearson’s correlation coefficients greater than 0.99) and fluctuating expression curves during the course of ontogeny. These genes had short nucleotide sequences (186–465 bp) and extremely high expression levels in most samples (RPKM was as high as 120,000). These genes were therefore considered to be special genomic elements such as retrotransposons. In this case, we only retained one gene among each cluster. As a result, a total of 13 genes were discarded in this step (see Supplementary Fig. S7). Furthermore, some genes may be duplicated and showed similar nucleotide sequences, we thus filtered the redundant genes out of version 9 of the oyster genome (released in 2012)^6^. To construct the non-redundant gene set, BLAT^45^ alignment searches were performed on the CDS and protein sets using the sets themselves as separate queries. The gene clusters were then constructed based on the similar sequences and were visualized using Cytosape^46^. The following criteria were used to determine the similar genes: 1. the protein sequences shared at least 97% identity in the alignment region, while the DNA sequences shared at least 95% identity; and 2. there were no gaps in the aligned protein regions. As a result, 505 genes were discarded because their sequences were redundant (see Supplementary Fig. S8). The filtering of these genes did not change the main TAI pattern compared with that using the whole predicted gene set and all the sampled development stages (see Supplementary Fig. S4), but reduced the high impact of few abundant short questionable genes.

For the Pacific abalone and sand worm, we assembled the transcriptomes with the Trinity program^47^. The transcript abundances were estimated using RSEM software^48^ based on the single-end read alignment obtained in Bowtie2^49^. The transcript abundances for the oyster genes were calculated using a previously described method^6^. Briefly, the reads were mapped using Tophat^50^, and the mapped reads for every gene were calculated using a Perl script developed in-house. The data for the hit reads can be found in Supplementary Table S6.

The sequenced reads from the genomes of ten individual oysters were mapped onto the oyster genome using Bowtie2^49^. A combination of SAMtools and BCFtools^51^ was used in SNP calling and allelic genotyping. The diploid genome sequences from each individual were inferred using the genomic allele site information. Then the gene sequences for each individual were extracted according to the oyster gene model annotation, and were used as the input for the DnaSP software^52^ to calculate the value of theta per nucleotide site (*θ*, see Supplementary Table S7).

### TAI and TPI

A database containing the complete genomes of 3,915 species was constructed (see Supplementary Table S8). The species were sorted into 11 phylostrata for the TAI analysis of the oysters and abalone and 10 phylostrata for the TAI analysis of the sand worm (see Supplementary Fig. S9). Blastp searches were conducted against the database using oyster proteins, while Blastx searches were conducted using the unigenes of abalones and sand worms.

The transcriptome age index of developmental stage *s* (TAI*s*) was calculated according to the method reported by Domazet-Lošo and Tautz^10^:


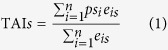


where *ps*_*i*_ is the evolutionary age (phylostratum) of gene *i*; *e*_*is*_ is the weighted read number of gene *i* at stage *s*, which is weighted by the CDS length of the gene; and *n* is the total number of genes analyzed.

To calculate the relative expression levels (REL) of the genes for a given phylostratum (*ps*) and developmental stage (*s*), we first measured the partial concentration (*f*_*is*_) according to the formula *f*_*is*_ = *e*_*is*_/(*e*_*1*_ + *e*_*2*_ + … + *e*_*n*_). Then, RE was calculated according to the following equation:


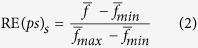


where *f* is the average partial concentration of genes from phylostratum *ps* for a given stage, *s*; and 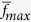 and 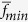 are the maximal and minimal average partial concentrations from phylostratum *ps* across all stages considered, respectively.

As oysters show a high degree of polymorphism, the SNP content varied for the different oyster genes. Some genes rarely exhibited SNP variations, indicating purifying selection during evolution. However, there was a high proportion of segregating (polymorphic) sites among some other genes. We calculated the *θ* for each gene and introduced the transcriptome polymorphism index (TPI), which was calculated according to the following equation:


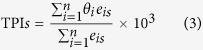


where *θ*_*i*_ is the theta per nucleotide site of gene *i*. When multiplying by 10^3^, the value is theta per kilo nucleotide sites.

### Other analysis methods

The orthologs were determined between each pair of species based on a reciprocal best-hit Blast with an E-value cutoff of 1e-5. Spearman’s correlation coefficient (ρ) was used to evaluate transcriptome similarity. The statistical analyses and plots were performed and generated in R (http://www.R-project.org/)^53^. A 1000 replication bootstrapping strategy was used to create a 95% confidence interval for TAI and TPI values with the “boot” R package. To determine the statistical significance of TAI and TPI pattern, permutation test was performed. The three components (phylostratum in TAI or theta value in TPI, gene length, and RPKM) of each gene were randomly permuted 1000 times within each developmental stage *s*. In this way, 1000 TAI*s* surrogate values were calculated for each development stage. These values were approximated by gamma distribution, and two parameters (shape and scale) were estimated by the method of moments. The fitted gamma distribution was treated as null distribution, and the *P*-value of observed TAI*s* was computed from the null distribution. Then the combined *P*-value for the TAI pattern was computed using the meta-analytic approach^54^. TPI followed the same procedure.

## Additional Information

**Accession codes:** The transcriptome, RNA-seq and genome re-sequencing raw data have been deposited with GenBank under BioProject SRP056090.

**How to cite this article**: Xu, F. *et al*. High expression of new genes in trochophore enlightening the ontogeny and evolution of trochozoans. *Sci. Rep.* **6**, 34664; doi: 10.1038/srep34664 (2016).

## Supplementary Material

Supplementary Information

Supplementary Table S6

Supplementary Table S7
