# Supplementary material for: High expression of new genes in trochophore enlightening the ontogeny and evolution of trochozoans
Source: Sci Rep. 2016 Oct 4;6:34664. doi: 10.1038/srep34664 (PMC5048140; doi:10.1038/srep34664)
Supplement: Supplementary Information [file srep34664-s1.pdf]

Supplementary Information for

High expression of new genes in trochophore enlightening the ontogeny and evolution of trochozoans

Fei Xu[1,2,3], Tomislav Domazet-Lošo[4,5], Dingding Fan[6], Thomas L. Dunwell[7], Li Li[1,2,3], Xiaodong Fang[6], Guofan Zhang[1,2,3#]

[1]Key Laboratory of Experimental Marine Biology, Institute of Oceanology, Chinese Academy of Sciences, Qingdao 266071, China

[2]Laboratory for Marine Biology and Biotechnology, Qingdao National Laboratory for Marine Science and Technology, Qingdao 266071, China

[3]National & Local Joint Engineering Laboratory of Ecological Mariculture, Institute of Oceanology, Chinese Academy of Sciences, Qingdao 266071, China

[4]Laboratory of Evolutionary Genetics, Ruđer Bošković Institute, Bijenička cesta 54, P.P. 180, HR-10002, Zagreb, Croatia.

[5]Catholic university of Croatia, Ilica 242, HR-10000, Zagreb, Croatia

[6]BGI-Shenzhen, Shenzhen, 518083, China.

[7]Department of Zoology, University of Oxford, South Parks Road, Oxford, OX1 3PS UK.

#Corresponding author: G.Z. (gzhang@qdio.ac.cn).

This file includes:

Supplementary figures S1-S9

Supplementary tables S1-S8

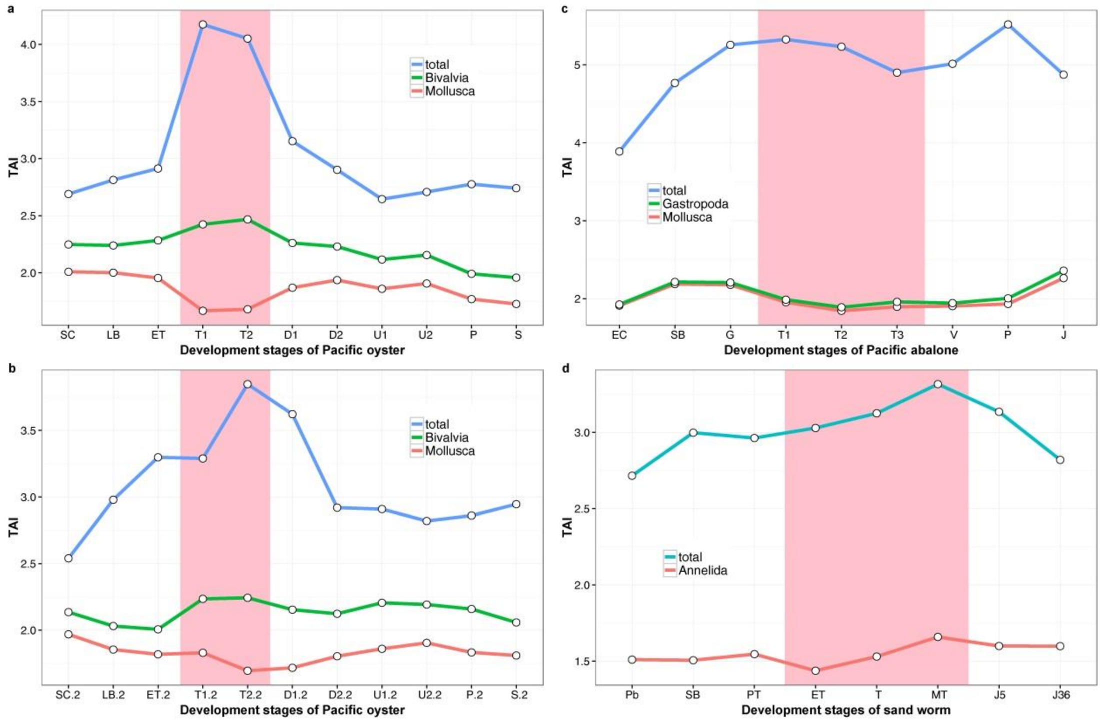

Figure S1 | **Transcriptome age indices across the ontogeny of the Pacific oyster, *Crassostrea gigas* (a and b indicate the two experimental replicates), the Pacific abalone, *Haliotis discus hannai* (c), and the sand worm *Perinereis aibuhitensis* (d).** The blue lines indicate the total cumulative value of the TAI. The green lines in panels a and b show TAI calculated for phylostrata older than Bivalvia (ps1-ps10). The green lines in panel c represent TAI calculated for phylostrata older than Gastropoda (ps1-ps10). The red lines in panels a, b, and c show TAI for phylostrata older than Mollusca (ps1-ps9), while the red line in panel d represents TAI for phylostrata older than Annelida (ps1-ps9). The developmental stages and their timelines are defined in Table S1-S4. The area with pink background designates the trochophore stage of each species.

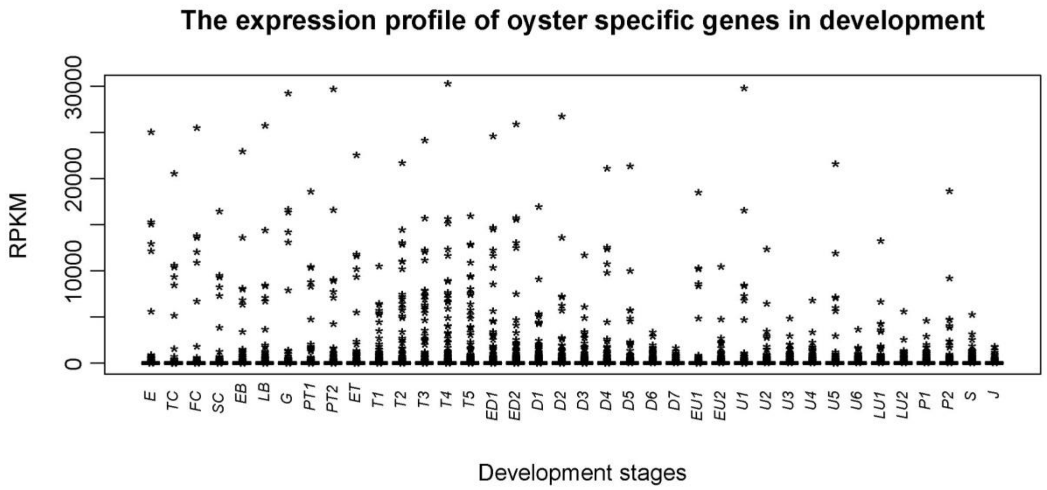

Figure S2 | **Distribution of RPKM value of all the annotated genes in the genome across different development stages**. Some genes showed high expression level at trochophore stage, mainly including 51 genes from ps11 (oyster-specific) and 5 genes from ps10 (Bivalvia-specific). Details of these 56 genes were shown in Table S1.

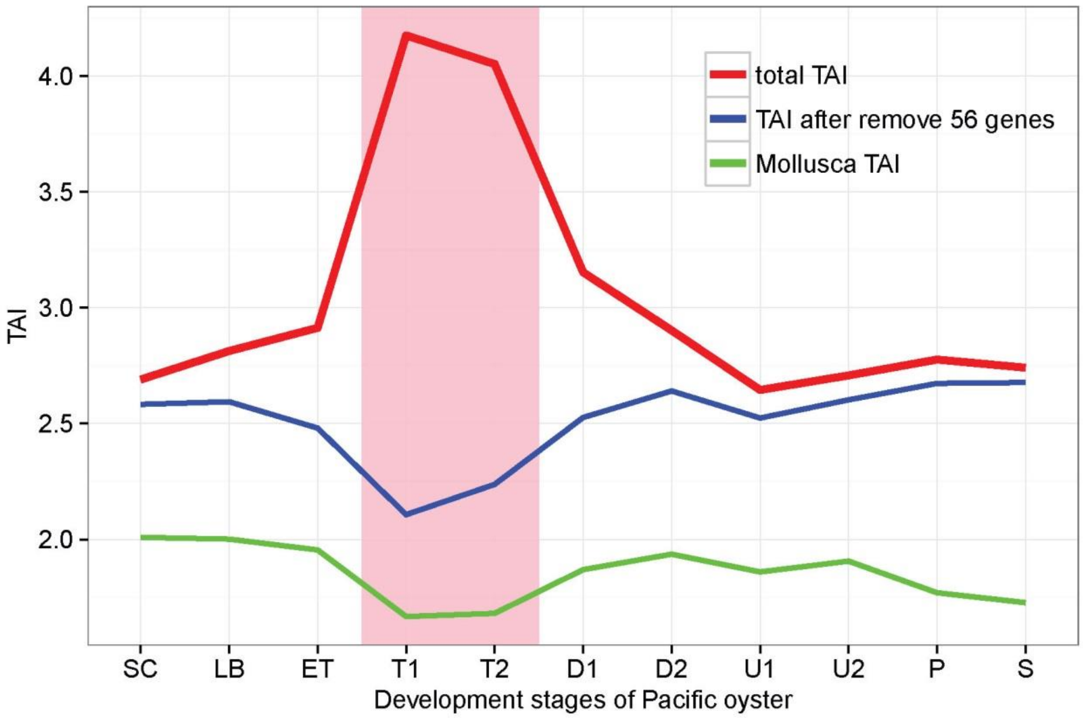

Figure S3 | **Dissection of the TAI composition by isolating the 56 genes (shown in Figure S3 and Table S1) mainly contributing to the high TAI value at trochophore stage**.

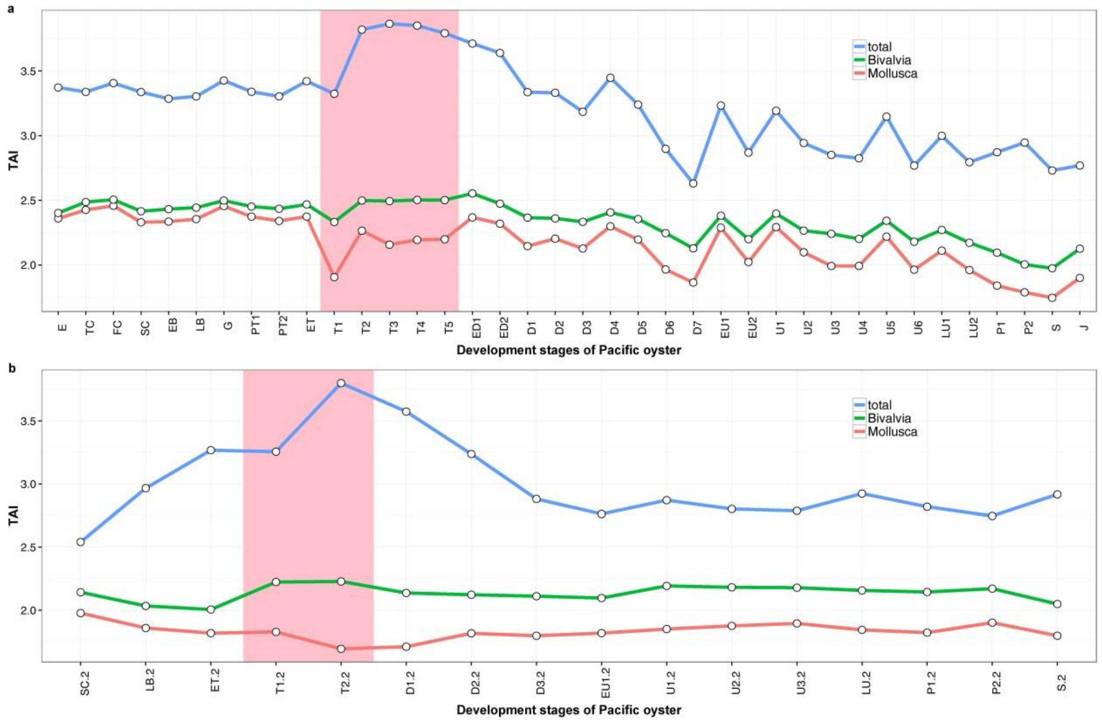

Figure S4 | **Transcriptome age indices across the ontogeny of the Pacific oyster, *Crassostrea gigas*.** TAI values for all the sequenced development stages were calculated using all the predicted genes of each genome genome. It can be seen that the selected typical development stages shown in Figure S1a & S1b can well reflect the pattern in this figure.

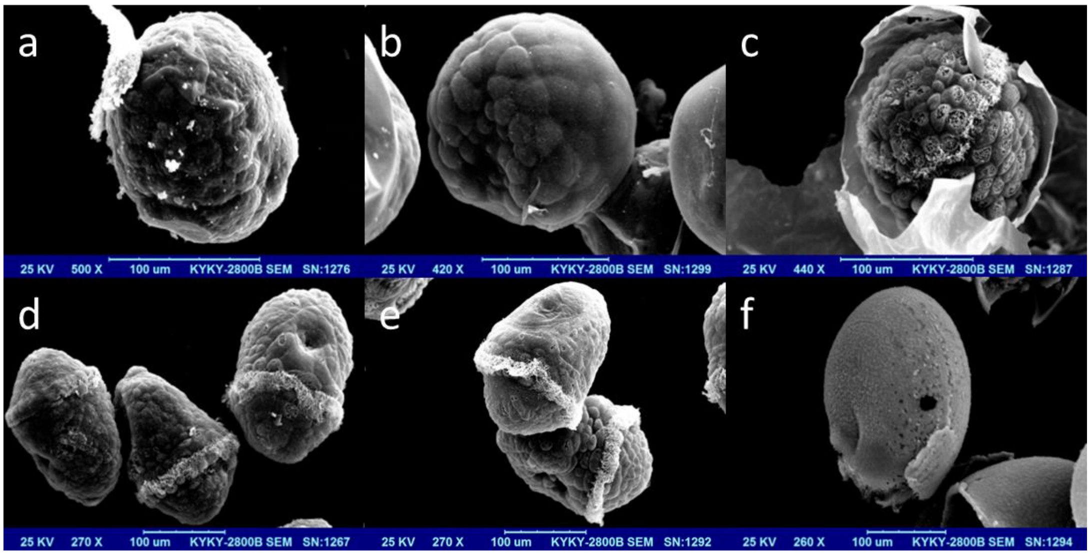

Figure S5 | **The embryonic and larval development of Pacific abalone *Haliotis discus hannai*.**

a. Stereoblastula stage at about 200 min post fertilization. **b**. Gastrula at 6 h post fertilization. **c**. Trochophore at 10.5 h post fertilization. The larvae was still in the egg envelope. **d**. Trochophore at 13.5 h post fertilization. Free swimming larvae hatched, and the shell field formed. **e**. Early veliger larvae at 15 h post fertilization. The shell field expanded at the dorsal side of the larvae. **f**. Veliger larvae at 25 h post fertilization. The calcified adult-like shell formed.

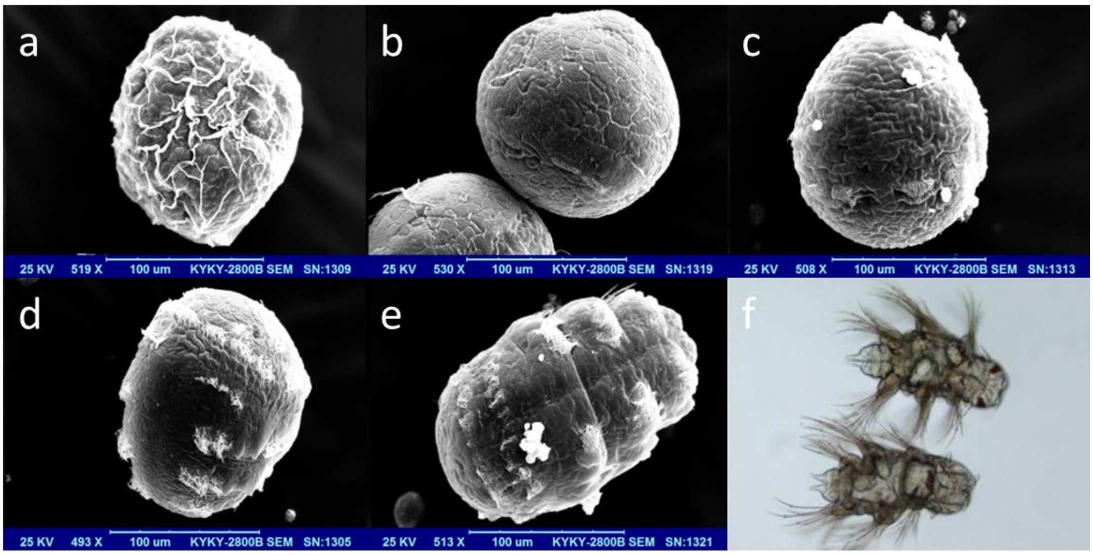

Figure S6 | **The embryonic and larval development of sand worm *Perinereis aibuhitensis*.**

a. Stereoblastula stage at 8 h post fertilization. The white lines at the surface of the embryo were wrinkles formed by the jelly around the embryo. **b**. Protrochophore at 14 h post fertilization. **c**. Trochophore at 26 h post fertilization. The larvae was still in the jelly. The prototroch formed at this stage. **d**. Metatrochophore at 30.5 h post fertilization. Three segments are visible, and the chaetae formed in the trunk. **e**. Juvenile in the jelly with three setigers (or metatrochophore), 36 h post fertilization. **f**. Juvenile with three setigers (or nectochaete) at 2.5 d post fertilization. This photo was taken with the light microscope.

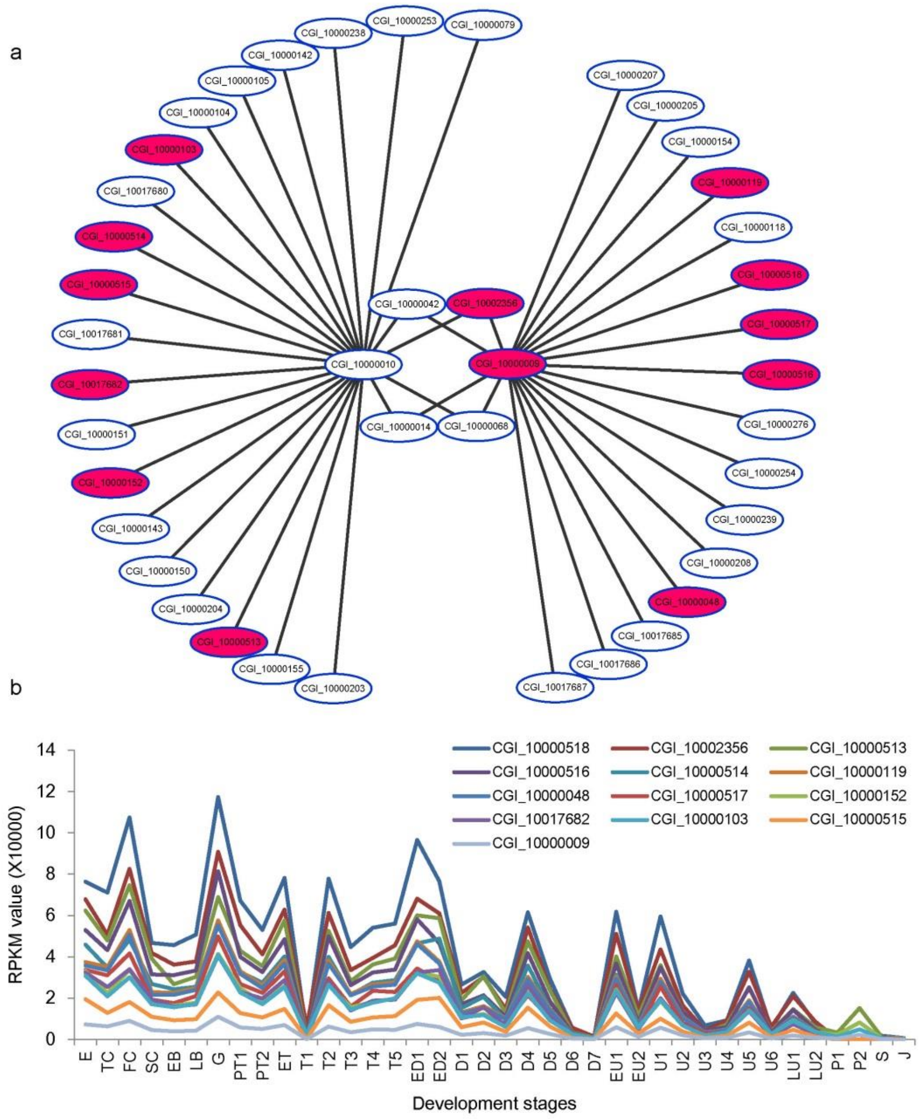

Figure S7 | **Genes filtered out through the expression pattern**.

Thirteen additional genes were discarded (the red color filled ellipses in panel a, and the genes listed in panel b) during the TAI analysis. These genes showed very similar expression pattern (Pearson correlation coefficients were larger than 0.99, panel a & b) and fluctuating expression curve along the ontogeny (panel b). They had short nucleotide sequences (186-465 bp) and extremely high expression levels in most samples (RPKM was as high as 120,000). These genes were thus considered to be some kind of special genomic elements, such as retrotransposon.

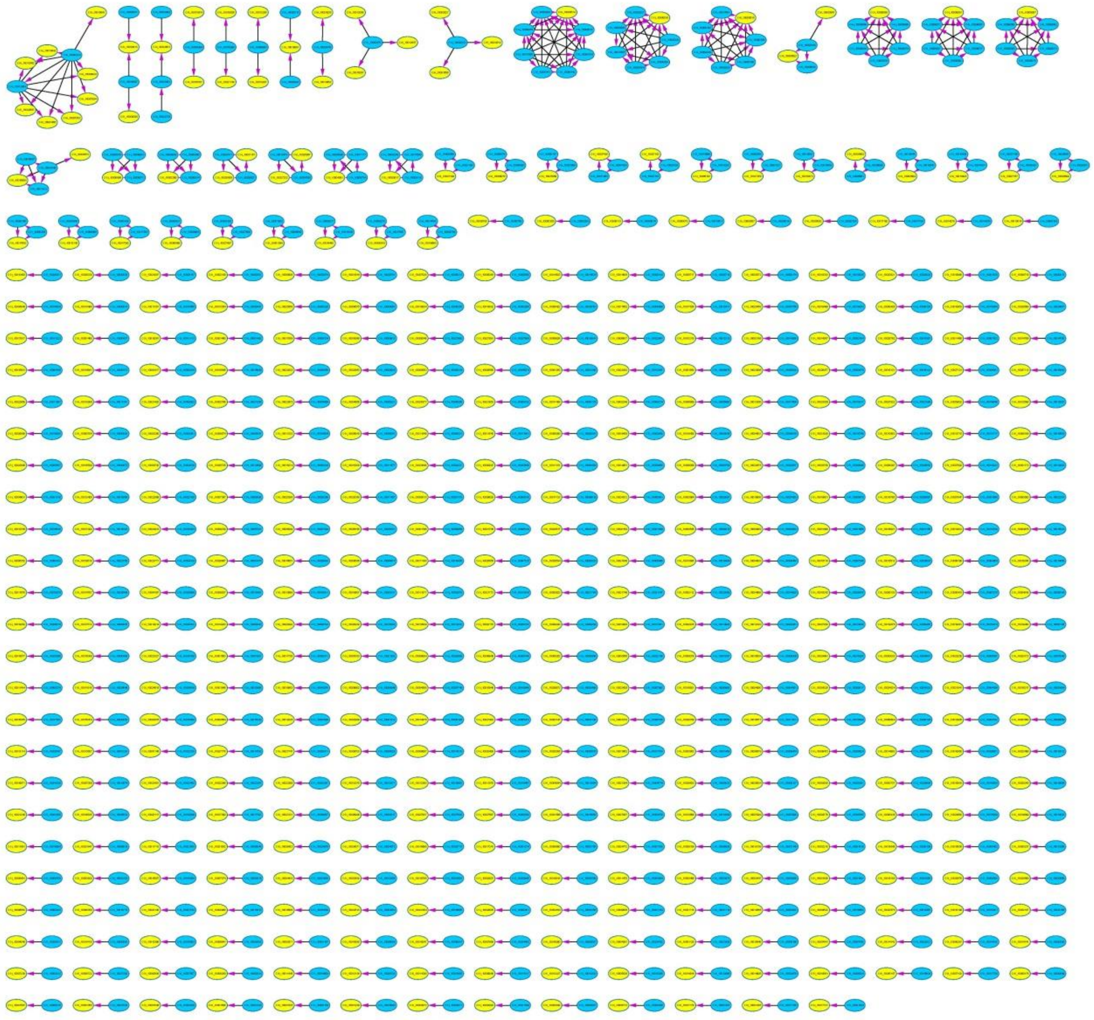

Figure S8 | **Genes filtered out through sequence redundant analysis**.
The ellipse filled with yellow color shows the genes which were retained, while the blue filled color shows the genes which were discarded. The target (the ellipse arrow is pointing) was defined as the genes with equal or larger length than the source (the ellipse at the other side of the arrowed line). In this case, all the source genes were discarded, and only the gene which was target for all the related genes (there are only arrow point around the gene) was retained. The following criteria were used to identify redundant genes: 1. When the query and target genes had different length, the shorter one should aligned onto the longer one completely. 2. The protein sequences should be at least 97% identities in the alignment region, while the DNA sequences should be at least 95%. 3. There should be not any gap in the aligned protein region.

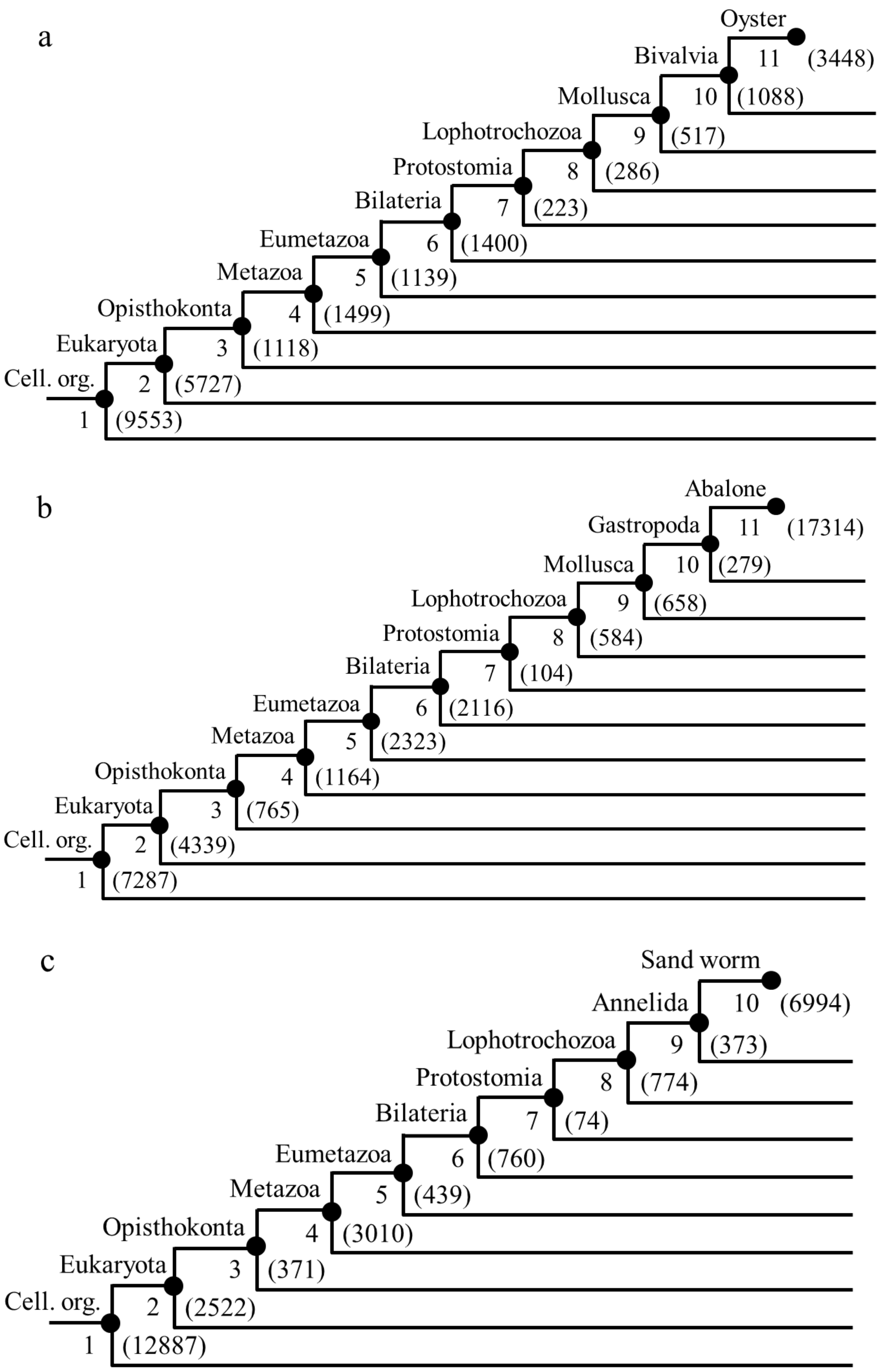

Figure S9 | **Phylostratigraphic map of oyster (a), abalone (b) and sand worm (c)**. The numbers at the lower left of each spot denote the number phylostrata, while the numbers in parenthesis at the lower right denote the number of genes per phylostratum. Cell. org., cellular organisms.

Table S1 | **Details of the 56 genes highly expressed in trochophore stages, and mainly contributing to the high value of the total TAI**. "NA" in the IPR, GO, KEGG, TrEMBL columns indicate the corresponding annotation is not available. Value in each development stage is the TAI value of each gene in the corresponding development stage (calculated by $ps_i f_i$, see reference 10 in the main text for more details).

Gene ID	theta	IPR	GO	KEGG	TrEMBL	Length (bp)	Age (ps)	SC	LB	ET	T1	T2	D1	D2	U1	U2	P	S
CGI_10006331	0.003223	NA	NA	NA	NA	1137	11	0	7.25E-06	0.000125	0.003297	0.002794	0.000697	0.000297	9.67E-05	7.82E-05	4.45E-05	1.60E-05
CGI_10003033	NA	NA	NA	NA	NA	771	11	1.76E-06	4.28E-06	0.000315	0.003359	0.003619	0.003271	0.001699	0.000813	0.000533	0	0.000198
CGI_10025597	0.014716	NA	NA	NA	NA	747	11	0.000104	3.53E-05	0.000238	0.003367	0.002464	0.001024	0.00041	0.0002	0.000145	0.000101	3.40E-05
CGI_10022474	NA	NA	NA	NA	NA	228	11	0.001036	0.0036	0.007911	0.003432	0.003281	0.002826	0.001458	0.001913	0.001697	0.002027	0.002055
CGI_10000269	0.008542	NA	NA	NA	NA	264	11	1.03E-05	5.62E-05	0.000539	0.003432	0.003466	0.000605	0.000197	4.43E-06	0	0	6.88E-06
CGI_10013629	0.011745	NA	NA	NA	NA	264	11	0.004138	0.00216	0.004225	0.003556	0.001489	0.00087	0.001139	0.00058	0.000937	0.001696	0.003027
CGI_10023289	0.001362	NA	NA	NA	NA	621	11	0	2.65E-06	0.001777	0.003671	0.004264	0.002558	0.003561	0.000573	0.000491	0.001006	0.00048
CGI_10004597	0.005369	NA	NA	NA	NA	315	11	0.000965	0.001769	0.004125	0.003685	0.003667	0.002989	0.001826	0.001619	0.001724	0.000951	0.000963
CGI_10005680	0.004651	NA	NA	NA	NA	606	11	3.36E-05	4.62E-05	0.000299	0.003777	0.008682	0.007638	0.010906	0.003347	0.004108	0.003622	0.003407
CGI_10022167	0.004153	NA	NA	NA	NA	543	11	2.50E-06	0	6.56E-05	0.003927	0.006049	0.001027	0.001113	0.000379	0.000296	0.000183	9.03E-05
CGI_10025265	0.012723	NA	NA	NA	NA	288	11	0.004925	0.007132	0.006252	0.003957	0.004531	0.002432	0.002098	0.001714	0.001902	0.001347	0.002617
CGI_10018867	0.005066	NA	NA	NA	NA	612	11	7.76E-05	5.39E-05	0.000974	0.004121	0.001012	0.00044	0.00043	0.000564	0.000569	0.001734	0.001163
CGI_10003505	0.011564	NA	NA	NA	NA	390	11	0.017233	0.020647	0.007469	0.004126	0.002346	0.00245	0.001342	5.10E-05	3.70E-05	2.78E-05	6.52E-05
CGI_10024593	0.004732	NA	NA	NA	NA	417	11	0	0	0.000114	0.004145	0.004781	0.001384	0.000402	0.000485	0.000248	0.000922	8.71E-06
CGI_10002843	NA	NA	NA	NA	NA	384	11	7.07E-06	8.59E-06	0.00061	0.004233	0.005401	0.018161	0.023504	0.007934	0.00117	0	8.51E-05
CGI_10027216	0.021667	NA	NA	NA	NA	1392	11	2.93E-06	2.37E-06	4.48E-05	0.004594	0.011136	0.010535	0.001786	0.000302	0.00049	0.002628	0.000301
CGI_10027684	0.01188	NA	NA	NA	NA	261	11	0	0.000221	0.003364	0.00502	0.003626	0.002823	0.000576	0.000314	0.000166	0.000228	0.000118
CGI_10014829	NA	NA	NA	NA	NA	327	11	0.013335	0.013838	0.00655	0.005142	0.00532	0.001602	0.001529	0.003391	0.004093	0.00016	0.003482
CGI_10013300	0.01439	NA	NA	NA	NA	333	11	4.08E-06	0	5.34E-05	0.005295	0.007983	0.005713	0.0025	0.000688	0.000613	0.000282	1.64E-05
CGI_10021124	0.01634	NA	NA	NA	NA	207	11	0.000354	0.005017	0.005446	0.005303	0.017499	0.014199	0.016676	0.004983	0.003343	0.005153	0.004755
CGI_10015394	0.008654	NA	NA	NA	NA	456	11	0.007182	0.006337	0.004996	0.005665	0.007974	0.009745	0.008312	0.015228	0.011693	0.01463	0.00906

CGI_10023434	0.005338	NA	NA	NA	NA	264	11	0.002226	0.004121	0.005562	0.005777	0.005509	0.004702	0.003658	0.00198	0.002075	0.002844	0.002084
CGI_10013302	0.012986	NA	NA	NA	NA	369	11	4.78E-05	0.000277	0.000981	0.006057	0.007374	0.000995	0.000266	0	6.51E-06	1.47E-05	9.84E-06
CGI_10002107	0.001515	NA	NA	NA	NA	372	11	0.009285	0.00588	0.008955	0.006065	0.005986	0.004459	0.004173	0.002773	0.002519	0.00231	0.003188
CGI_10020278	0.006909	NA	NA	NA	NA	204	11	0.009913	0.042923	0.020111	0.006159	0.002286	0.000852	0.000312	4.59E-05	0	8.85E-06	8.90E-06
CGI_10008847	0.004503	NA	NA	NA	NA	3693	11	6.25E-06	2.68E-06	0.002293	0.00655	0.002719	0.001382	0.000726	0.000275	0.000239	9.38E-05	6.88E-06
CGI_10000377	0.004836	NA	NA	NA	NA	408	11	0.011343	0.039344	0.019835	0.006589	0.002209	0.000982	0.000439	6.31E-05	8.24E-05	8.85E-05	2.23E-05
CGI_10000706	0.005935	NA	NA	NA	NA	2802	11	6.25E-05	9.06E-05	0.002793	0.006825	0.004094	0.001171	0.000482	0.000411	0.000308	0.00039	0.000134
CGI_10000270	0.001445	NA	NA	NA	NA	195	11	0.010106	0.03864	0.020978	0.006923	0.001999	0.001121	0.000371	3.60E-05	4.93E-05	1.85E-05	0
CGI_10021631	0.005806	NA	NA	NA	NA	1068	11	5.08E-06	0	0.000306	0.007853	0.004261	0.005759	0.009785	0.007308	0.004088	0.000808	4.08E-05
CGI_10013422	0.015659	NA	NA	NA	NA	306	11	0.000124	0.00014	0.000553	0.008174	0.013873	0.008937	0.001219	0.000604	0.00084	0.000926	0.000558
CGI_10008846	0.007517	NA	NA	NA	NA	225	11	5.43E-05	1.47E-05	0.003718	0.009207	0.006062	0.002503	0.001773	0.000509	0.00032	0.000233	2.42E-05
CGI_10017441	0.011206	NA	NA	NA	NA	327	11	0	8.07E-05	5.44E-05	0.009371	0.03105	0.027119	0.000937	1.07E-05	0	0	0
CGI_10007670	0.007718	NA	NA	NA	NA	840	11	1.13E-05	0.000679	0.000247	0.009392	0.01014	0.003015	0.003156	0.000515	0.001181	0.002714	0.005493
CGI_10024592	0.007626	NA	NA	NA	NA	924	11	1.47E-06	1.78E-06	0.000299	0.009949	0.018516	0.011787	0.004534	0.002278	0.002036	0.001031	6.49E-05
CGI_10016774	0.012363	NA	NA	NA	NA	570	11	0	4.05E-05	0.004554	0.010711	0.001972	0.000157	0	4.10E-06	0	9.50E-06	3.19E-06
CGI_10021950	0.006711	NA	NA	NA	NA	336	11	0	0	0.000115	0.014541	0.012033	0.001059	0.000783	0.000435	0.001402	0.001155	7.57E-05
CGI_10018236	NA	NA	NA	NA	NA	156	11	8.70E-05	0.000127	0.004298	0.015046	0.029866	0.025414	0.036211	0.009102	0.015557	0.003147	0.00986
CGI_10012511	0.007765	NA	NA	NA	NA	363	11	0	1.82E-05	0.000605	0.015654	0.015241	0.008004	0.000398	0	6.62E-06	0	0
CGI_10015606	0.001833	NA	NA	NA	NA	615	11	0.014013	0.02526	0.023963	0.020214	0.014303	0.012934	0.010191	0.005352	0.009889	0.007719	0.004409
CGI_10022617	0.006923	NA	NA	NA	NA	285	11	0	5.78E-06	0.001447	0.030804	0.003333	0.000501	0.00071	0.000213	0.000287	4.43E-05	1.27E-05
CGI_10016224	0.004385	NA	NA	NA	NA	450	11	9.05E-06	3.66E-06	0.000422	0.033164	0.073658	0.084237	0.034152	0.004091	0.002275	0.009149	0.000601
CGI_10005282	0.004176	NA	NA	NA	NA	540	11	7.54E-06	9.16E-06	0.010707	0.049664	0.05702	0.072999	0.038841	0.020431	0.01597	0.018334	0.002815
CGI_10012510	0.001879	NA	NA	NA	NA	150	11	0	0	0.001384	0.053654	0.062655	0.031474	0.000347	0	0	0	2.42E-05
CGI_10001095	0.003693	NA	NA	NA	NA	687	11	5.93E-06	4.80E-06	0.002958	0.067566	0.027816	0.00217	0.000644	6.81E-06	1.75E-05	1.58E-05	2.64E-06
CGI_10013406	0.005045	NA	NA	NA	NA	894	11	3.04E-06	2.77E-05	0.024797	0.113303	0.043183	0.003087	7.76E-05	1.31E-05	0	0	6.09E-06
CGI_10007242	0.002936	NA	NA	NA	NA	384	11	0	1.29E-05	0.020626	0.118837	0.062587	0.011434	0.0032	0.001715	0.001283	0.001598	0.000459

CGI_10013407	0.004207	NA	NA	NA	NA	402	11	0	0	0.018559	0.137533	0.090287	0.002154	7.19E-06	0	0	0	0
CGI_10025784	0.005079	NA	NA	NA	NA	444	11	8.56E-05	0.000111	0.022923	0.14731	0.11825	0.021134	0.0014	0.000271	5.95E-05	0	4.09E-06
CGI_10014301	0.00375	NA	NA	NA	NA	1353	11	1.00E-06	2.44E-06	0.004635	0.216347	0.201727	0.012226	0.000389	0.00011	9.06E-05	9.47E-05	8.05E-06
CGI_10025785	0.006236	NA	NA	NA	NA	678	11	0.000142	0.000182	0.045993	0.284311	0.238242	0.040987	0.003757	0.000668	0.000131	0	5.36E-06
CGI_10002165	0.003111	NA	NA	NA	NA	453	10	1.63E-05	6.62E-06	0.001298	0.039895	0.035152	0.026444	0.01368	0.016723	0.010213	0.012725	0.001436
CGI_10016777	0.004497	NA	NA	NA	NA	1191	10	6.22E-06	1.01E-05	0.017064	0.066109	0.063081	0.010747	6.18E-05	4.46E-06	0	2.76E-06	2.77E-06
CGI_10002164	0.003153	NA	NA	NA	NA	447	10	0	1.68E-05	0.010461	0.116134	0.096815	0.007915	0.000958	3.09E-05	0.000137	0.000529	0.000524
CGI_10025032	0.003226	NA	NA	NA	NA	699	10	0	0	0.025756	0.129253	0.124425	0.017925	0.000274	4.56E-06	3.44E-05	1.88E-05	2.83E-05
CGI_10025031	0.001773	NA	NA	NA	NA	318	10	0	5.65E-05	0.048152	0.196869	0.215289	0.06581	0.001678	2.67E-05	6.87E-06	0	0

Table S2 | **Summary of all the sampled developmental stages of Pacific oyster from the first replicate**. Stages with an asterisk in the column "Typical" are those chosen as typical development stages for the data analysis in this study.

**Stages dividing strategy**	**Figure label**	**Developmental age**	**Seawater temperature (℃)**	**Developmental stage**	**Typical**
Eggs	E	0 h 00 min	26	Egg	
Embryogenesis	TC	1 h 20 min	26	Two cells	
	FC	1 h 32 min	26	Four cells	
	SC	2 h 25 min	26	Sixteen cells	*
	EB	3 h 30 min	26	Early blastula	
	LB	4 h 35 min	26	Late blastula	*
	G	5 h 30 min	26	Gastrula	
	PT1	6 h 35 min	26	Pre-trochophore	
	PT2	7 h 35 min	26	Pre-trochophore	
	ET	8 h 30 min	26	Early trochophore	*
Trochophore	T1	9 h 30 min	26	Trochophore	
	T2	11 h 35 min	26	Trochophore	*
	T3	12 h 35 min	26	Trochophore	
	T4	13 h 35 min	26	Trochophore	
	T5	14 h 35 min	26	Trochophore	*
D-shape larvae	ED1	15 h 30 min	26	Early D-shape larva	
	ED2	16 h 35 min	26	Early D-shape larva	
	D1	17 h 35 min	26	D-shape larva	*
	D2	18 h 35 min	26	D-shape larva	
	D3	19 h 35 min	26	D-shape larva	
	D4	20 h 35 min	26	D-shape larva	
	D5	1.09 d	26	D-shape larva	*
	D6	1.75 d	26	D-shape larva	
	D7	3.77 d	26	D-shape larva	
Umbo larvae	EU1	4.77 d	25	Early umbo larva	
	EU2	6.75 d	25	Early umbo larva	
	U1	7.75 d	25	Umbo larva	*
	U2	8.75 d	25	Umbo larva	
	U3	9.75 d	25	Umbo larva	
	U4	10.77 d	25	Umbo larva	
	U5	12.76 d	25	Umbo larva	
	U6	13.75 d	25	Umbo larva	*
	LU1	14.73 d	25	Later umbo larva	
	LU2	15.73 d	25	Later umbo larva	
Pediveliger	P1	18.03 d	25	Pediveliger	
	P2	18.19 d	25	Pediveliger	*
Spat	S	22.15 d	25	Spat	*
Juvenile	J	215 d	Ambient, sea	Juvenile	

Table S3 | **Summary of all the sampled developmental stages of Pacific oyster from the second replicate**. Stages with an asterisk in the column "Typical" are those chosen as typical development stages for the data analysis in this study.

**Stages dividing strategy**	**Figure label**	**Developmental age**	**Seawater temperature (℃)**	**Developmental stage**	**Typical**
Embryogenesis	SC.2	2 h 25 min	22	Sixteen cells	*
	LB.2	4 h 35 min	22	Late blastula	*
	ET.2	8 h 30 min	22	Early trochophore	*
Trochophore	T1.2	12 h 35 min	22	Trochophore	*
	T2.2	14 h 35 min	22	Trochophore	*
D-shape larvae	D1.2	1.09 d	22	D-shape larva	*
	D2.2	1.75 d	22		
	D3.2	3.77 d	22		*
Umbo larvae	EU.2	7.75 d	22		
	U1.2	11.75 d	22	Umbo larva	*
	U2.2	16.75 d	22		
	U3.2	26 d	22	Umbo larva	*
	LU.2	28 d	22		
Pediveliger	P1.2	34 d	22	Pediveliger	*
	P2.2	37 d	22		
Spat	S	44 d	22	Spat	*

Table S4 | **Summary of developmental stages of Pacific abalone**.

**Stages dividing strategy**	**Figure label**	**Developmental age**	**Seawater temperature (℃)**	**Developmental stage**
Embryogenesis	EC	2 h	21.5	Eight cells
	SB	3 h 15 min	21.5	Stereoblastula
	G	6 h	21.5	Gastrula
Trochophore	T1	7 h 30 min	22	Trochophore
	T2	10 h 30 min	22	Trochophore
	T3	13 h 30 min	22	Trochophore
Veliger	V	43 h	22	Veliger
Groveling larvae	P	79 h	22	Pediveliger
Juvenile	J	1 year	Ambient	Juvenile

Table S5 | **Summary of developmental stages of sand worm**.

**Stages dividing strategy**	**Figure label**	**Developmental age**	**Seawater temperature (℃)**	**Developmental stage**
	Pb	2:12	22-25	1st polar body
Embryogenesis	SB	8:07	25	Stereoblastula
	PT	14:07	25	Protrochophore
Trochophore	ET	1 d 2:17	25	Early trochophore
	T	1 d 6:47	25	Trochophore
	MT	1 d 11:57	25	Metatrochophore
Juvenile	J5	9 d	25	Juvenile with 5-6 setigers
	J36	37 d	25	Juvenile with 36-47 setigers

Table S6 | **The reads hit number data of oyster, abalone and sand worm**.
See the excel file.

Table S7 | **The value of theta per nucleotide site for each oyster gene calculated with DnaSP software**.
See the excel file.

Table S8 | **The 3,915 genomes used in the phylostratigraphic analysis**.

Phylostrata	Name	Species
1	Cellular_org	'Nostoc azollae' 0708
1	Cellular_org	'Rehmannia glutinosa' phytoplasma
1	Cellular_org	Abiotrophia defectiva
1	Cellular_org	Abiotrophia defectiva ATCC 49176
1	Cellular_org	Acaryochloris marina
1	Cellular_org	Acaryochloris marina MBIC11017
1	Cellular_org	Acetivibrio cellulolyticus
1	Cellular_org	Acetivibrio cellulolyticus CD2
1	Cellular_org	Acetobacter aceti
1	Cellular_org	Acetobacter pasteurianus
1	Cellular_org	Acetobacter pasteurianus IFO 3283-01
1	Cellular_org	Acetohalobium arabaticum DSM 5501
1	Cellular_org	Acholeplasma laidlawii
1	Cellular_org	Acholeplasma laidlawii PG-8A
1	Cellular_org	Achromobacter denitrificans
1	Cellular_org	Achromobacter piechaudii
1	Cellular_org	Achromobacter piechaudii ATCC 43553
1	Cellular_org	Achromobacter xylosoxidans
1	Cellular_org	Achromobacter xylosoxidans A8
1	Cellular_org	Acidaminococcus fermentans
1	Cellular_org	Acidaminococcus fermentans DSM 20731
1	Cellular_org	Acidaminococcus sp. D21
1	Cellular_org	Acidimicrobium ferrooxidans DSM 10331
1	Cellular_org	Acidiphilium cryptum
1	Cellular_org	Acidiphilium cryptum JF-5
1	Cellular_org	Acidiphilium multivorum
1	Cellular_org	Acidithiobacillus caldus
1	Cellular_org	Acidithiobacillus caldus ATCC 51756
1	Cellular_org	Acidithiobacillus ferrooxidans
1	Cellular_org	Acidithiobacillus ferrooxidans ATCC 23270
1	Cellular_org	Acidithiobacillus ferrooxidans ATCC 53993
1	Cellular_org	Acidobacterium capsulatum
1	Cellular_org	Acidobacterium capsulatum ATCC 51196
1	Cellular_org	Acidobacterium sp. MP5ACTX8
1	Cellular_org	Acidobacterium sp. MP5ACTX9
1	Cellular_org	Acidothermus cellulolyticus
1	Cellular_org	Acidothermus cellulolyticus 11B
1	Cellular_org	Acidovorax citrulli
1	Cellular_org	Acidovorax citrulli AAC00-1
1	Cellular_org	Acidovorax delafieldii
1	Cellular_org	Acidovorax delafieldii 2AN

1	Cellular_org	Acidovorax ebreus TPSY
1	Cellular_org	Acidovorax sp. JS42
1	Cellular_org	Acinetobacter baumannii
1	Cellular_org	Acinetobacter baumannii AB0057
1	Cellular_org	Acinetobacter baumannii AB056
1	Cellular_org	Acinetobacter baumannii AB058
1	Cellular_org	Acinetobacter baumannii AB059
1	Cellular_org	Acinetobacter baumannii AB307-0294
1	Cellular_org	Acinetobacter baumannii AB900
1	Cellular_org	Acinetobacter baumannii ACICU
1	Cellular_org	Acinetobacter baumannii ATCC 17978
1	Cellular_org	Acinetobacter baumannii ATCC 19606
1	Cellular_org	Acinetobacter baumannii AYE
1	Cellular_org	Acinetobacter baumannii SDF
1	Cellular_org	Acinetobacter calcoaceticus
1	Cellular_org	Acinetobacter calcoaceticus RUH2202
1	Cellular_org	Acinetobacter genomosp. 13TU
1	Cellular_org	Acinetobacter genomosp. 3
1	Cellular_org	Acinetobacter haemolyticus
1	Cellular_org	Acinetobacter haemolyticus ATCC 19194
1	Cellular_org	Acinetobacter johnsonii
1	Cellular_org	Acinetobacter johnsonii SH046
1	Cellular_org	Acinetobacter junii
1	Cellular_org	Acinetobacter junii SH205
1	Cellular_org	Acinetobacter lwoffii
1	Cellular_org	Acinetobacter lwoffii SH145
1	Cellular_org	Acinetobacter radioresistens
1	Cellular_org	Acinetobacter radioresistens SH164
1	Cellular_org	Acinetobacter radioresistens SK82
1	Cellular_org	Acinetobacter sp. ADP1
1	Cellular_org	Acinetobacter sp. ATCC 27244
1	Cellular_org	Acinetobacter sp. DR1
1	Cellular_org	Acinetobacter sp. EB104
1	Cellular_org	Acinetobacter sp. RUH2624
1	Cellular_org	Acinetobacter sp. SH024
1	Cellular_org	Acinetobacter sp. SUN
1	Cellular_org	Acinetobacter venetianus
1	Cellular_org	Actinobacillus minor
1	Cellular_org	Actinobacillus minor 202
1	Cellular_org	Actinobacillus minor NM305
1	Cellular_org	Actinobacillus pleuropneumoniae
1	Cellular_org	Actinobacillus pleuropneumoniae serovar 1
1	Cellular_org	Actinobacillus pleuropneumoniae serovar 1 str. 4074

1	Cellular_org	Actinobacillus pleuropneumoniae serovar 10 str. D13039
1	Cellular_org	Actinobacillus pleuropneumoniae serovar 11 str. 56153
1	Cellular_org	Actinobacillus pleuropneumoniae serovar 12 str. 1096
1	Cellular_org	Actinobacillus pleuropneumoniae serovar 13 str. N273
1	Cellular_org	Actinobacillus pleuropneumoniae serovar 2
1	Cellular_org	Actinobacillus pleuropneumoniae serovar 2 str. 4226
1	Cellular_org	Actinobacillus pleuropneumoniae serovar 2 str. S1536
1	Cellular_org	Actinobacillus pleuropneumoniae serovar 3 str. JL03
1	Cellular_org	Actinobacillus pleuropneumoniae serovar 4 str. M62
1	Cellular_org	Actinobacillus pleuropneumoniae serovar 5
1	Cellular_org	Actinobacillus pleuropneumoniae serovar 6 str. Femo
1	Cellular_org	Actinobacillus pleuropneumoniae serovar 7 str. AP76
1	Cellular_org	Actinobacillus pleuropneumoniae serovar 9 str. CVJ13261
1	Cellular_org	Actinobacillus porcitonsillarum
1	Cellular_org	Actinobacillus succinogenes
1	Cellular_org	Actinobacillus succinogenes 130Z
1	Cellular_org	Actinobacillus ureae
1	Cellular_org	Actinomyces coleocanis DSM 15436
1	Cellular_org	Actinomyces odontolyticus
1	Cellular_org	Actinomyces odontolyticus ATCC 17982
1	Cellular_org	Actinomyces odontolyticus F0309
1	Cellular_org	Actinomyces sp. oral taxon 848 str. F0332
1	Cellular_org	Actinomyces urogenitalis DSM 15434
1	Cellular_org	Actinomyces viscosus
1	Cellular_org	Actinomyces viscosus C505
1	Cellular_org	Actinosynnema mirum
1	Cellular_org	Actinosynnema mirum DSM 43827
1	Cellular_org	Aerococcus viridans
1	Cellular_org	Aerococcus viridans ATCC 11563
1	Cellular_org	Aeromicrobium marinum DSM 15272
1	Cellular_org	Aeromonas bestiarum
1	Cellular_org	Aeromonas caviae
1	Cellular_org	Aeromonas hydrophila
1	Cellular_org	Aeromonas hydrophila subsp. hydrophila
1	Cellular_org	Aeromonas hydrophila subsp. hydrophila ATCC 7966
1	Cellular_org	Aeromonas salmonicida
1	Cellular_org	Aeromonas salmonicida subsp. salmonicida
1	Cellular_org	Aeromonas salmonicida subsp. salmonicida A449
1	Cellular_org	Afipia sp. 1NLS2
1	Cellular_org	Aggregatibacter actinomycetemcomitans
1	Cellular_org	Aggregatibacter actinomycetemcomitans D11S-1
1	Cellular_org	Aggregatibacter actinomycetemcomitans D7S-1
1	Cellular_org	Aggregatibacter aphrophilus

1	Cellular_org	Aggregatibacter aphrophilus NJ8700
1	Cellular_org	Aggregatibacter segnis
1	Cellular_org	Agrobacterium radiobacter K84
1	Cellular_org	Agrobacterium rhizogenes
1	Cellular_org	Agrobacterium tumefaciens
1	Cellular_org	Agrobacterium tumefaciens str. C58
1	Cellular_org	Agrobacterium vitis
1	Cellular_org	Agrobacterium vitis S4
1	Cellular_org	Ahrensia sp. R2A130
1	Cellular_org	Akkermansia muciniphila
1	Cellular_org	Akkermansia muciniphila ATCC BAA-835
1	Cellular_org	Alcanivorax borkumensis
1	Cellular_org	Alcanivorax borkumensis SK2
1	Cellular_org	Alcanivorax sp. DG881
1	Cellular_org	Algoriphagus sp. PR1
1	Cellular_org	Alicycliphilus denitrificans BC
1	Cellular_org	Alicyclobacillus acidocaldarius
1	Cellular_org	Alicyclobacillus acidocaldarius LAA1
1	Cellular_org	Alicyclobacillus acidocaldarius subsp. acidocaldarius
1	Cellular_org	Alicyclobacillus acidocaldarius subsp. acidocaldarius DSM 446
1	Cellular_org	Aliivibrio fischeri
1	Cellular_org	Aliivibrio salmonicida
1	Cellular_org	Aliivibrio salmonicida LFI1238
1	Cellular_org	Alistipes putredinis
1	Cellular_org	Alistipes putredinis DSM 17216
1	Cellular_org	Alkalilimnicola ehrlichii MLHE-1
1	Cellular_org	Alkaliphilus metalliredigens QYMF
1	Cellular_org	Alkaliphilus oremlandii OhILAs
1	Cellular_org	Allochromatium vinosum
1	Cellular_org	Allochromatium vinosum DSM 180
1	Cellular_org	Alteromonadales bacterium TW-7
1	Cellular_org	Alteromonas
1	Cellular_org	Alteromonas macleodii
1	Cellular_org	Alteromonas macleodii 'Deep ecotype'
1	Cellular_org	Alteromonas macleodii ATCC 27126
1	Cellular_org	Aminobacterium colombiense DSM 12261
1	Cellular_org	Aminomonas paucivorans DSM 12260
1	Cellular_org	Ammonifex degensii KC4
1	Cellular_org	Amycolatopsis benzoatilytica
1	Cellular_org	Amycolatopsis mediterranei
1	Cellular_org	Amycolatopsis mediterranei U32
1	Cellular_org	Anabaena

1	Cellular_org	Anabaena azollae
1	Cellular_org	Anabaena variabilis
1	Cellular_org	Anabaena variabilis ATCC 29413
1	Cellular_org	Anaerobaculum hydrogeniformans ATCC BAA-1850
1	Cellular_org	Anaerococcus hydrogenalis DSM 7454
1	Cellular_org	Anaerococcus lactolyticus
1	Cellular_org	Anaerococcus lactolyticus ATCC 51172
1	Cellular_org	Anaerococcus prevotii
1	Cellular_org	Anaerococcus prevotii DSM 20548
1	Cellular_org	Anaerococcus tetradius ATCC 35098
1	Cellular_org	Anaerococcus vaginalis ATCC 51170
1	Cellular_org	Anaerofustis stercorihominis DSM 17244
1	Cellular_org	Anaeromyxobacter dehalogenans
1	Cellular_org	Anaeromyxobacter dehalogenans 2CP-1
1	Cellular_org	Anaeromyxobacter dehalogenans 2CP-C
1	Cellular_org	Anaeromyxobacter sp. Fw109-5
1	Cellular_org	Anaeromyxobacter sp. K
1	Cellular_org	Anaerostipes caccae
1	Cellular_org	Anaerostipes caccae DSM 14662
1	Cellular_org	Anaerotruncus colihominis DSM 17241
1	Cellular_org	Anaplasma centrale
1	Cellular_org	Anaplasma centrale str. Israel
1	Cellular_org	Anaplasma marginale
1	Cellular_org	Anaplasma marginale str. Florida
1	Cellular_org	Anaplasma marginale str. Mississippi
1	Cellular_org	Anaplasma marginale str. Puerto Rico
1	Cellular_org	Anaplasma marginale str. St. Maries
1	Cellular_org	Anaplasma marginale str. Virginia
1	Cellular_org	Anaplasma phagocytophilum HZ
1	Cellular_org	Anoxybacillus flavithermus
1	Cellular_org	Anoxybacillus flavithermus WK1
1	Cellular_org	Aquifex aeolicus
1	Cellular_org	Aquifex aeolicus VF5
1	Cellular_org	Arcanobacterium haemolyticum
1	Cellular_org	Arcanobacterium haemolyticum DSM 20595
1	Cellular_org	Arcanobacterium pyogenes
1	Cellular_org	Arcobacter butzleri
1	Cellular_org	Arcobacter butzleri RM4018
1	Cellular_org	Arcobacter nitrofigilis
1	Cellular_org	Arcobacter nitrofigilis DSM 7299
1	Cellular_org	Aromatoleum aromaticum EbN1
1	Cellular_org	Arthrobacter arilaitensis
1	Cellular_org	Arthrobacter arilaitensis Re117

1	Cellular_org	Arthrobacter aurescens
1	Cellular_org	Arthrobacter aurescens TC1
1	Cellular_org	Arthrobacter chlorophenolicus
1	Cellular_org	Arthrobacter chlorophenolicus A6
1	Cellular_org	Arthrobacter nitroguajacolicus
1	Cellular_org	Arthrobacter sp. AK-1
1	Cellular_org	Arthrobacter sp. Chr15
1	Cellular_org	Arthrobacter sp. FB24
1	Cellular_org	Arthrospira maxima
1	Cellular_org	Arthrospira maxima CS-328
1	Cellular_org	Arthrospira platensis
1	Cellular_org	Arthrospira platensis str. Paraca
1	Cellular_org	Aster yellows phytoplasma
1	Cellular_org	Aster yellows witches'-broom phytoplasma
1	Cellular_org	Aster yellows witches'-broom phytoplasma AYWB
1	Cellular_org	Asticcacaulis excentricus CB 48
1	Cellular_org	Atopobium parvulum DSM 20469
1	Cellular_org	Atopobium rimae ATCC 49626
1	Cellular_org	Atopobium vaginae
1	Cellular_org	Atopobium vaginae DSM 15829
1	Cellular_org	Atopobium vaginae PB189-T1-4
1	Cellular_org	Aurantimonas manganoxydans SI85-9A1
1	Cellular_org	Avibacterium paragallinarum
1	Cellular_org	Azoarcus sp. BH72
1	Cellular_org	Azorhizobium caulinodans
1	Cellular_org	Azorhizobium caulinodans ORS 571
1	Cellular_org	Azospirillum sp. B510
1	Cellular_org	Azotobacter vinelandii
1	Cellular_org	Azotobacter vinelandii DJ
1	Cellular_org	Bacillus amyloliquefaciens
1	Cellular_org	Bacillus amyloliquefaciens FZB42
1	Cellular_org	Bacillus anthracis Tsiankovskii-I
1	Cellular_org	Bacillus anthracis str. 'Ames Ancestor'
1	Cellular_org	Bacillus anthracis str. A0174
1	Cellular_org	Bacillus anthracis str. A0193
1	Cellular_org	Bacillus anthracis str. A0248
1	Cellular_org	Bacillus anthracis str. A0389
1	Cellular_org	Bacillus anthracis str. A0442
1	Cellular_org	Bacillus anthracis str. A0465
1	Cellular_org	Bacillus anthracis str. A0488
1	Cellular_org	Bacillus anthracis str. A1055
1	Cellular_org	Bacillus anthracis str. A2012
1	Cellular_org	Bacillus anthracis str. Ames

1	Cellular_org	Bacillus anthracis str. Australia 94
1	Cellular_org	Bacillus anthracis str. CDC 684
1	Cellular_org	Bacillus anthracis str. CNEVA-9066
1	Cellular_org	Bacillus anthracis str. Kruger B
1	Cellular_org	Bacillus anthracis str. Sterne
1	Cellular_org	Bacillus anthracis str. Vollum
1	Cellular_org	Bacillus anthracis str. Western North America USA6153
1	Cellular_org	Bacillus atrophaeus
1	Cellular_org	Bacillus cellulosilyticus DSM 2522
1	Cellular_org	Bacillus cereus
1	Cellular_org	Bacillus cereus 03BB102
1	Cellular_org	Bacillus cereus 03BB108
1	Cellular_org	Bacillus cereus 172560W
1	Cellular_org	Bacillus cereus 95/8201
1	Cellular_org	Bacillus cereus AH1134
1	Cellular_org	Bacillus cereus AH1271
1	Cellular_org	Bacillus cereus AH1272
1	Cellular_org	Bacillus cereus AH1273
1	Cellular_org	Bacillus cereus AH187
1	Cellular_org	Bacillus cereus AH603
1	Cellular_org	Bacillus cereus AH621
1	Cellular_org	Bacillus cereus AH676
1	Cellular_org	Bacillus cereus AH820
1	Cellular_org	Bacillus cereus ATCC 10876
1	Cellular_org	Bacillus cereus ATCC 10987
1	Cellular_org	Bacillus cereus ATCC 14579
1	Cellular_org	Bacillus cereus ATCC 4342
1	Cellular_org	Bacillus cereus B4264
1	Cellular_org	Bacillus cereus BDRD-Cer4
1	Cellular_org	Bacillus cereus BDRD-ST196
1	Cellular_org	Bacillus cereus BDRD-ST24
1	Cellular_org	Bacillus cereus BDRD-ST26
1	Cellular_org	Bacillus cereus BGSC 6E1
1	Cellular_org	Bacillus cereus E33L
1	Cellular_org	Bacillus cereus F65185
1	Cellular_org	Bacillus cereus G9241
1	Cellular_org	Bacillus cereus G9842
1	Cellular_org	Bacillus cereus H3081.97
1	Cellular_org	Bacillus cereus MM3
1	Cellular_org	Bacillus cereus NVH0597-99
1	Cellular_org	Bacillus cereus Q1
1	Cellular_org	Bacillus cereus R309803
1	Cellular_org	Bacillus cereus Rock1-15

1	Cellular_org	Bacillus cereus Rock1-3
1	Cellular_org	Bacillus cereus Rock3-28
1	Cellular_org	Bacillus cereus Rock3-29
1	Cellular_org	Bacillus cereus Rock3-42
1	Cellular_org	Bacillus cereus Rock3-44
1	Cellular_org	Bacillus cereus Rock4-18
1	Cellular_org	Bacillus cereus Rock4-2
1	Cellular_org	Bacillus cereus SJ1
1	Cellular_org	Bacillus cereus W
1	Cellular_org	Bacillus cereus biovar anthracis str. CI
1	Cellular_org	Bacillus cereus m1293
1	Cellular_org	Bacillus cereus m1550
1	Cellular_org	Bacillus clausii
1	Cellular_org	Bacillus clausii KSM-K16
1	Cellular_org	Bacillus coagulans
1	Cellular_org	Bacillus coagulans 36D1
1	Cellular_org	Bacillus coahuilensis
1	Cellular_org	Bacillus coahuilensis m4-4
1	Cellular_org	Bacillus cytotoxicus
1	Cellular_org	Bacillus cytotoxicus NVH 391-98
1	Cellular_org	Bacillus halodurans
1	Cellular_org	Bacillus halodurans C-125
1	Cellular_org	Bacillus licheniformis
1	Cellular_org	Bacillus licheniformis ATCC 14580
1	Cellular_org	Bacillus megaterium
1	Cellular_org	Bacillus megaterium DSM 319
1	Cellular_org	Bacillus megaterium QM B1551
1	Cellular_org	Bacillus methanolicus
1	Cellular_org	Bacillus methanolicus MGA3
1	Cellular_org	Bacillus mycoides
1	Cellular_org	Bacillus mycoides DSM 2048
1	Cellular_org	Bacillus mycoides Rock1-4
1	Cellular_org	Bacillus mycoides Rock3-17
1	Cellular_org	Bacillus pseudofirmus
1	Cellular_org	Bacillus pseudofirmus OF4
1	Cellular_org	Bacillus pseudomycoides
1	Cellular_org	Bacillus pseudomycoides DSM 12442
1	Cellular_org	Bacillus pumilus
1	Cellular_org	Bacillus pumilus ATCC 7061
1	Cellular_org	Bacillus pumilus SAFR-032
1	Cellular_org	Bacillus selenitireducens MLS10
1	Cellular_org	Bacillus sp. B-3
1	Cellular_org	Bacillus sp. B14905

1	Cellular_org	Bacillus sp. BS-01
1	Cellular_org	Bacillus sp. BS-02
1	Cellular_org	Bacillus sp. JAMB750
1	Cellular_org	Bacillus sp. NRRL B-14911
1	Cellular_org	Bacillus sp. SG-1
1	Cellular_org	Bacillus subtilis
1	Cellular_org	Bacillus subtilis subsp. natto
1	Cellular_org	Bacillus subtilis subsp. spizizenii
1	Cellular_org	Bacillus subtilis subsp. spizizenii ATCC 6633
1	Cellular_org	Bacillus subtilis subsp. spizizenii str. W23
1	Cellular_org	Bacillus subtilis subsp. subtilis
1	Cellular_org	Bacillus subtilis subsp. subtilis str. 168
1	Cellular_org	Bacillus subtilis subsp. subtilis str. JH642
1	Cellular_org	Bacillus subtilis subsp. subtilis str. NCIB 3610
1	Cellular_org	Bacillus subtilis subsp. subtilis str. SMY
1	Cellular_org	Bacillus thuringiensis
1	Cellular_org	Bacillus thuringiensis BMB171
1	Cellular_org	Bacillus thuringiensis Bt407
1	Cellular_org	Bacillus thuringiensis IBL 200
1	Cellular_org	Bacillus thuringiensis IBL 4222
1	Cellular_org	Bacillus thuringiensis serovar andalousiensis
1	Cellular_org	Bacillus thuringiensis serovar andalousiensis BGSC 4AW1
1	Cellular_org	Bacillus thuringiensis serovar berliner
1	Cellular_org	Bacillus thuringiensis serovar berliner ATCC 10792
1	Cellular_org	Bacillus thuringiensis serovar darmstadiensis
1	Cellular_org	Bacillus thuringiensis serovar entomocidus
1	Cellular_org	Bacillus thuringiensis serovar huazhongensis
1	Cellular_org	Bacillus thuringiensis serovar huazhongensis BGSC 4BD1
1	Cellular_org	Bacillus thuringiensis serovar israelensis
1	Cellular_org	Bacillus thuringiensis serovar israelensis ATCC 35646
1	Cellular_org	Bacillus thuringiensis serovar konkukian
1	Cellular_org	Bacillus thuringiensis serovar konkukian str. 97-27
1	Cellular_org	Bacillus thuringiensis serovar kurstaki
1	Cellular_org	Bacillus thuringiensis serovar kurstaki str. T03a001
1	Cellular_org	Bacillus thuringiensis serovar kurstaki str. YBT-1520
1	Cellular_org	Bacillus thuringiensis serovar monterrey
1	Cellular_org	Bacillus thuringiensis serovar monterrey BGSC 4AJ1
1	Cellular_org	Bacillus thuringiensis serovar pakistani
1	Cellular_org	Bacillus thuringiensis serovar pakistani str. T13001
1	Cellular_org	Bacillus thuringiensis serovar pondicheriensis
1	Cellular_org	Bacillus thuringiensis serovar pondicheriensis BGSC 4BA1
1	Cellular_org	Bacillus thuringiensis serovar pulsiensis
1	Cellular_org	Bacillus thuringiensis serovar pulsiensis BGSC 4CC1

1	Cellular_org	Bacillus thuringiensis serovar sotto
1	Cellular_org	Bacillus thuringiensis serovar sotto str. T04001
1	Cellular_org	Bacillus thuringiensis serovar tenebrionis
1	Cellular_org	Bacillus thuringiensis serovar thuringiensis
1	Cellular_org	Bacillus thuringiensis serovar thuringiensis str. T01001
1	Cellular_org	Bacillus thuringiensis serovar tochigiensis
1	Cellular_org	Bacillus thuringiensis serovar tochigiensis BGSC 4Y1
1	Cellular_org	Bacillus thuringiensis str. Al Hakam
1	Cellular_org	Bacillus tusciae
1	Cellular_org	Bacillus tusciae DSM 2912
1	Cellular_org	Bacillus weihenstephanensis
1	Cellular_org	Bacillus weihenstephanensis KBAB4
1	Cellular_org	Bacteroides caccae
1	Cellular_org	Bacteroides caccae ATCC 43185
1	Cellular_org	Bacteroides capillosus ATCC 29799
1	Cellular_org	Bacteroides cellulosilyticus
1	Cellular_org	Bacteroides cellulosilyticus DSM 14838
1	Cellular_org	Bacteroides coprocola
1	Cellular_org	Bacteroides coprocola DSM 17136
1	Cellular_org	Bacteroides coprophilus
1	Cellular_org	Bacteroides coprophilus DSM 18228
1	Cellular_org	Bacteroides dorei 5_1_36/D4
1	Cellular_org	Bacteroides dorei DSM 17855
1	Cellular_org	Bacteroides eggerthii
1	Cellular_org	Bacteroides eggerthii DSM 20697
1	Cellular_org	Bacteroides finegoldii DSM 17565
1	Cellular_org	Bacteroides fragilis
1	Cellular_org	Bacteroides fragilis 3_1_12
1	Cellular_org	Bacteroides fragilis NCTC 9343
1	Cellular_org	Bacteroides fragilis YCH46
1	Cellular_org	Bacteroides helcogenes
1	Cellular_org	Bacteroides helcogenes P 36-108
1	Cellular_org	Bacteroides intestinalis DSM 17393
1	Cellular_org	Bacteroides ovatus
1	Cellular_org	Bacteroides ovatus ATCC 8483
1	Cellular_org	Bacteroides ovatus SD CC 2a
1	Cellular_org	Bacteroides ovatus SD CMC 3f
1	Cellular_org	Bacteroides pectinophilus ATCC 43243
1	Cellular_org	Bacteroides plebeius
1	Cellular_org	Bacteroides plebeius DSM 17135
1	Cellular_org	Bacteroides sp. 1_1_14
1	Cellular_org	Bacteroides sp. 1_1_6
1	Cellular_org	Bacteroides sp. 20_3

1	Cellular_org	Bacteroides sp. 2_1_16
1	Cellular_org	Bacteroides sp. 2_1_22
1	Cellular_org	Bacteroides sp. 2_1_33B
1	Cellular_org	Bacteroides sp. 2_1_7
1	Cellular_org	Bacteroides sp. 2_2_4
1	Cellular_org	Bacteroides sp. 3_1_19
1	Cellular_org	Bacteroides sp. 3_1_23
1	Cellular_org	Bacteroides sp. 3_1_33FAA
1	Cellular_org	Bacteroides sp. 3_2_5
1	Cellular_org	Bacteroides sp. 4_3_47FAA
1	Cellular_org	Bacteroides sp. 9_1_42FAA
1	Cellular_org	Bacteroides sp. D1
1	Cellular_org	Bacteroides sp. D2
1	Cellular_org	Bacteroides sp. D20
1	Cellular_org	Bacteroides sp. D22
1	Cellular_org	Bacteroides stercoris
1	Cellular_org	Bacteroides stercoris ATCC 43183
1	Cellular_org	Bacteroides thetaiotaomicron
1	Cellular_org	Bacteroides thetaiotaomicron VPI-5482
1	Cellular_org	Bacteroides uniformis
1	Cellular_org	Bacteroides uniformis ATCC 8492
1	Cellular_org	Bacteroides vulgatus
1	Cellular_org	Bacteroides vulgatus ATCC 8482
1	Cellular_org	Bacteroides vulgatus PC510
1	Cellular_org	Bacteroides xylanisolvens
1	Cellular_org	Bacteroides xylanisolvens SD CC 1b
1	Cellular_org	Bacteroidetes oral taxon 274 str. F0058
1	Cellular_org	Bartonella bacilliformis
1	Cellular_org	Bartonella bacilliformis KC583
1	Cellular_org	Bartonella clarridgeiae
1	Cellular_org	Bartonella grahamii
1	Cellular_org	Bartonella grahamii as4aup
1	Cellular_org	Bartonella henselae
1	Cellular_org	Bartonella henselae str. Houston-1
1	Cellular_org	Bartonella quintana
1	Cellular_org	Bartonella quintana str. Toulouse
1	Cellular_org	Bartonella tribocorum
1	Cellular_org	Bartonella tribocorum CIP 105476
1	Cellular_org	Baumannia cicadellinicola str. Hc (Homalodisca coagulata)
1	Cellular_org	Bdellovibrio bacteriovorus
1	Cellular_org	Bdellovibrio bacteriovorus HD100
1	Cellular_org	Beet leafhopper transmitted virescence phytoplasma
1	Cellular_org	Beggiatoa sp. PS

1	Cellular_org	Beggiatoa sp. SS
1	Cellular_org	Beijerinckia indica subsp. indica
1	Cellular_org	Beijerinckia indica subsp. indica ATCC 9039
1	Cellular_org	Beutenbergia cavernae DSM 12333
1	Cellular_org	Bibersteinia trehalosi
1	Cellular_org	Bifidobacterium adolescentis
1	Cellular_org	Bifidobacterium adolescentis ATCC 15703
1	Cellular_org	Bifidobacterium adolescentis L2-32
1	Cellular_org	Bifidobacterium angulatum
1	Cellular_org	Bifidobacterium angulatum DSM 20098
1	Cellular_org	Bifidobacterium animalis
1	Cellular_org	Bifidobacterium animalis subsp. lactis
1	Cellular_org	Bifidobacterium animalis subsp. lactis AD011
1	Cellular_org	Bifidobacterium animalis subsp. lactis Bl-04
1	Cellular_org	Bifidobacterium animalis subsp. lactis DSM 10140
1	Cellular_org	Bifidobacterium animalis subsp. lactis HN019
1	Cellular_org	Bifidobacterium asteroides
1	Cellular_org	Bifidobacterium bifidum
1	Cellular_org	Bifidobacterium bifidum NCIMB 41171
1	Cellular_org	Bifidobacterium bifidum S17
1	Cellular_org	Bifidobacterium breve
1	Cellular_org	Bifidobacterium breve DSM 20213
1	Cellular_org	Bifidobacterium catenulatum
1	Cellular_org	Bifidobacterium catenulatum DSM 16992
1	Cellular_org	Bifidobacterium dentium
1	Cellular_org	Bifidobacterium dentium ATCC 27678
1	Cellular_org	Bifidobacterium dentium ATCC 27679
1	Cellular_org	Bifidobacterium dentium Bd1
1	Cellular_org	Bifidobacterium dentium JCVIHMP022
1	Cellular_org	Bifidobacterium gallicum
1	Cellular_org	Bifidobacterium gallicum DSM 20093
1	Cellular_org	Bifidobacterium longum
1	Cellular_org	Bifidobacterium longum DJO10A
1	Cellular_org	Bifidobacterium longum NCC2705
1	Cellular_org	Bifidobacterium longum subsp. infantis
1	Cellular_org	Bifidobacterium longum subsp. infantis ATCC 15697
1	Cellular_org	Bifidobacterium longum subsp. infantis ATCC 55813
1	Cellular_org	Bifidobacterium longum subsp. infantis CCUG 52486
1	Cellular_org	Bifidobacterium longum subsp. longum
1	Cellular_org	Bifidobacterium longum subsp. longum JCM 1217
1	Cellular_org	Bifidobacterium longum subsp. longum JDM301
1	Cellular_org	Bifidobacterium pseudocatenulatum
1	Cellular_org	Bifidobacterium pseudocatenulatum DSM 20438

1	Cellular_org	Bifidobacterium pseudolongum
1	Cellular_org	Bifidobacterium pseudolongum subsp. globosum
1	Cellular_org	Bifidobacterium sp. A24
1	Cellular_org	Bilophila wadsworthia
1	Cellular_org	Blastopirellula marina DSM 3645
1	Cellular_org	Blattabacterium sp. (Blattella germanica) str. Bge
1	Cellular_org	Blattabacterium sp. (Periplaneta americana) str. BPLAN
1	Cellular_org	Blautia hansenii
1	Cellular_org	Blautia hansenii DSM 20583
1	Cellular_org	Blautia hydrogenotrophica DSM 10507
1	Cellular_org	Bordetella avium
1	Cellular_org	Bordetella avium 197N
1	Cellular_org	Bordetella bronchiseptica
1	Cellular_org	Bordetella bronchiseptica RB50
1	Cellular_org	Bordetella parapertussis
1	Cellular_org	Bordetella parapertussis 12822
1	Cellular_org	Bordetella pertussis
1	Cellular_org	Bordetella pertussis Tohama I
1	Cellular_org	Bordetella petrii
1	Cellular_org	Bordetella petrii DSM 12804
1	Cellular_org	Borrelia afzelii
1	Cellular_org	Borrelia afzelii ACA-1
1	Cellular_org	Borrelia afzelii PKo
1	Cellular_org	Borrelia bavariensis
1	Cellular_org	Borrelia burgdorferi 118a
1	Cellular_org	Borrelia burgdorferi 156a
1	Cellular_org	Borrelia burgdorferi 29805
1	Cellular_org	Borrelia burgdorferi 64b
1	Cellular_org	Borrelia burgdorferi 72a
1	Cellular_org	Borrelia burgdorferi 80a
1	Cellular_org	Borrelia burgdorferi 94a
1	Cellular_org	Borrelia burgdorferi B31
1	Cellular_org	Borrelia burgdorferi Bol26
1	Cellular_org	Borrelia burgdorferi WI91-23
1	Cellular_org	Borrelia burgdorferi ZS7
1	Cellular_org	Borrelia duttonii
1	Cellular_org	Borrelia duttonii Ly
1	Cellular_org	Borrelia garinii
1	Cellular_org	Borrelia garinii Far04
1	Cellular_org	Borrelia garinii PBi
1	Cellular_org	Borrelia garinii PBr
1	Cellular_org	Borrelia hermsii
1	Cellular_org	Borrelia hermsii DAH

1	Cellular_org	Borrelia recurrentis
1	Cellular_org	Borrelia recurrentis A1
1	Cellular_org	Borrelia sp. SV1
1	Cellular_org	Borrelia spielmanii
1	Cellular_org	Borrelia spielmanii A14S
1	Cellular_org	Borrelia turicatae
1	Cellular_org	Borrelia turicatae 91E135
1	Cellular_org	Borrelia valaisiana
1	Cellular_org	Borrelia valaisiana VS116
1	Cellular_org	Brachybacterium faecium DSM 4810
1	Cellular_org	Brachyspira hyodysenteriae
1	Cellular_org	Brachyspira hyodysenteriae WA1
1	Cellular_org	Brachyspira murdochii
1	Cellular_org	Brachyspira murdochii DSM 12563
1	Cellular_org	Brachyspira pilosicoli
1	Cellular_org	Brachyspira pilosicoli 95/1000
1	Cellular_org	Bradyrhizobium japonicum
1	Cellular_org	Bradyrhizobium japonicum USDA 110
1	Cellular_org	Bradyrhizobium sp. BTAi1
1	Cellular_org	Bradyrhizobium sp. ORS278
1	Cellular_org	Brevibacillus borstelensis
1	Cellular_org	Brevibacillus brevis
1	Cellular_org	Brevibacillus brevis NBRC 100599
1	Cellular_org	Brevibacterium
1	Cellular_org	Brevibacterium linens
1	Cellular_org	Brevibacterium linens BL2
1	Cellular_org	Brevibacterium mcbrellneri ATCC 49030
1	Cellular_org	Brevundimonas sp. BAL3
1	Cellular_org	Brevundimonas subvibrioides
1	Cellular_org	Brevundimonas subvibrioides ATCC 15264
1	Cellular_org	Brucella abortus
1	Cellular_org	Brucella abortus NCTC 8038
1	Cellular_org	Brucella abortus S19
1	Cellular_org	Brucella abortus bv. 1
1	Cellular_org	Brucella abortus bv. 1 str. 9-941
1	Cellular_org	Brucella abortus bv. 2 str. 86/8/59
1	Cellular_org	Brucella abortus bv. 3
1	Cellular_org	Brucella abortus bv. 3 str. Tulya
1	Cellular_org	Brucella abortus bv. 4 str. 292
1	Cellular_org	Brucella abortus bv. 5 str. B3196
1	Cellular_org	Brucella abortus bv. 6 str. 870
1	Cellular_org	Brucella abortus bv. 9 str. C68
1	Cellular_org	Brucella abortus str. 2308 A

1	Cellular_org	Brucella canis
1	Cellular_org	Brucella canis ATCC 23365
1	Cellular_org	Brucella ceti
1	Cellular_org	Brucella ceti B1/94
1	Cellular_org	Brucella ceti M13/05/1
1	Cellular_org	Brucella ceti M490/95/1
1	Cellular_org	Brucella ceti M644/93/1
1	Cellular_org	Brucella ceti str. Cudo
1	Cellular_org	Brucella melitensis
1	Cellular_org	Brucella melitensis ATCC 23457
1	Cellular_org	Brucella melitensis biovar Abortus 2308
1	Cellular_org	Brucella melitensis bv. 1 str. 16M
1	Cellular_org	Brucella melitensis bv. 1 str. Rev.1
1	Cellular_org	Brucella melitensis bv. 2 str. 63/9
1	Cellular_org	Brucella melitensis bv. 3 str. Ether
1	Cellular_org	Brucella microti
1	Cellular_org	Brucella microti CCM 4915
1	Cellular_org	Brucella neotomae
1	Cellular_org	Brucella neotomae 5K33
1	Cellular_org	Brucella ovis
1	Cellular_org	Brucella ovis ATCC 25840
1	Cellular_org	Brucella pinnipedialis
1	Cellular_org	Brucella pinnipedialis B2/94
1	Cellular_org	Brucella pinnipedialis M163/99/10
1	Cellular_org	Brucella pinnipedialis M292/94/1
1	Cellular_org	Brucella sp. 83/13
1	Cellular_org	Brucella sp. BO1
1	Cellular_org	Brucella sp. BO2
1	Cellular_org	Brucella sp. F5/99
1	Cellular_org	Brucella sp. NF 2653
1	Cellular_org	Brucella sp. NVSL 07-0026
1	Cellular_org	Brucella suis
1	Cellular_org	Brucella suis 1330
1	Cellular_org	Brucella suis ATCC 23445
1	Cellular_org	Brucella suis bv. 3 str. 686
1	Cellular_org	Brucella suis bv. 4 str. 40
1	Cellular_org	Brucella suis bv. 5 str. 513
1	Cellular_org	Buchnera aphidicola
1	Cellular_org	Buchnera aphidicola (Acyrthosiphon pisum)
1	Cellular_org	Buchnera aphidicola (Cinara cedri)
1	Cellular_org	Buchnera aphidicola (Schizaphis graminum)
1	Cellular_org	Buchnera aphidicola str. 5A (Acyrthosiphon pisum)
1	Cellular_org	Buchnera aphidicola str. APS (Acyrthosiphon pisum)

1	Cellular_org	Buchnera aphidicola str. Bp (Baizongia pistaciae)
1	Cellular_org	Buchnera aphidicola str. Cc (Cinara cedri)
1	Cellular_org	Buchnera aphidicola str. LSR1 (Acyrthosiphon pisum)
1	Cellular_org	Buchnera aphidicola str. Sg (Schizaphis graminum)
1	Cellular_org	Buchnera aphidicola str. Tuc7 (Acyrthosiphon pisum)
1	Cellular_org	Bulleidia extructa W1219
1	Cellular_org	Burkholderia ambifaria
1	Cellular_org	Burkholderia ambifaria AMMD
1	Cellular_org	Burkholderia ambifaria IOP40-10
1	Cellular_org	Burkholderia ambifaria MC40-6
1	Cellular_org	Burkholderia ambifaria MEX-5
1	Cellular_org	Burkholderia cenocepacia
1	Cellular_org	Burkholderia cenocepacia AU 1054
1	Cellular_org	Burkholderia cenocepacia HI2424
1	Cellular_org	Burkholderia cenocepacia J2315
1	Cellular_org	Burkholderia cenocepacia MC0-3
1	Cellular_org	Burkholderia cenocepacia PC184
1	Cellular_org	Burkholderia cepacia
1	Cellular_org	Burkholderia dolosa
1	Cellular_org	Burkholderia dolosa AUO158
1	Cellular_org	Burkholderia glumae
1	Cellular_org	Burkholderia glumae BGR1
1	Cellular_org	Burkholderia graminis
1	Cellular_org	Burkholderia graminis C4D1M
1	Cellular_org	Burkholderia mallei
1	Cellular_org	Burkholderia mallei 2002721280
1	Cellular_org	Burkholderia mallei ATCC 10399
1	Cellular_org	Burkholderia mallei ATCC 23344
1	Cellular_org	Burkholderia mallei FMH
1	Cellular_org	Burkholderia mallei GB8 horse 4
1	Cellular_org	Burkholderia mallei JHU
1	Cellular_org	Burkholderia mallei NCTC 10229
1	Cellular_org	Burkholderia mallei NCTC 10247
1	Cellular_org	Burkholderia mallei PRL-20
1	Cellular_org	Burkholderia mallei SAVP1
1	Cellular_org	Burkholderia multivorans
1	Cellular_org	Burkholderia multivorans ATCC 17616
1	Cellular_org	Burkholderia multivorans CGD1
1	Cellular_org	Burkholderia multivorans CGD2
1	Cellular_org	Burkholderia multivorans CGD2M
1	Cellular_org	Burkholderia oklahomensis C6786
1	Cellular_org	Burkholderia oklahomensis EO147
1	Cellular_org	Burkholderia phymatum

1	Cellular_org	Burkholderia phymatum STM815
1	Cellular_org	Burkholderia phytofirmans
1	Cellular_org	Burkholderia phytofirmans PsJN
1	Cellular_org	Burkholderia pseudomallei
1	Cellular_org	Burkholderia pseudomallei 1106a
1	Cellular_org	Burkholderia pseudomallei 1106b
1	Cellular_org	Burkholderia pseudomallei 112
1	Cellular_org	Burkholderia pseudomallei 14
1	Cellular_org	Burkholderia pseudomallei 1655
1	Cellular_org	Burkholderia pseudomallei 1710a
1	Cellular_org	Burkholderia pseudomallei 1710b
1	Cellular_org	Burkholderia pseudomallei 305
1	Cellular_org	Burkholderia pseudomallei 406e
1	Cellular_org	Burkholderia pseudomallei 576
1	Cellular_org	Burkholderia pseudomallei 668
1	Cellular_org	Burkholderia pseudomallei 7894
1	Cellular_org	Burkholderia pseudomallei 9
1	Cellular_org	Burkholderia pseudomallei 91
1	Cellular_org	Burkholderia pseudomallei B7210
1	Cellular_org	Burkholderia pseudomallei BCC215
1	Cellular_org	Burkholderia pseudomallei DM98
1	Cellular_org	Burkholderia pseudomallei K96243
1	Cellular_org	Burkholderia pseudomallei MSHR346
1	Cellular_org	Burkholderia pseudomallei NCTC 13177
1	Cellular_org	Burkholderia pseudomallei Pakistan 9
1	Cellular_org	Burkholderia pseudomallei Pasteur 52237
1	Cellular_org	Burkholderia pseudomallei S13
1	Cellular_org	Burkholderia rhizoxinica
1	Cellular_org	Burkholderia rhizoxinica HKI 454
1	Cellular_org	Burkholderia sp. 383
1	Cellular_org	Burkholderia sp. CCGE1001
1	Cellular_org	Burkholderia sp. CCGE1002
1	Cellular_org	Burkholderia sp. CCGE1003
1	Cellular_org	Burkholderia sp. Ch1-1
1	Cellular_org	Burkholderia sp. H160
1	Cellular_org	Burkholderia thailandensis
1	Cellular_org	Burkholderia thailandensis Bt4
1	Cellular_org	Burkholderia thailandensis E264
1	Cellular_org	Burkholderia thailandensis MSMB43
1	Cellular_org	Burkholderia thailandensis TXDOH
1	Cellular_org	Burkholderia ubonensis
1	Cellular_org	Burkholderia ubonensis Bu
1	Cellular_org	Burkholderia vietnamiensis

1	Cellular_org	Burkholderia vietnamiensis G4
1	Cellular_org	Burkholderia xenovorans
1	Cellular_org	Burkholderia xenovorans LB400
1	Cellular_org	Burkholderiales bacterium 1_1_47
1	Cellular_org	Butyrivibrio crossotus DSM 2876
1	Cellular_org	Butyrivibrio fibrisolvens
1	Cellular_org	Butyrivibrio fibrisolvens OB157
1	Cellular_org	Butyrivibrio proteoclasticus B316
1	Cellular_org	Caedibacter taeniospiralis
1	Cellular_org	Caldicellulosiruptor bescii DSM 6725
1	Cellular_org	Caldicellulosiruptor lactoaceticus
1	Cellular_org	Caldicellulosiruptor obsidiansis OB47
1	Cellular_org	Caldicellulosiruptor saccharolyticus
1	Cellular_org	Caldicellulosiruptor saccharolyticus DSM 8903
1	Cellular_org	Caminibacter mediatlanticus
1	Cellular_org	Caminibacter mediatlanticus TB-2
1	Cellular_org	Campylobacter coli
1	Cellular_org	Campylobacter coli JV20
1	Cellular_org	Campylobacter coli RM2228
1	Cellular_org	Campylobacter concisus
1	Cellular_org	Campylobacter concisus 13826
1	Cellular_org	Campylobacter curvus
1	Cellular_org	Campylobacter curvus 525.92
1	Cellular_org	Campylobacter fetus
1	Cellular_org	Campylobacter fetus subsp. fetus
1	Cellular_org	Campylobacter fetus subsp. fetus 82-40
1	Cellular_org	Campylobacter fetus subsp. venerealis
1	Cellular_org	Campylobacter fetus subsp. venerealis str. Azul-94
1	Cellular_org	Campylobacter gracilis
1	Cellular_org	Campylobacter gracilis RM3268
1	Cellular_org	Campylobacter hominis
1	Cellular_org	Campylobacter hominis ATCC BAA-381
1	Cellular_org	Campylobacter jejuni
1	Cellular_org	Campylobacter jejuni RM1221
1	Cellular_org	Campylobacter jejuni subsp. doylei
1	Cellular_org	Campylobacter jejuni subsp. doylei 269.97
1	Cellular_org	Campylobacter jejuni subsp. jejuni
1	Cellular_org	Campylobacter jejuni subsp. jejuni 1336
1	Cellular_org	Campylobacter jejuni subsp. jejuni 260.94
1	Cellular_org	Campylobacter jejuni subsp. jejuni 414
1	Cellular_org	Campylobacter jejuni subsp. jejuni 81-176
1	Cellular_org	Campylobacter jejuni subsp. jejuni 81116
1	Cellular_org	Campylobacter jejuni subsp. jejuni 84-25

1	Cellular_org	Campylobacter jejuni subsp. jejuni BH-01-0142
1	Cellular_org	Campylobacter jejuni subsp. jejuni CF93-6
1	Cellular_org	Campylobacter jejuni subsp. jejuni CG8421
1	Cellular_org	Campylobacter jejuni subsp. jejuni CG8486
1	Cellular_org	Campylobacter jejuni subsp. jejuni HB93-13
1	Cellular_org	Campylobacter jejuni subsp. jejuni NCTC 11168
1	Cellular_org	Campylobacter lari
1	Cellular_org	Campylobacter lari RM2100
1	Cellular_org	Campylobacter rectus
1	Cellular_org	Campylobacter rectus RM3267
1	Cellular_org	Campylobacter showae
1	Cellular_org	Campylobacter showae RM3277
1	Cellular_org	Campylobacter upsaliensis
1	Cellular_org	Campylobacter upsaliensis RM3195
1	Cellular_org	Campylobacterales bacterium GD 1
1	Cellular_org	Candidatus Accumulibacter phosphatis clade IIA str. UW-1
1	Cellular_org	Candidatus Amoebophilus asiaticus
1	Cellular_org	Candidatus Amoebophilus asiaticus 5a2
1	Cellular_org	Candidatus Azobacteroides pseudotrichonymphae genomovar. CFP2
1	Cellular_org	Candidatus Baumannia cicadellinicola
1	Cellular_org	Candidatus Blochmannia floridanus
1	Cellular_org	Candidatus Blochmannia pennsylvanicus
1	Cellular_org	Candidatus Blochmannia pennsylvanicus str. BPEN
1	Cellular_org	Candidatus Blochmannia vafer
1	Cellular_org	Candidatus Carsonella ruddii
1	Cellular_org	Candidatus Carsonella ruddii PV
1	Cellular_org	Candidatus Cloacamonas acidaminovorans
1	Cellular_org	Candidatus Desulforudis audaxviator MP104C
1	Cellular_org	Candidatus Hamiltonella defensa
1	Cellular_org	Candidatus Hamiltonella defensa 5AT (Acyrthosiphon pisum)
1	Cellular_org	Candidatus Hodgkinia cicadicola Dsem
1	Cellular_org	Candidatus Koribacter versatilis Ellin345
1	Cellular_org	Candidatus Liberibacter asiaticus
1	Cellular_org	Candidatus Liberibacter asiaticus str. psy62
1	Cellular_org	Candidatus Liberibacter solanacearum
1	Cellular_org	Candidatus Nitrospira defluvii
1	Cellular_org	Candidatus Pelagibacter sp. HTCC7211
1	Cellular_org	Candidatus Pelagibacter ubique
1	Cellular_org	Candidatus Pelagibacter ubique HTCC1002
1	Cellular_org	Candidatus Pelagibacter ubique HTCC1062
1	Cellular_org	Candidatus Phytoplasma australiense

1	Cellular_org	Candidatus Phytoplasma mali
1	Cellular_org	Candidatus Poribacteria sp. WGA-A3
1	Cellular_org	Candidatus Protochlamydia amoebophila UWE25
1	Cellular_org	Candidatus Puniceispirillum marinum IMCC1322
1	Cellular_org	Candidatus Regiella insecticola
1	Cellular_org	Candidatus Regiella insecticola LSR1
1	Cellular_org	Candidatus Rickettsia amblyommii
1	Cellular_org	Candidatus Riesia pediculicola
1	Cellular_org	Candidatus Riesia pediculicola USDA
1	Cellular_org	Candidatus Ruthia magnifica str. Cm (Calyptogena magnifica)
1	Cellular_org	Candidatus Solibacter usitatus Ellin6076
1	Cellular_org	Candidatus Sulcia muelleri CARI
1	Cellular_org	Candidatus Sulcia muelleri DMIN
1	Cellular_org	Candidatus Sulcia muelleri GWSS
1	Cellular_org	Candidatus Sulcia muelleri SMDSEM
1	Cellular_org	Candidatus Sulcia muelleri str. Hc (Homalodisca coagulata)
1	Cellular_org	Candidatus Vesicomyosocius okutanii HA
1	Cellular_org	Candidatus Zinderia insecticola CARI
1	Cellular_org	Capnocytophaga canimorsus
1	Cellular_org	Capnocytophaga gingivalis
1	Cellular_org	Capnocytophaga gingivalis ATCC 33624
1	Cellular_org	Capnocytophaga ochracea
1	Cellular_org	Capnocytophaga ochracea DSM 7271
1	Cellular_org	Capnocytophaga sputigena
1	Cellular_org	Carboxydibrachium pacificum DSM 12653
1	Cellular_org	Carboxydothermus hydrogenoformans
1	Cellular_org	Carboxydothermus hydrogenoformans Z-2901
1	Cellular_org	Cardiobacterium hominis ATCC 15826
1	Cellular_org	Carnobacterium sp. AT7
1	Cellular_org	Catenibacterium mitsuokai DSM 15897
1	Cellular_org	Catenulispora acidiphila DSM 44928
1	Cellular_org	Catonella morbi ATCC 51271
1	Cellular_org	Caulobacter crescentus CB15
1	Cellular_org	Caulobacter crescentus NA1000
1	Cellular_org	Caulobacter segnis
1	Cellular_org	Caulobacter segnis ATCC 21756
1	Cellular_org	Caulobacter sp. K31
1	Cellular_org	Caulobacter vibrioides
1	Cellular_org	Cellulomonas flavigena
1	Cellular_org	Cellulomonas flavigena DSM 20109
1	Cellular_org	Cellvibrio japonicus

1	Cellular_org	Cellvibrio japonicus Ueda107
1	Cellular_org	Chelativorans sp. BNC1
1	Cellular_org	Chitinophaga pinensis
1	Cellular_org	Chitinophaga pinensis DSM 2588
1	Cellular_org	Chlamydia muridarum MopnTet14
1	Cellular_org	Chlamydia muridarum Nigg
1	Cellular_org	Chlamydia muridarum Weiss
1	Cellular_org	Chlamydia trachomatis
1	Cellular_org	Chlamydia trachomatis 434/Bu
1	Cellular_org	Chlamydia trachomatis 6276
1	Cellular_org	Chlamydia trachomatis 6276s
1	Cellular_org	Chlamydia trachomatis 70
1	Cellular_org	Chlamydia trachomatis 70s
1	Cellular_org	Chlamydia trachomatis A/HAR-13
1	Cellular_org	Chlamydia trachomatis B/Jali20/OT
1	Cellular_org	Chlamydia trachomatis B/TZ1A828/OT
1	Cellular_org	Chlamydia trachomatis D(s)2923
1	Cellular_org	Chlamydia trachomatis D/UW-3/CX
1	Cellular_org	Chlamydia trachomatis L2b/UCH-1/proctitis
1	Cellular_org	Chlamydia trachomatis L2tet1
1	Cellular_org	Chlamydophila abortus
1	Cellular_org	Chlamydophila abortus S26/3
1	Cellular_org	Chlamydophila caviae
1	Cellular_org	Chlamydophila caviae GPIC
1	Cellular_org	Chlamydophila felis
1	Cellular_org	Chlamydophila felis Fe/C-56
1	Cellular_org	Chlamydophila pneumoniae
1	Cellular_org	Chlamydophila pneumoniae AR39
1	Cellular_org	Chlamydophila pneumoniae CWL029
1	Cellular_org	Chlamydophila pneumoniae J138
1	Cellular_org	Chlamydophila pneumoniae TW-183
1	Cellular_org	Chlamydophila psittaci
1	Cellular_org	Chlorobaculum parvum
1	Cellular_org	Chlorobaculum parvum NCIB 8327
1	Cellular_org	Chlorobaculum tepidum
1	Cellular_org	Chlorobium chlorochromatii CaD3
1	Cellular_org	Chlorobium ferrooxidans DSM 13031
1	Cellular_org	Chlorobium limicola
1	Cellular_org	Chlorobium limicola DSM 245
1	Cellular_org	Chlorobium luteolum DSM 273
1	Cellular_org	Chlorobium phaeobacteroides
1	Cellular_org	Chlorobium phaeobacteroides BS1
1	Cellular_org	Chlorobium phaeobacteroides DSM 266

1	Cellular_org	Chlorobium phaeovibrioides
1	Cellular_org	Chlorobium phaeovibrioides DSM 265
1	Cellular_org	Chlorobium tepidum TLS
1	Cellular_org	Chloroflexus aggregans DSM 9485
1	Cellular_org	Chloroflexus aurantiacus
1	Cellular_org	Chloroflexus aurantiacus J-10-fl
1	Cellular_org	Chloroflexus sp. Y-400-fl
1	Cellular_org	Chloroherpeton thalassium
1	Cellular_org	Chloroherpeton thalassium ATCC 35110
1	Cellular_org	Chromobacterium violaceum
1	Cellular_org	Chromobacterium violaceum ATCC 12472
1	Cellular_org	Chromohalobacter salexigens
1	Cellular_org	Chromohalobacter salexigens DSM 3043
1	Cellular_org	Chryseobacterium gleum
1	Cellular_org	Chryseobacterium gleum ATCC 35910
1	Cellular_org	Chthoniobacter flavus Ellin428
1	Cellular_org	Citreicella sp. SE45
1	Cellular_org	Citrobacter freundii
1	Cellular_org	Citrobacter koseri
1	Cellular_org	Citrobacter koseri ATCC BAA-895
1	Cellular_org	Citrobacter rodentium
1	Cellular_org	Citrobacter rodentium ICC168
1	Cellular_org	Citrobacter sp. 30_2
1	Cellular_org	Citrobacter youngae
1	Cellular_org	Citrobacter youngae ATCC 29220
1	Cellular_org	Citromicrobium bathyomarinum JL354
1	Cellular_org	Clavibacter michiganensis
1	Cellular_org	Clavibacter michiganensis subsp. michiganensis
1	Cellular_org	Clavibacter michiganensis subsp. michiganensis NCPPB 382
1	Cellular_org	Clavibacter michiganensis subsp. sepedonicus
1	Cellular_org	Clostridiales genomosp. BVAB3 str. UPII9-5
1	Cellular_org	Clostridium acetobutylicum
1	Cellular_org	Clostridium acetobutylicum ATCC 824
1	Cellular_org	Clostridium asparagiforme DSM 15981
1	Cellular_org	Clostridium bartlettii DSM 16795
1	Cellular_org	Clostridium beijerinckii
1	Cellular_org	Clostridium beijerinckii NCIMB 8052
1	Cellular_org	Clostridium bolteae
1	Cellular_org	Clostridium bolteae ATCC BAA-613
1	Cellular_org	Clostridium botulinum
1	Cellular_org	Clostridium botulinum A
1	Cellular_org	Clostridium botulinum A str. ATCC 19397

1	Cellular_org	Clostridium botulinum A str. ATCC 3502
1	Cellular_org	Clostridium botulinum A str. Hall
1	Cellular_org	Clostridium botulinum A2 str. Kyoto
1	Cellular_org	Clostridium botulinum A3 str. Loch Maree
1	Cellular_org	Clostridium botulinum B
1	Cellular_org	Clostridium botulinum B str. Eklund 17B
1	Cellular_org	Clostridium botulinum B1 str. Okra
1	Cellular_org	Clostridium botulinum Ba4 str. 657
1	Cellular_org	Clostridium botulinum Bf
1	Cellular_org	Clostridium botulinum C
1	Cellular_org	Clostridium botulinum C str. Eklund
1	Cellular_org	Clostridium botulinum D
1	Cellular_org	Clostridium botulinum D str. 1873
1	Cellular_org	Clostridium botulinum E
1	Cellular_org	Clostridium botulinum E1 str. 'BoNT E Beluga'
1	Cellular_org	Clostridium botulinum E3 str. Alaska E43
1	Cellular_org	Clostridium botulinum F str. Langeland
1	Cellular_org	Clostridium botulinum NCTC 2916
1	Cellular_org	Clostridium butyricum
1	Cellular_org	Clostridium butyricum 5521
1	Cellular_org	Clostridium butyricum E4 str. BoNT E BL5262
1	Cellular_org	Clostridium carboxidivorans P7
1	Cellular_org	Clostridium cellulolyticum
1	Cellular_org	Clostridium cellulolyticum H10
1	Cellular_org	Clostridium cellulovorans
1	Cellular_org	Clostridium cellulovorans 743B
1	Cellular_org	Clostridium difficile
1	Cellular_org	Clostridium difficile 630
1	Cellular_org	Clostridium difficile ATCC 43255
1	Cellular_org	Clostridium difficile CD196
1	Cellular_org	Clostridium difficile CIP 107932
1	Cellular_org	Clostridium difficile NAP07
1	Cellular_org	Clostridium difficile NAP08
1	Cellular_org	Clostridium difficile QCD-23m63
1	Cellular_org	Clostridium difficile QCD-32g58
1	Cellular_org	Clostridium difficile QCD-37x79
1	Cellular_org	Clostridium difficile QCD-63q42
1	Cellular_org	Clostridium difficile QCD-66c26
1	Cellular_org	Clostridium difficile QCD-76w55
1	Cellular_org	Clostridium difficile QCD-97b34
1	Cellular_org	Clostridium difficile R20291
1	Cellular_org	Clostridium hathewayi
1	Cellular_org	Clostridium hathewayi DSM 13479

1	Cellular_org	Clostridium hiranonis DSM 13275
1	Cellular_org	Clostridium hylemonae DSM 15053
1	Cellular_org	Clostridium kluyveri
1	Cellular_org	Clostridium kluyveri DSM 555
1	Cellular_org	Clostridium kluyveri NBRC 12016
1	Cellular_org	Clostridium lentocellum DSM 5427
1	Cellular_org	Clostridium leptum DSM 753
1	Cellular_org	Clostridium ljungdahlii DSM 13528
1	Cellular_org	Clostridium methylpentosum DSM 5476
1	Cellular_org	Clostridium nexile DSM 1787
1	Cellular_org	Clostridium novyi
1	Cellular_org	Clostridium novyi NT
1	Cellular_org	Clostridium papyrosolvens DSM 2782
1	Cellular_org	Clostridium perfringens
1	Cellular_org	Clostridium perfringens ATCC 13124
1	Cellular_org	Clostridium perfringens B
1	Cellular_org	Clostridium perfringens B str. ATCC 3626
1	Cellular_org	Clostridium perfringens C
1	Cellular_org	Clostridium perfringens C str. JGS1495
1	Cellular_org	Clostridium perfringens CPE str. F4969
1	Cellular_org	Clostridium perfringens D
1	Cellular_org	Clostridium perfringens D str. JGS1721
1	Cellular_org	Clostridium perfringens E str. JGS1987
1	Cellular_org	Clostridium perfringens NCTC 8239
1	Cellular_org	Clostridium perfringens SM101
1	Cellular_org	Clostridium perfringens str. 13
1	Cellular_org	Clostridium phytofermentans ISDg
1	Cellular_org	Clostridium ramosum
1	Cellular_org	Clostridium ramosum DSM 1402
1	Cellular_org	Clostridium saccharolyticum WM1
1	Cellular_org	Clostridium scindens
1	Cellular_org	Clostridium scindens ATCC 35704
1	Cellular_org	Clostridium sp. 7_2_43FAA
1	Cellular_org	Clostridium sp. L2-50
1	Cellular_org	Clostridium sp. M62/1
1	Cellular_org	Clostridium sp. MCF-1
1	Cellular_org	Clostridium sp. SS2/1
1	Cellular_org	Clostridium spiroforme
1	Cellular_org	Clostridium spiroforme DSM 1552
1	Cellular_org	Clostridium sporogenes
1	Cellular_org	Clostridium sporogenes ATCC 15579
1	Cellular_org	Clostridium sticklandii
1	Cellular_org	Clostridium sticklandii DSM 519

1	Cellular_org	Clostridium symbiosum
1	Cellular_org	Clostridium tetani
1	Cellular_org	Clostridium tetani E88
1	Cellular_org	Clostridium thermocellum
1	Cellular_org	Clostridium thermocellum ATCC 27405
1	Cellular_org	Clostridium thermocellum DSM 2360
1	Cellular_org	Clostridium thermocellum JW20
1	Cellular_org	Collimonas fungivorans
1	Cellular_org	Collinsella aerofaciens
1	Cellular_org	Collinsella aerofaciens ATCC 25986
1	Cellular_org	Collinsella intestinalis DSM 13280
1	Cellular_org	Collinsella stercoris DSM 13279
1	Cellular_org	Colwellia psychrerythraea
1	Cellular_org	Colwellia psychrerythraea 34H
1	Cellular_org	Comamonas sp. CNB-1
1	Cellular_org	Comamonas testosteroni
1	Cellular_org	Comamonas testosteroni CNB-2
1	Cellular_org	Comamonas testosteroni KF-1
1	Cellular_org	Comamonas testosteroni S44
1	Cellular_org	Conexibacter woesei DSM 14684
1	Cellular_org	Congregibacter litoralis KT71
1	Cellular_org	Coprobacillus sp. D7
1	Cellular_org	Coprococcus comes ATCC 27758
1	Cellular_org	Coprococcus eutactus ATCC 27759
1	Cellular_org	Coprothermobacter proteolyticus DSM 5265
1	Cellular_org	Coraliomargarita akajimensis DSM 45221
1	Cellular_org	Corynebacterium accolens
1	Cellular_org	Corynebacterium accolens ATCC 49725
1	Cellular_org	Corynebacterium accolens ATCC 49726
1	Cellular_org	Corynebacterium ammoniagenes
1	Cellular_org	Corynebacterium ammoniagenes DSM 20306
1	Cellular_org	Corynebacterium amycolatum
1	Cellular_org	Corynebacterium amycolatum SK46
1	Cellular_org	Corynebacterium aurimucosum
1	Cellular_org	Corynebacterium aurimucosum ATCC 700975
1	Cellular_org	Corynebacterium callunae
1	Cellular_org	Corynebacterium casei
1	Cellular_org	Corynebacterium diphtheriae
1	Cellular_org	Corynebacterium diphtheriae NCTC 13129
1	Cellular_org	Corynebacterium efficiens
1	Cellular_org	Corynebacterium efficiens YS-314
1	Cellular_org	Corynebacterium genitalium ATCC 33030
1	Cellular_org	Corynebacterium glucuronolyticum

1	Cellular_org	Corynebacterium glucuronolyticum ATCC 51866
1	Cellular_org	Corynebacterium glucuronolyticum ATCC 51867
1	Cellular_org	Corynebacterium glutamicum
1	Cellular_org	Corynebacterium glutamicum ATCC 13032
1	Cellular_org	Corynebacterium glutamicum R
1	Cellular_org	Corynebacterium jeikeium
1	Cellular_org	Corynebacterium jeikeium ATCC 43734
1	Cellular_org	Corynebacterium jeikeium K411
1	Cellular_org	Corynebacterium kroppenstedtii DSM 44385
1	Cellular_org	Corynebacterium lipophiloflavum
1	Cellular_org	Corynebacterium lipophiloflavum DSM 44291
1	Cellular_org	Corynebacterium matruchotii
1	Cellular_org	Corynebacterium matruchotii ATCC 14266
1	Cellular_org	Corynebacterium matruchotii ATCC 33806
1	Cellular_org	Corynebacterium pseudogenitalium
1	Cellular_org	Corynebacterium pseudogenitalium ATCC 33035
1	Cellular_org	Corynebacterium pseudotuberculosis
1	Cellular_org	Corynebacterium pseudotuberculosis FRC41
1	Cellular_org	Corynebacterium renale
1	Cellular_org	Corynebacterium resistens DSM 45100
1	Cellular_org	Corynebacterium sp. L2-79-05
1	Cellular_org	Corynebacterium striatum
1	Cellular_org	Corynebacterium striatum ATCC 6940
1	Cellular_org	Corynebacterium tuberculostearicum
1	Cellular_org	Corynebacterium tuberculostearicum SK141
1	Cellular_org	Corynebacterium urealyticum
1	Cellular_org	Corynebacterium urealyticum DSM 7109
1	Cellular_org	Corynebacterium variabile
1	Cellular_org	Coxiella burnetii
1	Cellular_org	Coxiella burnetii 'MSU Goat Q177'
1	Cellular_org	Coxiella burnetii CbuG_Q212
1	Cellular_org	Coxiella burnetii CbuK_Q154
1	Cellular_org	Coxiella burnetii Dugway 5J108-111
1	Cellular_org	Coxiella burnetii RSA 331
1	Cellular_org	Coxiella burnetii RSA 334
1	Cellular_org	Coxiella burnetii RSA 493
1	Cellular_org	Croceibacter atlanticus HTCC2559
1	Cellular_org	Crocosphaera watsonii
1	Cellular_org	Crocosphaera watsonii WH 8501
1	Cellular_org	Cronobacter sakazakii
1	Cellular_org	Cronobacter sakazakii ATCC BAA-894
1	Cellular_org	Cronobacter turicensis
1	Cellular_org	Cronobacter turicensis z3032

1	Cellular_org	Cryptobacterium curtum DSM 15641
1	Cellular_org	Cupriavidus metallidurans
1	Cellular_org	Cupriavidus metallidurans CH34
1	Cellular_org	Cupriavidus necator
1	Cellular_org	Cupriavidus taiwanensis
1	Cellular_org	Cyanobium sp. PCC 7001
1	Cellular_org	Cyanothece sp. ATCC 51142
1	Cellular_org	Cyanothece sp. CCY0110
1	Cellular_org	Cyanothece sp. PCC 7424
1	Cellular_org	Cyanothece sp. PCC 7425
1	Cellular_org	Cyanothece sp. PCC 7822
1	Cellular_org	Cyanothece sp. PCC 8801
1	Cellular_org	Cyanothece sp. PCC 8802
1	Cellular_org	Cylindrospermopsis raciborskii
1	Cellular_org	Cylindrospermopsis raciborskii CS-505
1	Cellular_org	Cylindrospermum sp. A1345
1	Cellular_org	Cytophaga hutchinsonii
1	Cellular_org	Cytophaga hutchinsonii ATCC 33406
1	Cellular_org	Dechloromonas aromatica RCB
1	Cellular_org	Deferribacter desulfuricans SSM1
1	Cellular_org	Dehalococcoides ethenogenes
1	Cellular_org	Dehalococcoides ethenogenes 195
1	Cellular_org	Dehalococcoides sp. BAV1
1	Cellular_org	Dehalococcoides sp. CBDB1
1	Cellular_org	Dehalococcoides sp. GT
1	Cellular_org	Dehalococcoides sp. VS
1	Cellular_org	Dehalogenimonas lykanthroporepellens BL-DC-9
1	Cellular_org	Deinococcus deserti
1	Cellular_org	Deinococcus deserti VCD115
1	Cellular_org	Deinococcus geothermalis
1	Cellular_org	Deinococcus geothermalis DSM 11300
1	Cellular_org	Deinococcus radiodurans
1	Cellular_org	Deinococcus radiodurans R1
1	Cellular_org	Deinococcus radiopugnans ATCC 19172
1	Cellular_org	Delftia acidovorans
1	Cellular_org	Delftia acidovorans SPH-1
1	Cellular_org	Denitrovibrio acetiphilus DSM 12809
1	Cellular_org	Dermacoccus sp. Ellin185
1	Cellular_org	Desulfarculus baarsii DSM 2075
1	Cellular_org	Desulfatibacillum alkenivorans
1	Cellular_org	Desulfatibacillum alkenivorans AK-01
1	Cellular_org	Desulfitobacterium hafniense
1	Cellular_org	Desulfitobacterium hafniense DCB-2

1	Cellular_org	Desulfitobacterium hafniense Y51
1	Cellular_org	Desulfobacterium autotrophicum
1	Cellular_org	Desulfobacterium autotrophicum HRM2
1	Cellular_org	Desulfobulbus propionicus
1	Cellular_org	Desulfobulbus propionicus DSM 2032
1	Cellular_org	Desulfococcus oleovorans Hxd3
1	Cellular_org	Desulfohalobium retbaense DSM 5692
1	Cellular_org	Desulfomicrobium baculatum
1	Cellular_org	Desulfomicrobium baculatum DSM 4028
1	Cellular_org	Desulfonatronospira thiodismutans ASO3-1
1	Cellular_org	Desulfotalea psychrophila LSv54
1	Cellular_org	Desulfotomaculum acetoxidans
1	Cellular_org	Desulfotomaculum acetoxidans DSM 771
1	Cellular_org	Desulfotomaculum nigrificans
1	Cellular_org	Desulfotomaculum nigrificans DSM 574
1	Cellular_org	Desulfotomaculum reducens MI-1
1	Cellular_org	Desulfovibrio aespoeensis
1	Cellular_org	Desulfovibrio aespoeensis Aspo-2
1	Cellular_org	Desulfovibrio desulfuricans
1	Cellular_org	Desulfovibrio desulfuricans subsp. desulfuricans
1	Cellular_org	Desulfovibrio desulfuricans subsp. desulfuricans str. ATCC 27774
1	Cellular_org	Desulfovibrio desulfuricans subsp. desulfuricans str. G20
1	Cellular_org	Desulfovibrio fructosovorans
1	Cellular_org	Desulfovibrio fructosovorans JJ
1	Cellular_org	Desulfovibrio magneticus RS-1
1	Cellular_org	Desulfovibrio piger
1	Cellular_org	Desulfovibrio piger ATCC 29098
1	Cellular_org	Desulfovibrio salexigens
1	Cellular_org	Desulfovibrio salexigens DSM 2638
1	Cellular_org	Desulfovibrio sp. 3_1_syn3
1	Cellular_org	Desulfovibrio sp. FW1012B
1	Cellular_org	Desulfovibrio vulgaris
1	Cellular_org	Desulfovibrio vulgaris DP4
1	Cellular_org	Desulfovibrio vulgaris str. 'Miyazaki F'
1	Cellular_org	Desulfovibrio vulgaris str. Hildenborough
1	Cellular_org	Desulfurivibrio alkaliphilus AHT2
1	Cellular_org	Desulfuromonas acetoxidans
1	Cellular_org	Desulfuromonas acetoxidans DSM 684
1	Cellular_org	Dethiobacter alkaliphilus AHT 1
1	Cellular_org	Dethiosulfovibrio peptidovorans DSM 11002
1	Cellular_org	Dialister invisus DSM 15470
1	Cellular_org	Dialister micraerophilus

1	Cellular_org	Dichelobacter nodosus
1	Cellular_org	Dichelobacter nodosus VCS1703A
1	Cellular_org	Dickeya dadantii
1	Cellular_org	Dickeya dadantii 3937
1	Cellular_org	Dickeya dadantii Ech586
1	Cellular_org	Dickeya dadantii Ech703
1	Cellular_org	Dickeya zeae
1	Cellular_org	Dickeya zeae Ech1591
1	Cellular_org	Dictyoglomus thermophilum
1	Cellular_org	Dictyoglomus thermophilum H-6-12
1	Cellular_org	Dictyoglomus turgidum DSM 6724
1	Cellular_org	Dietzia cinnamea
1	Cellular_org	Dinoroseobacter shibae DFL 12
1	Cellular_org	Dokdonia donghaensis
1	Cellular_org	Dokdonia donghaensis MED134
1	Cellular_org	Dorea formicigenerans ATCC 27755
1	Cellular_org	Dorea longicatena DSM 13814
1	Cellular_org	Dyadobacter fermentans DSM 18053
1	Cellular_org	Edwardsiella ictaluri
1	Cellular_org	Edwardsiella ictaluri 93-146
1	Cellular_org	Edwardsiella tarda
1	Cellular_org	Edwardsiella tarda ATCC 23685
1	Cellular_org	Edwardsiella tarda EIB202
1	Cellular_org	Eggerthella lenta
1	Cellular_org	Eggerthella lenta DSM 2243
1	Cellular_org	Ehrlichia canis
1	Cellular_org	Ehrlichia canis str. Jake
1	Cellular_org	Ehrlichia chaffeensis
1	Cellular_org	Ehrlichia chaffeensis str. Arkansas
1	Cellular_org	Ehrlichia chaffeensis str. Sapulpa
1	Cellular_org	Ehrlichia ruminantium str. Gardel
1	Cellular_org	Ehrlichia ruminantium str. Welgevonden
1	Cellular_org	Eikenella corrodens
1	Cellular_org	Eikenella corrodens ATCC 23834
1	Cellular_org	Elusimicrobium minutum Pei191
1	Cellular_org	Endoriftia persephone 'Hot96_1+Hot96_2'
1	Cellular_org	Enhydrobacter aerosaccus SK60
1	Cellular_org	Enterobacter aerogenes
1	Cellular_org	Enterobacter cancerogenus
1	Cellular_org	Enterobacter cancerogenus ATCC 35316
1	Cellular_org	Enterobacter cloacae
1	Cellular_org	Enterobacter cloacae SCF1
1	Cellular_org	Enterobacter cloacae subsp. cloacae

1	Cellular_org	Enterobacter cloacae subsp. cloacae ATCC 13047
1	Cellular_org	Enterobacter sp. 638
1	Cellular_org	Enterobacter sp. RFL1396
1	Cellular_org	Enterococcus casseliflavus
1	Cellular_org	Enterococcus casseliflavus EC10
1	Cellular_org	Enterococcus casseliflavus EC20
1	Cellular_org	Enterococcus casseliflavus EC30
1	Cellular_org	Enterococcus faecalis
1	Cellular_org	Enterococcus faecalis ATCC 29200
1	Cellular_org	Enterococcus faecalis ATCC 4200
1	Cellular_org	Enterococcus faecalis CH188
1	Cellular_org	Enterococcus faecalis D6
1	Cellular_org	Enterococcus faecalis DAPTO 512
1	Cellular_org	Enterococcus faecalis DS5
1	Cellular_org	Enterococcus faecalis E1Sol
1	Cellular_org	Enterococcus faecalis Fly1
1	Cellular_org	Enterococcus faecalis HH22
1	Cellular_org	Enterococcus faecalis HIP11704
1	Cellular_org	Enterococcus faecalis JH1
1	Cellular_org	Enterococcus faecalis Merz96
1	Cellular_org	Enterococcus faecalis OG1RF
1	Cellular_org	Enterococcus faecalis PC1.1
1	Cellular_org	Enterococcus faecalis R712
1	Cellular_org	Enterococcus faecalis S613
1	Cellular_org	Enterococcus faecalis T1
1	Cellular_org	Enterococcus faecalis T11
1	Cellular_org	Enterococcus faecalis T2
1	Cellular_org	Enterococcus faecalis T3
1	Cellular_org	Enterococcus faecalis T8
1	Cellular_org	Enterococcus faecalis TUSoD Ef11
1	Cellular_org	Enterococcus faecalis TX0102
1	Cellular_org	Enterococcus faecalis TX0104
1	Cellular_org	Enterococcus faecalis TX0109
1	Cellular_org	Enterococcus faecalis TX0411
1	Cellular_org	Enterococcus faecalis TX0635
1	Cellular_org	Enterococcus faecalis TX0855
1	Cellular_org	Enterococcus faecalis TX0860
1	Cellular_org	Enterococcus faecalis TX1322
1	Cellular_org	Enterococcus faecalis TX2134
1	Cellular_org	Enterococcus faecalis TX4248
1	Cellular_org	Enterococcus faecalis V583
1	Cellular_org	Enterococcus faecalis X98
1	Cellular_org	Enterococcus faecium

1	Cellular_org	Enterococcus faecium 1,141,733
1	Cellular_org	Enterococcus faecium 1,230,933
1	Cellular_org	Enterococcus faecium 1,231,408
1	Cellular_org	Enterococcus faecium 1,231,410
1	Cellular_org	Enterococcus faecium 1,231,501
1	Cellular_org	Enterococcus faecium 1,231,502
1	Cellular_org	Enterococcus faecium C68
1	Cellular_org	Enterococcus faecium Com12
1	Cellular_org	Enterococcus faecium Com15
1	Cellular_org	Enterococcus faecium D344SRF
1	Cellular_org	Enterococcus faecium DO
1	Cellular_org	Enterococcus faecium E1039
1	Cellular_org	Enterococcus faecium E1071
1	Cellular_org	Enterococcus faecium E1162
1	Cellular_org	Enterococcus faecium E1636
1	Cellular_org	Enterococcus faecium E1679
1	Cellular_org	Enterococcus faecium E980
1	Cellular_org	Enterococcus faecium PC4.1
1	Cellular_org	Enterococcus faecium TC 6
1	Cellular_org	Enterococcus faecium TX1330
1	Cellular_org	Enterococcus faecium U0317
1	Cellular_org	Enterococcus gallinarum
1	Cellular_org	Enterococcus gallinarum EG2
1	Cellular_org	Enterococcus italicus
1	Cellular_org	Epulopiscium sp. 'N.t. morphotype B'
1	Cellular_org	Erwinia amylovora
1	Cellular_org	Erwinia amylovora ATCC 49946
1	Cellular_org	Erwinia amylovora CFBP1430
1	Cellular_org	Erwinia billingiae
1	Cellular_org	Erwinia billingiae Eb661
1	Cellular_org	Erwinia pyrifoliae
1	Cellular_org	Erwinia pyrifoliae Ep1/96
1	Cellular_org	Erwinia sp. Ejp 556
1	Cellular_org	Erwinia tasmaniensis
1	Cellular_org	Erwinia tasmaniensis Et1/99
1	Cellular_org	Erysipelothrix rhusiopathiae
1	Cellular_org	Erysipelothrix rhusiopathiae ATCC 19414
1	Cellular_org	Erysipelotrichaceae bacterium 3_1_53
1	Cellular_org	Erysipelotrichaceae bacterium 5_2_54FAA
1	Cellular_org	Erythrobacter litoralis
1	Cellular_org	Erythrobacter litoralis HTCC2594
1	Cellular_org	Erythrobacter sp. NAP1
1	Cellular_org	Erythrobacter sp. SD-21

1	Cellular_org	Escherichia albertii
1	Cellular_org	Escherichia albertii TW07627
1	Cellular_org	Escherichia coli
1	Cellular_org	Escherichia coli 'BL21-Gold(DE3)pLysS AG'
1	Cellular_org	Escherichia coli 101-1
1	Cellular_org	Escherichia coli 1520
1	Cellular_org	Escherichia coli 1827-70
1	Cellular_org	Escherichia coli 536
1	Cellular_org	Escherichia coli 53638
1	Cellular_org	Escherichia coli 55989
1	Cellular_org	Escherichia coli 83972
1	Cellular_org	Escherichia coli APEC O1
1	Cellular_org	Escherichia coli ATCC 8739
1	Cellular_org	Escherichia coli B
1	Cellular_org	Escherichia coli B str. REL606
1	Cellular_org	Escherichia coli B088
1	Cellular_org	Escherichia coli B171
1	Cellular_org	Escherichia coli B185
1	Cellular_org	Escherichia coli B354
1	Cellular_org	Escherichia coli B7A
1	Cellular_org	Escherichia coli BW2952
1	Cellular_org	Escherichia coli CFT073
1	Cellular_org	Escherichia coli E110019
1	Cellular_org	Escherichia coli E22
1	Cellular_org	Escherichia coli E24377A
1	Cellular_org	Escherichia coli ED1a
1	Cellular_org	Escherichia coli ETEC 1392/75
1	Cellular_org	Escherichia coli ETEC H10407
1	Cellular_org	Escherichia coli F11
1	Cellular_org	Escherichia coli FVEC1302
1	Cellular_org	Escherichia coli FVEC1412
1	Cellular_org	Escherichia coli H299
1	Cellular_org	Escherichia coli H591
1	Cellular_org	Escherichia coli H736
1	Cellular_org	Escherichia coli HS
1	Cellular_org	Escherichia coli IAI1
1	Cellular_org	Escherichia coli IAI39
1	Cellular_org	Escherichia coli K-12
1	Cellular_org	Escherichia coli M605
1	Cellular_org	Escherichia coli M718
1	Cellular_org	Escherichia coli MS 107-1
1	Cellular_org	Escherichia coli MS 115-1
1	Cellular_org	Escherichia coli MS 116-1

1	Cellular_org	Escherichia coli MS 119-7
1	Cellular_org	Escherichia coli MS 124-1
1	Cellular_org	Escherichia coli MS 145-7
1	Cellular_org	Escherichia coli MS 146-1
1	Cellular_org	Escherichia coli MS 175-1
1	Cellular_org	Escherichia coli MS 182-1
1	Cellular_org	Escherichia coli MS 185-1
1	Cellular_org	Escherichia coli MS 187-1
1	Cellular_org	Escherichia coli MS 196-1
1	Cellular_org	Escherichia coli MS 198-1
1	Cellular_org	Escherichia coli MS 200-1
1	Cellular_org	Escherichia coli MS 21-1
1	Cellular_org	Escherichia coli MS 45-1
1	Cellular_org	Escherichia coli MS 69-1
1	Cellular_org	Escherichia coli MS 78-1
1	Cellular_org	Escherichia coli MS 84-1
1	Cellular_org	Escherichia coli NC101
1	Cellular_org	Escherichia coli O103:H2
1	Cellular_org	Escherichia coli O103:H2 str. 12009
1	Cellular_org	Escherichia coli O111:H-
1	Cellular_org	Escherichia coli O111:H- str. 11128
1	Cellular_org	Escherichia coli O127:H6
1	Cellular_org	Escherichia coli O127:H6 str. E2348/69
1	Cellular_org	Escherichia coli O157:H7
1	Cellular_org	Escherichia coli O157:H7 str. EC4024
1	Cellular_org	Escherichia coli O157:H7 str. EC4042
1	Cellular_org	Escherichia coli O157:H7 str. EC4045
1	Cellular_org	Escherichia coli O157:H7 str. EC4076
1	Cellular_org	Escherichia coli O157:H7 str. EC4113
1	Cellular_org	Escherichia coli O157:H7 str. EC4115
1	Cellular_org	Escherichia coli O157:H7 str. EC4196
1	Cellular_org	Escherichia coli O157:H7 str. EC4206
1	Cellular_org	Escherichia coli O157:H7 str. EC4401
1	Cellular_org	Escherichia coli O157:H7 str. EC4486
1	Cellular_org	Escherichia coli O157:H7 str. EC4501
1	Cellular_org	Escherichia coli O157:H7 str. EC508
1	Cellular_org	Escherichia coli O157:H7 str. EC869
1	Cellular_org	Escherichia coli O157:H7 str. FRIK2000
1	Cellular_org	Escherichia coli O157:H7 str. FRIK966
1	Cellular_org	Escherichia coli O157:H7 str. Sakai
1	Cellular_org	Escherichia coli O157:H7 str. TW14359
1	Cellular_org	Escherichia coli O157:H7 str. TW14588
1	Cellular_org	Escherichia coli O26:H-

1	Cellular_org	Escherichia coli O26:H11
1	Cellular_org	Escherichia coli O26:H11 str. 11368
1	Cellular_org	Escherichia coli O55:H7
1	Cellular_org	Escherichia coli O55:H7 str. CB9615
1	Cellular_org	Escherichia coli OP50
1	Cellular_org	Escherichia coli S88
1	Cellular_org	Escherichia coli SE11
1	Cellular_org	Escherichia coli SMS-3-5
1	Cellular_org	Escherichia coli TA143
1	Cellular_org	Escherichia coli TA206
1	Cellular_org	Escherichia coli TA271
1	Cellular_org	Escherichia coli TA280
1	Cellular_org	Escherichia coli UMN026
1	Cellular_org	Escherichia coli UTI89
1	Cellular_org	Escherichia coli Vir68
1	Cellular_org	Escherichia coli W
1	Cellular_org	Escherichia coli str. K-12 substr. DH10B
1	Cellular_org	Escherichia coli str. K-12 substr. MG1655
1	Cellular_org	Escherichia coli str. K-12 substr. W3110
1	Cellular_org	Escherichia fergusonii
1	Cellular_org	Escherichia fergusonii ATCC 35469
1	Cellular_org	Escherichia sp. 1_1_43
1	Cellular_org	Escherichia sp. 3_2_53FAA
1	Cellular_org	Escherichia sp. 4_1_40B
1	Cellular_org	Escherichia sp. Sflu5
1	Cellular_org	Ethanoligenens harbinense
1	Cellular_org	Ethanoligenens harbinense YUAN-3
1	Cellular_org	Eubacterium biforme DSM 3989
1	Cellular_org	Eubacterium cellulosolvens
1	Cellular_org	Eubacterium dolichum DSM 3991
1	Cellular_org	Eubacterium eligens ATCC 27750
1	Cellular_org	Eubacterium hallii
1	Cellular_org	Eubacterium hallii DSM 3353
1	Cellular_org	Eubacterium limosum
1	Cellular_org	Eubacterium limosum KIST612
1	Cellular_org	Eubacterium rectale
1	Cellular_org	Eubacterium rectale ATCC 33656
1	Cellular_org	Eubacterium saburreum
1	Cellular_org	Eubacterium saphenum ATCC 49989
1	Cellular_org	Eubacterium siraeum DSM 15702
1	Cellular_org	Eubacterium ventriosum
1	Cellular_org	Eubacterium ventriosum ATCC 27560
1	Cellular_org	Eubacterium yurii subsp. margaretiae ATCC 43715

1	Cellular_org	Exiguobacterium arabatum
1	Cellular_org	Exiguobacterium sibiricum
1	Cellular_org	Exiguobacterium sibiricum 255-15
1	Cellular_org	Exiguobacterium sp. AT1b
1	Cellular_org	Faecalibacterium cf. prausnitzii KLE1255
1	Cellular_org	Faecalibacterium prausnitzii
1	Cellular_org	Faecalibacterium prausnitzii A2-165
1	Cellular_org	Faecalibacterium prausnitzii M21/2
1	Cellular_org	Ferrimonas balearica
1	Cellular_org	Ferrimonas balearica DSM 9799
1	Cellular_org	Fervidobacterium nodosum Rt17-B1
1	Cellular_org	Fibrobacter succinogenes
1	Cellular_org	Fibrobacter succinogenes subsp. succinogenes S85
1	Cellular_org	Finegoldia magna
1	Cellular_org	Finegoldia magna ACS-171-V-Col3
1	Cellular_org	Finegoldia magna ATCC 29328
1	Cellular_org	Finegoldia magna ATCC 53516
1	Cellular_org	Finegoldia magna BVS033A4
1	Cellular_org	Flavobacteria bacterium BAL38
1	Cellular_org	Flavobacteria bacterium BBFL7
1	Cellular_org	Flavobacteria bacterium MS024-2A
1	Cellular_org	Flavobacteria bacterium MS024-3C
1	Cellular_org	Flavobacteriaceae bacterium 3519-10
1	Cellular_org	Flavobacteriales bacterium ALC-1
1	Cellular_org	Flavobacterium johnsoniae
1	Cellular_org	Flavobacterium johnsoniae UW101
1	Cellular_org	Flavobacterium psychrophilum
1	Cellular_org	Flavobacterium psychrophilum JIP02/86
1	Cellular_org	Flavobacterium sp.
1	Cellular_org	Flavobacterium sp. KI723T1
1	Cellular_org	Fluoribacter dumoffii
1	Cellular_org	Francisella novicida
1	Cellular_org	Francisella novicida FTG
1	Cellular_org	Francisella novicida GA99-3548
1	Cellular_org	Francisella novicida GA99-3549
1	Cellular_org	Francisella novicida U112
1	Cellular_org	Francisella philomiragia
1	Cellular_org	Francisella philomiragia subsp. philomiragia ATCC 25015
1	Cellular_org	Francisella philomiragia subsp. philomiragia ATCC 25017
1	Cellular_org	Francisella tularensis
1	Cellular_org	Francisella tularensis subsp. holarctica
1	Cellular_org	Francisella tularensis subsp. holarctica 257
1	Cellular_org	Francisella tularensis subsp. holarctica FSC022

1	Cellular_org	Francisella tularensis subsp. holarctica FSC200
1	Cellular_org	Francisella tularensis subsp. holarctica FTNF002-00
1	Cellular_org	Francisella tularensis subsp. holarctica LVS
1	Cellular_org	Francisella tularensis subsp. holarctica OSU18
1	Cellular_org	Francisella tularensis subsp. holarctica URFT1
1	Cellular_org	Francisella tularensis subsp. mediasiatica
1	Cellular_org	Francisella tularensis subsp. mediasiatica FSC147
1	Cellular_org	Francisella tularensis subsp. tularensis
1	Cellular_org	Francisella tularensis subsp. tularensis FSC033
1	Cellular_org	Francisella tularensis subsp. tularensis FSC198
1	Cellular_org	Francisella tularensis subsp. tularensis MA00-2987
1	Cellular_org	Francisella tularensis subsp. tularensis SCHU S4
1	Cellular_org	Francisella tularensis subsp. tularensis WY96-3418
1	Cellular_org	Frankia alni
1	Cellular_org	Frankia alni ACN14a
1	Cellular_org	Frankia sp. CcI3
1	Cellular_org	Frankia sp. CpI1
1	Cellular_org	Frankia sp. EAN1pec
1	Cellular_org	Frankia sp. EUN1f
1	Cellular_org	Frankia sp. EuI1c
1	Cellular_org	Frankia symbiont of Datisca glomerata
1	Cellular_org	Fulvimarina pelagi HTCC2506
1	Cellular_org	Fusobacterium gonidiaformans ATCC 25563
1	Cellular_org	Fusobacterium mortiferum
1	Cellular_org	Fusobacterium mortiferum ATCC 9817
1	Cellular_org	Fusobacterium nucleatum
1	Cellular_org	Fusobacterium nucleatum subsp. nucleatum
1	Cellular_org	Fusobacterium nucleatum subsp. nucleatum ATCC 23726
1	Cellular_org	Fusobacterium nucleatum subsp. nucleatum ATCC 25586
1	Cellular_org	Fusobacterium nucleatum subsp. polymorphum ATCC 10953
1	Cellular_org	Fusobacterium nucleatum subsp. vincentii ATCC 49256
1	Cellular_org	Fusobacterium periodonticum
1	Cellular_org	Fusobacterium periodonticum ATCC 33693
1	Cellular_org	Fusobacterium sp. 1_1_41FAA
1	Cellular_org	Fusobacterium sp. 2_1_31
1	Cellular_org	Fusobacterium sp. 3_1_27
1	Cellular_org	Fusobacterium sp. 3_1_33
1	Cellular_org	Fusobacterium sp. 3_1_36A2
1	Cellular_org	Fusobacterium sp. 3_1_5R
1	Cellular_org	Fusobacterium sp. 4_1_13
1	Cellular_org	Fusobacterium sp. 7_1
1	Cellular_org	Fusobacterium sp. D11

1	Cellular_org	Fusobacterium sp. D12
1	Cellular_org	Fusobacterium ulcerans ATCC 49185
1	Cellular_org	Fusobacterium varium
1	Cellular_org	Fusobacterium varium ATCC 27725
1	Cellular_org	Gallionella capsiferriformans ES-2
1	Cellular_org	Gardnerella vaginalis
1	Cellular_org	Gardnerella vaginalis 409-05
1	Cellular_org	Gardnerella vaginalis 5-1
1	Cellular_org	Gardnerella vaginalis AMD
1	Cellular_org	Gardnerella vaginalis ATCC 14018
1	Cellular_org	Gardnerella vaginalis ATCC 14019
1	Cellular_org	Gemella haemolysans
1	Cellular_org	Gemella haemolysans ATCC 10379
1	Cellular_org	Gemella morbillorum
1	Cellular_org	Gemmata obscuriglobus UQM 2246
1	Cellular_org	Gemmatimonas aurantiaca T-27
1	Cellular_org	Geobacillus kaustophilus
1	Cellular_org	Geobacillus kaustophilus HTA426
1	Cellular_org	Geobacillus sp. C56-T3
1	Cellular_org	Geobacillus sp. G11MC16
1	Cellular_org	Geobacillus sp. WCH70
1	Cellular_org	Geobacillus sp. Y4.1MC1
1	Cellular_org	Geobacillus sp. Y412MC52
1	Cellular_org	Geobacillus sp. Y412MC61
1	Cellular_org	Geobacillus stearothermophilus
1	Cellular_org	Geobacillus thermodenitrificans
1	Cellular_org	Geobacillus thermodenitrificans NG80-2
1	Cellular_org	Geobacillus thermoglucosidasius
1	Cellular_org	Geobacillus thermoglucosidasius C56-YS93
1	Cellular_org	Geobacter bemidjiensis
1	Cellular_org	Geobacter bemidjiensis Bem
1	Cellular_org	Geobacter lovleyi SZ
1	Cellular_org	Geobacter metallireducens
1	Cellular_org	Geobacter metallireducens GS-15
1	Cellular_org	Geobacter sp. FRC-32
1	Cellular_org	Geobacter sp. M18
1	Cellular_org	Geobacter sp. M21
1	Cellular_org	Geobacter sulfurreducens
1	Cellular_org	Geobacter sulfurreducens PCA
1	Cellular_org	Geobacter uraniireducens Rf4
1	Cellular_org	Geodermatophilus obscurus
1	Cellular_org	Geodermatophilus obscurus DSM 43160
1	Cellular_org	Glaciecola sp. HTCC2999

1	Cellular_org	Gloeobacter violaceus
1	Cellular_org	Gloeobacter violaceus PCC 7421
1	Cellular_org	Gluconacetobacter diazotrophicus
1	Cellular_org	Gluconacetobacter diazotrophicus PAl 5
1	Cellular_org	Gluconacetobacter hansenii
1	Cellular_org	Gluconacetobacter hansenii ATCC 23769
1	Cellular_org	Gluconacetobacter xylinus
1	Cellular_org	Gluconacetobacter xylinus NBRC 3288
1	Cellular_org	Gluconobacter oxydans
1	Cellular_org	Gluconobacter oxydans 621H
1	Cellular_org	Gordonia bronchialis
1	Cellular_org	Gordonia bronchialis DSM 43247
1	Cellular_org	Gordonia westfalica
1	Cellular_org	Gramella forsetii KT0803
1	Cellular_org	Granulibacter bethesdensis
1	Cellular_org	Granulibacter bethesdensis CGDNIH1
1	Cellular_org	Granulicatella adiacens
1	Cellular_org	Granulicatella adiacens ATCC 49175
1	Cellular_org	Granulicatella elegans
1	Cellular_org	Granulicatella elegans ATCC 700633
1	Cellular_org	Grimontia hollisae
1	Cellular_org	Grimontia hollisae CIP 101886
1	Cellular_org	Haemophilus ducreyi
1	Cellular_org	Haemophilus ducreyi 35000HP
1	Cellular_org	Haemophilus influenzae
1	Cellular_org	Haemophilus influenzae 22.1-21
1	Cellular_org	Haemophilus influenzae 22.4-21
1	Cellular_org	Haemophilus influenzae 3655
1	Cellular_org	Haemophilus influenzae 6P18H1
1	Cellular_org	Haemophilus influenzae 7P49H1
1	Cellular_org	Haemophilus influenzae 86-028NP
1	Cellular_org	Haemophilus influenzae HK1212
1	Cellular_org	Haemophilus influenzae NT127
1	Cellular_org	Haemophilus influenzae PittAA
1	Cellular_org	Haemophilus influenzae PittEE
1	Cellular_org	Haemophilus influenzae PittGG
1	Cellular_org	Haemophilus influenzae PittHH
1	Cellular_org	Haemophilus influenzae PittII
1	Cellular_org	Haemophilus influenzae R3021
1	Cellular_org	Haemophilus influenzae Rd KW20
1	Cellular_org	Haemophilus influenzae RdAW
1	Cellular_org	Haemophilus influenzae biotype aegyptius
1	Cellular_org	Haemophilus parasuis

1	Cellular_org	Haemophilus parasuis 29755
1	Cellular_org	Haemophilus parasuis SH0165
1	Cellular_org	Haemophilus somnus 129PT
1	Cellular_org	Haemophilus somnus 2336
1	Cellular_org	Hafnia alvei
1	Cellular_org	Hahella chejuensis KCTC 2396
1	Cellular_org	Haliangium ochraceum DSM 14365
1	Cellular_org	Halomonas elongata
1	Cellular_org	Halomonas elongata DSM 2581
1	Cellular_org	Halorhodospira halophila
1	Cellular_org	Halorhodospira halophila SL1
1	Cellular_org	Halothermothrix orenii
1	Cellular_org	Halothermothrix orenii H 168
1	Cellular_org	Halothiobacillus neapolitanus
1	Cellular_org	Halothiobacillus neapolitanus c2
1	Cellular_org	Helicobacter acinonychis
1	Cellular_org	Helicobacter acinonychis str. Sheeba
1	Cellular_org	Helicobacter bilis
1	Cellular_org	Helicobacter bilis ATCC 43879
1	Cellular_org	Helicobacter canadensis
1	Cellular_org	Helicobacter canadensis MIT 98-5491
1	Cellular_org	Helicobacter cinaedi
1	Cellular_org	Helicobacter cinaedi CCUG 18818
1	Cellular_org	Helicobacter felis
1	Cellular_org	Helicobacter hepaticus
1	Cellular_org	Helicobacter hepaticus ATCC 51449
1	Cellular_org	Helicobacter mustelae
1	Cellular_org	Helicobacter mustelae 12198
1	Cellular_org	Helicobacter pullorum
1	Cellular_org	Helicobacter pullorum MIT 98-5489
1	Cellular_org	Helicobacter pylori
1	Cellular_org	Helicobacter pylori 26695
1	Cellular_org	Helicobacter pylori 98-10
1	Cellular_org	Helicobacter pylori B128
1	Cellular_org	Helicobacter pylori B38
1	Cellular_org	Helicobacter pylori B8
1	Cellular_org	Helicobacter pylori G27
1	Cellular_org	Helicobacter pylori HPAG1
1	Cellular_org	Helicobacter pylori HPKX_438_AG0C1
1	Cellular_org	Helicobacter pylori HPKX_438_CA4C1
1	Cellular_org	Helicobacter pylori J99
1	Cellular_org	Helicobacter pylori P12
1	Cellular_org	Helicobacter pylori PeCan4

1	Cellular_org	Helicobacter pylori SJM180
1	Cellular_org	Helicobacter pylori Shi470
1	Cellular_org	Helicobacter suis
1	Cellular_org	Helicobacter suis HS1
1	Cellular_org	Helicobacter winghamensis
1	Cellular_org	Helicobacter winghamensis ATCC BAA-430
1	Cellular_org	Heliobacterium modesticaldum
1	Cellular_org	Heliobacterium modesticaldum Ice1
1	Cellular_org	Herbaspirillum seropedicae
1	Cellular_org	Herbaspirillum seropedicae SmR1
1	Cellular_org	Herminiimonas arsenicoxydans
1	Cellular_org	Herpetosiphon aurantiacus
1	Cellular_org	Herpetosiphon aurantiacus ATCC 23779
1	Cellular_org	Hirschia baltica ATCC 49814
1	Cellular_org	Histophilus somni
1	Cellular_org	Hoeflea phototrophica DFL-43
1	Cellular_org	Holdemania filiformis DSM 12042
1	Cellular_org	Hydrogenivirga sp. 128-5-R1-1
1	Cellular_org	Hydrogenobacter thermophilus
1	Cellular_org	Hydrogenobacter thermophilus TK-6
1	Cellular_org	Hydrogenobaculum sp. Y04AAS1
1	Cellular_org	Hyphomicrobium denitrificans
1	Cellular_org	Hyphomicrobium denitrificans ATCC 51888
1	Cellular_org	Hyphomonas neptunium
1	Cellular_org	Hyphomonas neptunium ATCC 15444
1	Cellular_org	Idiomarina baltica
1	Cellular_org	Idiomarina baltica OS145
1	Cellular_org	Idiomarina loihiensis L2TR
1	Cellular_org	Ilyobacter polytropus DSM 2926
1	Cellular_org	Isosphaera pallida ATCC 43644
1	Cellular_org	Janibacter sp. HTCC2649
1	Cellular_org	Jannaschia sp. CCS1
1	Cellular_org	Janthinobacterium sp. Marseille
1	Cellular_org	Jonesia denitrificans DSM 20603
1	Cellular_org	Jonquetella anthropi E3_33 E1
1	Cellular_org	Kangiella koreensis DSM 16069
1	Cellular_org	Ketogulonicigenium vulgare
1	Cellular_org	Ketogulonicigenium vulgare Y25
1	Cellular_org	Kineococcus radiotolerans SRS30216
1	Cellular_org	Kingella oralis ATCC 51147
1	Cellular_org	Klebsiella oxytoca
1	Cellular_org	Klebsiella oxytoca KOX105
1	Cellular_org	Klebsiella pneumoniae

1	Cellular_org	Klebsiella pneumoniae 342
1	Cellular_org	Klebsiella pneumoniae NTUH-K2044
1	Cellular_org	Klebsiella pneumoniae subsp. pneumoniae
1	Cellular_org	Klebsiella pneumoniae subsp. pneumoniae MGH 78578
1	Cellular_org	Klebsiella pneumoniae subsp. rhinoscleromatis
1	Cellular_org	Klebsiella pneumoniae subsp. rhinoscleromatis ATCC 13884
1	Cellular_org	Klebsiella sp. 1_1_55
1	Cellular_org	Klebsiella sp. KCL-2
1	Cellular_org	Klebsiella variicola
1	Cellular_org	Klebsiella variicola At-22
1	Cellular_org	Kocuria rhizophila
1	Cellular_org	Kocuria rhizophila DC2201
1	Cellular_org	Kordia algicida OT-1
1	Cellular_org	Kosmotoga olearia TBF 19.5.1
1	Cellular_org	Kribbella flavida
1	Cellular_org	Kribbella flavida DSM 17836
1	Cellular_org	Ktedonobacter racemifer DSM 44963
1	Cellular_org	Kytococcus sedentarius
1	Cellular_org	Kytococcus sedentarius DSM 20547
1	Cellular_org	Labrenzia aggregata
1	Cellular_org	Labrenzia alexandrii DFL-11
1	Cellular_org	Lactobacillus acidipiscis
1	Cellular_org	Lactobacillus acidophilus
1	Cellular_org	Lactobacillus acidophilus ATCC 4796
1	Cellular_org	Lactobacillus acidophilus NCFM
1	Cellular_org	Lactobacillus amylolyticus
1	Cellular_org	Lactobacillus amylolyticus DSM 11664
1	Cellular_org	Lactobacillus amylovorus
1	Cellular_org	Lactobacillus antri
1	Cellular_org	Lactobacillus antri DSM 16041
1	Cellular_org	Lactobacillus brevis
1	Cellular_org	Lactobacillus brevis ATCC 367
1	Cellular_org	Lactobacillus brevis subsp. gravesensis ATCC 27305
1	Cellular_org	Lactobacillus buchneri
1	Cellular_org	Lactobacillus buchneri ATCC 11577
1	Cellular_org	Lactobacillus casei
1	Cellular_org	Lactobacillus casei ATCC 334
1	Cellular_org	Lactobacillus casei BL23
1	Cellular_org	Lactobacillus casei str. Zhang
1	Cellular_org	Lactobacillus coleohominis
1	Cellular_org	Lactobacillus coleohominis 101-4-CHN
1	Cellular_org	Lactobacillus crispatus

1	Cellular_org	Lactobacillus crispatus 125-2-CHN
1	Cellular_org	Lactobacillus crispatus 214-1
1	Cellular_org	Lactobacillus crispatus JV-V01
1	Cellular_org	Lactobacillus crispatus MV-1A-US
1	Cellular_org	Lactobacillus crispatus MV-3A-US
1	Cellular_org	Lactobacillus crispatus ST1
1	Cellular_org	Lactobacillus curvatus
1	Cellular_org	Lactobacillus delbrueckii
1	Cellular_org	Lactobacillus delbrueckii subsp. bulgaricus
1	Cellular_org	Lactobacillus delbrueckii subsp. bulgaricus ATCC 11842
1	Cellular_org	Lactobacillus delbrueckii subsp. bulgaricus ATCC BAA-365
1	Cellular_org	Lactobacillus delbrueckii subsp. bulgaricus PB2003/044-T3-4
1	Cellular_org	Lactobacillus delbrueckii subsp. lactis
1	Cellular_org	Lactobacillus farciminis
1	Cellular_org	Lactobacillus fermentum
1	Cellular_org	Lactobacillus fermentum 28-3-CHN
1	Cellular_org	Lactobacillus fermentum ATCC 14931
1	Cellular_org	Lactobacillus fermentum IFO 3956
1	Cellular_org	Lactobacillus gasseri
1	Cellular_org	Lactobacillus gasseri 202-4
1	Cellular_org	Lactobacillus gasseri 224-1
1	Cellular_org	Lactobacillus gasseri ATCC 33323
1	Cellular_org	Lactobacillus gasseri JV-V03
1	Cellular_org	Lactobacillus gasseri MV-22
1	Cellular_org	Lactobacillus helveticus
1	Cellular_org	Lactobacillus helveticus DPC 4571
1	Cellular_org	Lactobacillus helveticus DSM 20075
1	Cellular_org	Lactobacillus helveticus subsp. jugurti
1	Cellular_org	Lactobacillus hilgardii
1	Cellular_org	Lactobacillus hilgardii ATCC 8290
1	Cellular_org	Lactobacillus iners
1	Cellular_org	Lactobacillus iners AB-1
1	Cellular_org	Lactobacillus iners DSM 13335
1	Cellular_org	Lactobacillus iners LactinV 01V1-a
1	Cellular_org	Lactobacillus iners LactinV 03V1-b
1	Cellular_org	Lactobacillus iners LactinV 09V1-c
1	Cellular_org	Lactobacillus iners LactinV 11V1-d
1	Cellular_org	Lactobacillus iners SPIN 2503V10-D
1	Cellular_org	Lactobacillus jensenii
1	Cellular_org	Lactobacillus jensenii 115-3-CHN
1	Cellular_org	Lactobacillus jensenii 1153
1	Cellular_org	Lactobacillus jensenii 208-1

1	Cellular_org	Lactobacillus jensenii 269-3
1	Cellular_org	Lactobacillus jensenii 27-2-CHN
1	Cellular_org	Lactobacillus jensenii JV-V16
1	Cellular_org	Lactobacillus jensenii SJ-7A-US
1	Cellular_org	Lactobacillus johnsonii
1	Cellular_org	Lactobacillus johnsonii ATCC 33200
1	Cellular_org	Lactobacillus johnsonii FI9785
1	Cellular_org	Lactobacillus johnsonii NCC 533
1	Cellular_org	Lactobacillus oris
1	Cellular_org	Lactobacillus paracasei
1	Cellular_org	Lactobacillus paracasei TXW
1	Cellular_org	Lactobacillus paracasei subsp. paracasei
1	Cellular_org	Lactobacillus paracasei subsp. paracasei 8700:2
1	Cellular_org	Lactobacillus paracasei subsp. paracasei ATCC 25302
1	Cellular_org	Lactobacillus pentosus
1	Cellular_org	Lactobacillus plantarum
1	Cellular_org	Lactobacillus plantarum JDM1
1	Cellular_org	Lactobacillus plantarum WCFS1
1	Cellular_org	Lactobacillus plantarum subsp. plantarum
1	Cellular_org	Lactobacillus plantarum subsp. plantarum ATCC 14917
1	Cellular_org	Lactobacillus plantarum subsp. plantarum ST-III
1	Cellular_org	Lactobacillus reuteri
1	Cellular_org	Lactobacillus reuteri 100-23
1	Cellular_org	Lactobacillus reuteri CF48-3A
1	Cellular_org	Lactobacillus reuteri DSM 20016
1	Cellular_org	Lactobacillus reuteri JCM 1112
1	Cellular_org	Lactobacillus reuteri MM2-3
1	Cellular_org	Lactobacillus reuteri MM4-1A
1	Cellular_org	Lactobacillus reuteri SD2112
1	Cellular_org	Lactobacillus rhamnosus
1	Cellular_org	Lactobacillus rhamnosus GG
1	Cellular_org	Lactobacillus rhamnosus HN001
1	Cellular_org	Lactobacillus rhamnosus LMS2-1
1	Cellular_org	Lactobacillus rhamnosus Lc 705
1	Cellular_org	Lactobacillus ruminis
1	Cellular_org	Lactobacillus ruminis ATCC 25644
1	Cellular_org	Lactobacillus sakei
1	Cellular_org	Lactobacillus sakei subsp. sakei
1	Cellular_org	Lactobacillus sakei subsp. sakei 23K
1	Cellular_org	Lactobacillus salivarius
1	Cellular_org	Lactobacillus salivarius ACS-116-V-Col5a
1	Cellular_org	Lactobacillus salivarius ATCC 11741
1	Cellular_org	Lactobacillus salivarius UCC118

1	Cellular_org	Lactobacillus ultunensis
1	Cellular_org	Lactobacillus ultunensis DSM 16047
1	Cellular_org	Lactobacillus vaginalis
1	Cellular_org	Lactobacillus vaginalis ATCC 49540
1	Cellular_org	Lactococcus garvieae
1	Cellular_org	Lactococcus lactis
1	Cellular_org	Lactococcus lactis subsp. cremoris
1	Cellular_org	Lactococcus lactis subsp. cremoris MG1363
1	Cellular_org	Lactococcus lactis subsp. cremoris SK11
1	Cellular_org	Lactococcus lactis subsp. lactis
1	Cellular_org	Lactococcus lactis subsp. lactis Il1403
1	Cellular_org	Lactococcus lactis subsp. lactis K214
1	Cellular_org	Lactococcus lactis subsp. lactis KF147
1	Cellular_org	Lactococcus lactis subsp. lactis bv. diacetylactis
1	Cellular_org	Laribacter hongkongensis
1	Cellular_org	Laribacter hongkongensis HLHK9
1	Cellular_org	Lawsonia intracellularis
1	Cellular_org	Lawsonia intracellularis PHE/MN1-00
1	Cellular_org	Leeuwenhoekiella blandensis MED217
1	Cellular_org	Legionella drancourtii
1	Cellular_org	Legionella drancourtii LLAP12
1	Cellular_org	Legionella longbeachae
1	Cellular_org	Legionella longbeachae D-4968
1	Cellular_org	Legionella longbeachae NSW150
1	Cellular_org	Legionella pneumophila
1	Cellular_org	Legionella pneumophila 2300/99 Alcoy
1	Cellular_org	Legionella pneumophila serogroup 1
1	Cellular_org	Legionella pneumophila str. Corby
1	Cellular_org	Legionella pneumophila str. Lens
1	Cellular_org	Legionella pneumophila str. Paris
1	Cellular_org	Legionella pneumophila subsp. pneumophila
1	Cellular_org	Legionella pneumophila subsp. pneumophila str. Philadelphia 1
1	Cellular_org	Leifsonia xyli
1	Cellular_org	Leifsonia xyli subsp. xyli
1	Cellular_org	Leifsonia xyli subsp. xyli str. CTCB07
1	Cellular_org	Lentisphaera araneosa HTCC2155
1	Cellular_org	Leptolyngbya boryana
1	Cellular_org	Leptolyngbya foveolarum
1	Cellular_org	Leptolyngbya sp. PCC 6402
1	Cellular_org	Leptolyngbya valderiana BDU 20041
1	Cellular_org	Leptospira biflexa
1	Cellular_org	Leptospira biflexa serovar Patoc

1	Cellular_org	Leptospira biflexa serovar Patoc strain 'Patoc 1 (Ames)'
1	Cellular_org	Leptospira biflexa serovar Patoc strain 'Patoc 1 (Paris)'
1	Cellular_org	Leptospira borgpetersenii
1	Cellular_org	Leptospira borgpetersenii serovar Hardjo-bovis
1	Cellular_org	Leptospira borgpetersenii serovar Hardjo-bovis JB197
1	Cellular_org	Leptospira borgpetersenii serovar Hardjo-bovis L550
1	Cellular_org	Leptospira interrogans
1	Cellular_org	Leptospira interrogans serovar Copenhageni
1	Cellular_org	Leptospira interrogans serovar Copenhageni str. Fiocruz L1-130
1	Cellular_org	Leptospira interrogans serovar Lai
1	Cellular_org	Leptospira interrogans serovar Lai str. 56601
1	Cellular_org	Leptospirillum ferrooxidans
1	Cellular_org	Leptothrix cholodnii
1	Cellular_org	Leptothrix cholodnii SP-6
1	Cellular_org	Leptotrichia buccalis
1	Cellular_org	Leptotrichia buccalis C-1013-b
1	Cellular_org	Leptotrichia goodfellowii F0264
1	Cellular_org	Leptotrichia hofstadii F0254
1	Cellular_org	Leuconostoc citreum
1	Cellular_org	Leuconostoc citreum KM20
1	Cellular_org	Leuconostoc gasicomitatum
1	Cellular_org	Leuconostoc gasicomitatum LMG 18811
1	Cellular_org	Leuconostoc kimchii
1	Cellular_org	Leuconostoc kimchii IMSNU 11154
1	Cellular_org	Leuconostoc mesenteroides
1	Cellular_org	Leuconostoc mesenteroides subsp. cremoris
1	Cellular_org	Leuconostoc mesenteroides subsp. cremoris ATCC 19254
1	Cellular_org	Leuconostoc mesenteroides subsp. mesenteroides
1	Cellular_org	Leuconostoc mesenteroides subsp. mesenteroides ATCC 8293
1	Cellular_org	Leuconostoc mesenteroides subsp. mesenteroides Y110
1	Cellular_org	Limnobacter sp. MED105
1	Cellular_org	Listeria grayi
1	Cellular_org	Listeria grayi DSM 20601
1	Cellular_org	Listeria innocua
1	Cellular_org	Listeria innocua Clip11262
1	Cellular_org	Listeria ivanovii
1	Cellular_org	Listeria marthii
1	Cellular_org	Listeria monocytogenes
1	Cellular_org	Listeria monocytogenes 08-5578
1	Cellular_org	Listeria monocytogenes 08-5923
1	Cellular_org	Listeria monocytogenes 10403S

1	Cellular_org	Listeria monocytogenes EGD-e
1	Cellular_org	Listeria monocytogenes F6900
1	Cellular_org	Listeria monocytogenes FSL F2-515
1	Cellular_org	Listeria monocytogenes FSL J1-175
1	Cellular_org	Listeria monocytogenes FSL J1-194
1	Cellular_org	Listeria monocytogenes FSL J1-208
1	Cellular_org	Listeria monocytogenes FSL J2-003
1	Cellular_org	Listeria monocytogenes FSL J2-064
1	Cellular_org	Listeria monocytogenes FSL J2-071
1	Cellular_org	Listeria monocytogenes FSL N1-017
1	Cellular_org	Listeria monocytogenes FSL N3-165
1	Cellular_org	Listeria monocytogenes FSL R2-503
1	Cellular_org	Listeria monocytogenes FSL R2-561
1	Cellular_org	Listeria monocytogenes Finland 1988
1	Cellular_org	Listeria monocytogenes HCC23
1	Cellular_org	Listeria monocytogenes HPB2262
1	Cellular_org	Listeria monocytogenes J0161
1	Cellular_org	Listeria monocytogenes J2818
1	Cellular_org	Listeria monocytogenes LO28
1	Cellular_org	Listeria monocytogenes serotype 4b str. F2365
1	Cellular_org	Listeria seeligeri
1	Cellular_org	Listeria seeligeri serovar 1/2b str. SLCC3954
1	Cellular_org	Listeria welshimeri
1	Cellular_org	Listeria welshimeri serovar 6b str. SLCC5334
1	Cellular_org	Listonella anguillarum
1	Cellular_org	Listonella anguillarum 775
1	Cellular_org	Listonella anguillarum serovar O2
1	Cellular_org	Loktanella vestfoldensis
1	Cellular_org	Loktanella vestfoldensis SKA53
1	Cellular_org	Lutiella nitroferrum 2002
1	Cellular_org	Lyngbya sp. PCC 8106
1	Cellular_org	Lysinibacillus fusiformis
1	Cellular_org	Lysinibacillus fusiformis ZC1
1	Cellular_org	Lysinibacillus sphaericus
1	Cellular_org	Lysinibacillus sphaericus C3-41
1	Cellular_org	Macrococcus caseolyticus
1	Cellular_org	Macrococcus caseolyticus JCSC5402
1	Cellular_org	Magnetococcus sp. MC-1
1	Cellular_org	Magnetospirillum gryphiswaldense
1	Cellular_org	Magnetospirillum gryphiswaldense MSR-1
1	Cellular_org	Magnetospirillum magneticum
1	Cellular_org	Magnetospirillum magneticum AMB-1
1	Cellular_org	Magnetospirillum magnetotacticum

1	Cellular_org	Magnetospirillum magnetotacticum MS-1
1	Cellular_org	Mannheimia haemolytica
1	Cellular_org	Mannheimia haemolytica PHL213
1	Cellular_org	Mannheimia haemolytica serotype A2 str. BOVINE
1	Cellular_org	Mannheimia haemolytica serotype A2 str. OVINE
1	Cellular_org	Mannheimia succiniciproducens MBEL55E
1	Cellular_org	Mannheimia varigena
1	Cellular_org	Maricaulis maris
1	Cellular_org	Maricaulis maris MCS10
1	Cellular_org	Marinobacter algicola DG893
1	Cellular_org	Marinobacter aquaeolei VT8
1	Cellular_org	Marinobacter hydrocarbonoclasticus
1	Cellular_org	Marinobacter sp. ELB17
1	Cellular_org	Marinococcus halophilus
1	Cellular_org	Marinomonas sp. MED121
1	Cellular_org	Marinomonas sp. MWYL1
1	Cellular_org	Mariprofundus ferrooxydans PV-1
1	Cellular_org	Maritimibacter alkaliphilus HTCC2654
1	Cellular_org	Megasphaera genomosp. type_1 str. 28L
1	Cellular_org	Megasphaera micronuciformis
1	Cellular_org	Megasphaera micronuciformis F0359
1	Cellular_org	Meiothermus ruber
1	Cellular_org	Meiothermus ruber DSM 1279
1	Cellular_org	Meiothermus silvanus DSM 9946
1	Cellular_org	Mesoplasma florum
1	Cellular_org	Mesoplasma florum L1
1	Cellular_org	Mesorhizobium ciceri
1	Cellular_org	Mesorhizobium ciceri biovar biserrulae
1	Cellular_org	Mesorhizobium loti
1	Cellular_org	Mesorhizobium loti MAFF303099
1	Cellular_org	Mesorhizobium opportunistum
1	Cellular_org	Mesorhizobium opportunistum WSM2075
1	Cellular_org	Methylacidiphilum infernorum V4
1	Cellular_org	Methylibium petroleiphilum PM1
1	Cellular_org	Methylobacillus flagellatus
1	Cellular_org	Methylobacillus flagellatus KT
1	Cellular_org	Methylobacter tundripaludum
1	Cellular_org	Methylobacter tundripaludum SV96
1	Cellular_org	Methylobacterium chloromethanicum
1	Cellular_org	Methylobacterium chloromethanicum CM4
1	Cellular_org	Methylobacterium extorquens
1	Cellular_org	Methylobacterium extorquens AM1
1	Cellular_org	Methylobacterium extorquens DM4

1	Cellular_org	Methylobacterium extorquens PA1
1	Cellular_org	Methylobacterium nodulans
1	Cellular_org	Methylobacterium nodulans ORS 2060
1	Cellular_org	Methylobacterium populi BJ001
1	Cellular_org	Methylobacterium radiotolerans
1	Cellular_org	Methylobacterium radiotolerans JCM 2831
1	Cellular_org	Methylobacterium sp. 4-46
1	Cellular_org	Methylocella silvestris
1	Cellular_org	Methylocella silvestris BL2
1	Cellular_org	Methylococcus capsulatus
1	Cellular_org	Methylococcus capsulatus str. Bath
1	Cellular_org	Methylocystis sp. ATCC 49242
1	Cellular_org	Methylophaga thalassica
1	Cellular_org	Methylophilales bacterium HTCC2181
1	Cellular_org	Methylosinus trichosporium
1	Cellular_org	Methylosinus trichosporium OB3b
1	Cellular_org	Methylotenera mobilis JLW8
1	Cellular_org	Methylotenera sp. 301
1	Cellular_org	Methylovorus sp. SIP3-4
1	Cellular_org	Microbacterium testaceum
1	Cellular_org	Micrococcus luteus
1	Cellular_org	Micrococcus luteus NCTC 2665
1	Cellular_org	Micrococcus luteus SK58
1	Cellular_org	Micrococcus sp. 28
1	Cellular_org	Microcoleus chthonoplastes
1	Cellular_org	Microcoleus chthonoplastes PCC 7420
1	Cellular_org	Microcystis
1	Cellular_org	Microcystis aeruginosa
1	Cellular_org	Microcystis aeruginosa NIES-843
1	Cellular_org	Micromonospora aurantiaca
1	Cellular_org	Micromonospora aurantiaca ATCC 27029
1	Cellular_org	Micromonospora rosaria
1	Cellular_org	Micromonospora sp. ATCC 39149
1	Cellular_org	Micromonospora sp. L5
1	Cellular_org	Microscilla marina
1	Cellular_org	Microscilla marina ATCC 23134
1	Cellular_org	Microscilla sp. PRE1
1	Cellular_org	Mitsuokella multacida
1	Cellular_org	Mitsuokella multacida DSM 20544
1	Cellular_org	Mobiluncus curtisii
1	Cellular_org	Mobiluncus curtisii ATCC 43063
1	Cellular_org	Mobiluncus curtisii subsp. curtisii
1	Cellular_org	Mobiluncus curtisii subsp. curtisii ATCC 35241

1	Cellular_org	Mobiluncus curtisii subsp. holmesii
1	Cellular_org	Mobiluncus mulieris 28-1
1	Cellular_org	Mobiluncus mulieris ATCC 35239
1	Cellular_org	Mobiluncus mulieris ATCC 35243
1	Cellular_org	Mobiluncus mulieris FB024-16
1	Cellular_org	Moorella thermoacetica
1	Cellular_org	Moorella thermoacetica ATCC 39073
1	Cellular_org	Moraxella bovis
1	Cellular_org	Moraxella bovis Epp63
1	Cellular_org	Moraxella catarrhalis
1	Cellular_org	Moraxella catarrhalis RH4
1	Cellular_org	Moraxella sp. TA144
1	Cellular_org	Moritella sp. PE36
1	Cellular_org	Mycobacterium abscessus
1	Cellular_org	Mycobacterium abscessus ATCC 19977
1	Cellular_org	Mycobacterium avium
1	Cellular_org	Mycobacterium avium 104
1	Cellular_org	Mycobacterium avium subsp. avium
1	Cellular_org	Mycobacterium avium subsp. avium ATCC 25291
1	Cellular_org	Mycobacterium avium subsp. paratuberculosis
1	Cellular_org	Mycobacterium avium subsp. paratuberculosis K-10
1	Cellular_org	Mycobacterium bovis
1	Cellular_org	Mycobacterium bovis AF2122/97
1	Cellular_org	Mycobacterium bovis BCG
1	Cellular_org	Mycobacterium bovis BCG str. Pasteur 1173P2
1	Cellular_org	Mycobacterium bovis BCG str. Tokyo 172
1	Cellular_org	Mycobacterium celatum
1	Cellular_org	Mycobacterium gilvum
1	Cellular_org	Mycobacterium gilvum PYR-GCK
1	Cellular_org	Mycobacterium intracellulare
1	Cellular_org	Mycobacterium intracellulare ATCC 13950
1	Cellular_org	Mycobacterium kansasii
1	Cellular_org	Mycobacterium kansasii ATCC 12478
1	Cellular_org	Mycobacterium leprae
1	Cellular_org	Mycobacterium leprae Br4923
1	Cellular_org	Mycobacterium leprae TN
1	Cellular_org	Mycobacterium liflandii
1	Cellular_org	Mycobacterium liflandii 128FXT
1	Cellular_org	Mycobacterium marinum
1	Cellular_org	Mycobacterium marinum M
1	Cellular_org	Mycobacterium parascrofulaceum
1	Cellular_org	Mycobacterium parascrofulaceum ATCC BAA-614
1	Cellular_org	Mycobacterium smegmatis

1	Cellular_org	Mycobacterium smegmatis str. MC2 155
1	Cellular_org	Mycobacterium sp. JLS
1	Cellular_org	Mycobacterium sp. KMS
1	Cellular_org	Mycobacterium sp. MCS
1	Cellular_org	Mycobacterium sp. Spyr1
1	Cellular_org	Mycobacterium tuberculosis
1	Cellular_org	Mycobacterium tuberculosis '98-R604 INH-RIF-EM'
1	Cellular_org	Mycobacterium tuberculosis 02_1987
1	Cellular_org	Mycobacterium tuberculosis 210
1	Cellular_org	Mycobacterium tuberculosis 94_M4241A
1	Cellular_org	Mycobacterium tuberculosis C
1	Cellular_org	Mycobacterium tuberculosis CDC1551
1	Cellular_org	Mycobacterium tuberculosis CPHL_A
1	Cellular_org	Mycobacterium tuberculosis EAS054
1	Cellular_org	Mycobacterium tuberculosis F11
1	Cellular_org	Mycobacterium tuberculosis GM 1503
1	Cellular_org	Mycobacterium tuberculosis H37Ra
1	Cellular_org	Mycobacterium tuberculosis H37Rv
1	Cellular_org	Mycobacterium tuberculosis K85
1	Cellular_org	Mycobacterium tuberculosis KZN 1435
1	Cellular_org	Mycobacterium tuberculosis KZN 4207
1	Cellular_org	Mycobacterium tuberculosis KZN 605
1	Cellular_org	Mycobacterium tuberculosis KZN R506
1	Cellular_org	Mycobacterium tuberculosis KZN V2475
1	Cellular_org	Mycobacterium tuberculosis SUMu001
1	Cellular_org	Mycobacterium tuberculosis SUMu002
1	Cellular_org	Mycobacterium tuberculosis SUMu003
1	Cellular_org	Mycobacterium tuberculosis SUMu004
1	Cellular_org	Mycobacterium tuberculosis SUMu005
1	Cellular_org	Mycobacterium tuberculosis SUMu006
1	Cellular_org	Mycobacterium tuberculosis SUMu007
1	Cellular_org	Mycobacterium tuberculosis SUMu008
1	Cellular_org	Mycobacterium tuberculosis SUMu009
1	Cellular_org	Mycobacterium tuberculosis SUMu010
1	Cellular_org	Mycobacterium tuberculosis SUMu011
1	Cellular_org	Mycobacterium tuberculosis SUMu012
1	Cellular_org	Mycobacterium tuberculosis T17
1	Cellular_org	Mycobacterium tuberculosis T46
1	Cellular_org	Mycobacterium tuberculosis T85
1	Cellular_org	Mycobacterium tuberculosis T92
1	Cellular_org	Mycobacterium tuberculosis str. Haarlem
1	Cellular_org	Mycobacterium ulcerans
1	Cellular_org	Mycobacterium ulcerans Agy99

1	Cellular_org	Mycobacterium vanbaalenii
1	Cellular_org	Mycobacterium vanbaalenii PYR-1
1	Cellular_org	Mycoplasma agalactiae
1	Cellular_org	Mycoplasma agalactiae PG2
1	Cellular_org	Mycoplasma alligatoris A21JP2
1	Cellular_org	Mycoplasma arthritidis
1	Cellular_org	Mycoplasma arthritidis 158L3-1
1	Cellular_org	Mycoplasma bovis
1	Cellular_org	Mycoplasma capricolum
1	Cellular_org	Mycoplasma capricolum subsp. capricolum
1	Cellular_org	Mycoplasma capricolum subsp. capricolum ATCC 27343
1	Cellular_org	Mycoplasma conjunctivae
1	Cellular_org	Mycoplasma conjunctivae HRC/581
1	Cellular_org	Mycoplasma crocodyli MP145
1	Cellular_org	Mycoplasma fermentans
1	Cellular_org	Mycoplasma fermentans JER
1	Cellular_org	Mycoplasma gallisepticum
1	Cellular_org	Mycoplasma gallisepticum str. R
1	Cellular_org	Mycoplasma gallisepticum str. R(low)
1	Cellular_org	Mycoplasma genitalium
1	Cellular_org	Mycoplasma genitalium G37
1	Cellular_org	Mycoplasma haemofelis
1	Cellular_org	Mycoplasma hominis
1	Cellular_org	Mycoplasma hyopneumoniae
1	Cellular_org	Mycoplasma hyopneumoniae 232
1	Cellular_org	Mycoplasma hyopneumoniae 7448
1	Cellular_org	Mycoplasma hyopneumoniae J
1	Cellular_org	Mycoplasma hyorhinis
1	Cellular_org	Mycoplasma hyorhinis HUB-1
1	Cellular_org	Mycoplasma leachii
1	Cellular_org	Mycoplasma mobile
1	Cellular_org	Mycoplasma mobile 163K
1	Cellular_org	Mycoplasma mycoides
1	Cellular_org	Mycoplasma mycoides subsp. mycoides
1	Cellular_org	Mycoplasma mycoides subsp. mycoides SC
1	Cellular_org	Mycoplasma mycoides subsp. mycoides SC str. PG1
1	Cellular_org	Mycoplasma penetrans
1	Cellular_org	Mycoplasma penetrans HF-2
1	Cellular_org	Mycoplasma pneumoniae
1	Cellular_org	Mycoplasma pneumoniae M129
1	Cellular_org	Mycoplasma pulmonis
1	Cellular_org	Mycoplasma pulmonis UAB CTIP
1	Cellular_org	Mycoplasma suis

1	Cellular_org	Mycoplasma synoviae
1	Cellular_org	Mycoplasma synoviae 53
1	Cellular_org	Mycoplasma yeatsii
1	Cellular_org	Myxococcus fulvus
1	Cellular_org	Myxococcus xanthus
1	Cellular_org	Myxococcus xanthus DK 1622
1	Cellular_org	Nakamurella multipartita DSM 44233
1	Cellular_org	Natranaerobius thermophilus JW/NM-WN-LF
1	Cellular_org	Nautilia profundicola AmH
1	Cellular_org	Neisseria cinerea
1	Cellular_org	Neisseria cinerea ATCC 14685
1	Cellular_org	Neisseria elongata
1	Cellular_org	Neisseria elongata subsp. glycolytica ATCC 29315
1	Cellular_org	Neisseria flavescens
1	Cellular_org	Neisseria flavescens NRL30031/H210
1	Cellular_org	Neisseria flavescens SK114
1	Cellular_org	Neisseria gonorrhoeae
1	Cellular_org	Neisseria gonorrhoeae 1291
1	Cellular_org	Neisseria gonorrhoeae 35/02
1	Cellular_org	Neisseria gonorrhoeae DGI18
1	Cellular_org	Neisseria gonorrhoeae DGI2
1	Cellular_org	Neisseria gonorrhoeae F62
1	Cellular_org	Neisseria gonorrhoeae FA 1090
1	Cellular_org	Neisseria gonorrhoeae FA19
1	Cellular_org	Neisseria gonorrhoeae FA6140
1	Cellular_org	Neisseria gonorrhoeae MS11
1	Cellular_org	Neisseria gonorrhoeae NCCP11945
1	Cellular_org	Neisseria gonorrhoeae PID1
1	Cellular_org	Neisseria gonorrhoeae PID18
1	Cellular_org	Neisseria gonorrhoeae PID24-1
1	Cellular_org	Neisseria gonorrhoeae PID332
1	Cellular_org	Neisseria gonorrhoeae SK-92-679
1	Cellular_org	Neisseria gonorrhoeae SK-93-1035
1	Cellular_org	Neisseria lactamica
1	Cellular_org	Neisseria lactamica ATCC 23970
1	Cellular_org	Neisseria meningitidis
1	Cellular_org	Neisseria meningitidis 053442
1	Cellular_org	Neisseria meningitidis ATCC 13091
1	Cellular_org	Neisseria meningitidis FAM18
1	Cellular_org	Neisseria meningitidis MC58
1	Cellular_org	Neisseria meningitidis Z2491
1	Cellular_org	Neisseria meningitidis alpha14
1	Cellular_org	Neisseria meningitidis serogroup A

1	Cellular_org	Neisseria meningitidis serogroup B
1	Cellular_org	Neisseria meningitidis serogroup C
1	Cellular_org	Neisseria mucosa
1	Cellular_org	Neisseria mucosa ATCC 25996
1	Cellular_org	Neisseria polysaccharea
1	Cellular_org	Neisseria polysaccharea ATCC 43768
1	Cellular_org	Neisseria sicca
1	Cellular_org	Neisseria sicca ATCC 29256
1	Cellular_org	Neisseria sp. oral taxon 014 str. F0314
1	Cellular_org	Neisseria subflava
1	Cellular_org	Neisseria subflava NJ9703
1	Cellular_org	Neorickettsia risticii str. Illinois
1	Cellular_org	Neorickettsia sennetsu
1	Cellular_org	Neorickettsia sennetsu str. Miyayama
1	Cellular_org	Nitratifractor salsuginis
1	Cellular_org	Nitratifractor salsuginis DSM 16511
1	Cellular_org	Nitratiruptor sp. SB155-2
1	Cellular_org	Nitrobacter hamburgensis
1	Cellular_org	Nitrobacter hamburgensis X14
1	Cellular_org	Nitrobacter sp. Nb-311A
1	Cellular_org	Nitrobacter winogradskyi
1	Cellular_org	Nitrobacter winogradskyi Nb-255
1	Cellular_org	Nitrococcus mobilis
1	Cellular_org	Nitrococcus mobilis Nb-231
1	Cellular_org	Nitrosococcus halophilus
1	Cellular_org	Nitrosococcus halophilus Nc4
1	Cellular_org	Nitrosococcus oceani
1	Cellular_org	Nitrosococcus oceani AFC27
1	Cellular_org	Nitrosococcus oceani ATCC 19707
1	Cellular_org	Nitrosococcus watsoni C-113
1	Cellular_org	Nitrosomonas europaea
1	Cellular_org	Nitrosomonas europaea ATCC 19718
1	Cellular_org	Nitrosomonas eutropha
1	Cellular_org	Nitrosomonas eutropha C91
1	Cellular_org	Nitrosomonas sp.
1	Cellular_org	Nitrosomonas sp. AL212
1	Cellular_org	Nitrosospira multiformis
1	Cellular_org	Nitrosospira multiformis ATCC 25196
1	Cellular_org	Nocardia aobensis
1	Cellular_org	Nocardia farcinica
1	Cellular_org	Nocardia farcinica IFM 10152
1	Cellular_org	Nocardia sp. 107
1	Cellular_org	Nocardia sp. C-14-1

1	Cellular_org	Nocardioides sp. JS614
1	Cellular_org	Nocardiopsis dassonvillei
1	Cellular_org	Nocardiopsis dassonvillei subsp. dassonvillei
1	Cellular_org	Nocardiopsis dassonvillei subsp. dassonvillei DSM 43111
1	Cellular_org	Nocardiopsis sp. 90127
1	Cellular_org	Nodularia spumigena
1	Cellular_org	Nodularia spumigena CCY9414
1	Cellular_org	Nostoc punctiforme
1	Cellular_org	Nostoc punctiforme PCC 73102
1	Cellular_org	Nostoc sp. PCC 7120
1	Cellular_org	Nostoc sp. PCC 7524
1	Cellular_org	Novosphingobium aromaticivorans
1	Cellular_org	Novosphingobium aromaticivorans DSM 12444
1	Cellular_org	Oceanibulbus indolifex
1	Cellular_org	Oceanibulbus indolifex HEL-45
1	Cellular_org	Oceanicola batsensis
1	Cellular_org	Oceanicola batsensis HTCC2597
1	Cellular_org	Oceanicola granulosus
1	Cellular_org	Oceanicola granulosus HTCC2516
1	Cellular_org	Oceanobacillus iheyensis
1	Cellular_org	Oceanobacillus iheyensis HTE831
1	Cellular_org	Ochrobactrum anthropi
1	Cellular_org	Ochrobactrum anthropi ATCC 49188
1	Cellular_org	Ochrobactrum intermedium
1	Cellular_org	Ochrobactrum intermedium LMG 3301
1	Cellular_org	Octadecabacter antarcticus
1	Cellular_org	Octadecabacter antarcticus 238
1	Cellular_org	Octadecabacter antarcticus 307
1	Cellular_org	Oenococcus oeni
1	Cellular_org	Oenococcus oeni ATCC BAA-1163
1	Cellular_org	Oenococcus oeni AWRIB429
1	Cellular_org	Oenococcus oeni PSU-1
1	Cellular_org	Oligotropha carboxidovorans
1	Cellular_org	Oligotropha carboxidovorans OM5
1	Cellular_org	Olsenella uli DSM 7084
1	Cellular_org	Onion yellows phytoplasma
1	Cellular_org	Onion yellows phytoplasma OY-M
1	Cellular_org	Opitutaceae bacterium TAV2
1	Cellular_org	Opitutus terrae
1	Cellular_org	Opitutus terrae PB90-1
1	Cellular_org	Oribacterium sinus F0268
1	Cellular_org	Oribacterium sp. oral taxon 078 str. F0262
1	Cellular_org	Orientia tsutsugamushi

1	Cellular_org	Orientia tsutsugamushi str. Boryong
1	Cellular_org	Orientia tsutsugamushi str. Ikeda
1	Cellular_org	Ornithobacterium rhinotracheale
1	Cellular_org	Oscillatoria sp. PCC 6506
1	Cellular_org	Oscillochloris trichoides
1	Cellular_org	Oxalobacter formigenes
1	Cellular_org	Oxalobacter formigenes HOxBLS
1	Cellular_org	Oxalobacter formigenes OXCC13
1	Cellular_org	Paenibacillus curdlanolyticus
1	Cellular_org	Paenibacillus curdlanolyticus YK9
1	Cellular_org	Paenibacillus larvae
1	Cellular_org	Paenibacillus larvae subsp. larvae
1	Cellular_org	Paenibacillus larvae subsp. larvae BRL-230010
1	Cellular_org	Paenibacillus polymyxa
1	Cellular_org	Paenibacillus polymyxa E681
1	Cellular_org	Paenibacillus polymyxa SC2
1	Cellular_org	Paenibacillus popilliae
1	Cellular_org	Paenibacillus sp. JDR-2
1	Cellular_org	Paenibacillus sp. oral taxon 786 str. D14
1	Cellular_org	Pantoea agglomerans
1	Cellular_org	Pantoea ananatis
1	Cellular_org	Pantoea ananatis LMG 20103
1	Cellular_org	Pantoea citrea
1	Cellular_org	Pantoea sp. At-9b
1	Cellular_org	Pantoea sp. aB
1	Cellular_org	Pantoea vagans
1	Cellular_org	Pantoea vagans C9-1
1	Cellular_org	Parabacteroides distasonis
1	Cellular_org	Parabacteroides distasonis ATCC 8503
1	Cellular_org	Parabacteroides johnsonii DSM 18315
1	Cellular_org	Parabacteroides merdae
1	Cellular_org	Parabacteroides merdae ATCC 43184
1	Cellular_org	Parabacteroides sp. D13
1	Cellular_org	Parachlamydia acanthamoebae
1	Cellular_org	Parachlamydia acanthamoebae str. Hall's coccus
1	Cellular_org	Paracoccus aminophilus
1	Cellular_org	Paracoccus denitrificans
1	Cellular_org	Paracoccus denitrificans PD1222
1	Cellular_org	Paracoccus methylutens
1	Cellular_org	Paracoccus pantotrophus
1	Cellular_org	Parascardovia denticolens DSM 10105
1	Cellular_org	Parascardovia denticolens F0305
1	Cellular_org	Parvibaculum lavamentivorans DS-1

1	Cellular_org	Parvimonas micra
1	Cellular_org	Parvimonas micra ATCC 33270
1	Cellular_org	Parvularcula bermudensis HTCC2503
1	Cellular_org	Pasteurella dagmatis
1	Cellular_org	Pasteurella dagmatis ATCC 43325
1	Cellular_org	Pasteurella multocida
1	Cellular_org	Pasteurella multocida subsp. multocida
1	Cellular_org	Pasteurella multocida subsp. multocida str. Pm70
1	Cellular_org	Pasteuria nishizawae str. North American
1	Cellular_org	Paulownia witches'-broom phytoplasma
1	Cellular_org	Peanut witches'-broom phytoplasma
1	Cellular_org	Pectobacterium atrosepticum
1	Cellular_org	Pectobacterium atrosepticum SCRI1043
1	Cellular_org	Pectobacterium carotovorum
1	Cellular_org	Pectobacterium carotovorum subsp. brasiliensis
1	Cellular_org	Pectobacterium carotovorum subsp. brasiliensis PBR1692
1	Cellular_org	Pectobacterium carotovorum subsp. carotovorum
1	Cellular_org	Pectobacterium carotovorum subsp. carotovorum PC1
1	Cellular_org	Pectobacterium carotovorum subsp. carotovorum WPP14
1	Cellular_org	Pectobacterium wasabiae
1	Cellular_org	Pectobacterium wasabiae WPP163
1	Cellular_org	Pediococcus acidilactici
1	Cellular_org	Pediococcus acidilactici 7_4
1	Cellular_org	Pediococcus acidilactici DSM 20284
1	Cellular_org	Pediococcus damnosus
1	Cellular_org	Pediococcus pentosaceus
1	Cellular_org	Pediococcus pentosaceus ATCC 25745
1	Cellular_org	Pedobacter heparinus
1	Cellular_org	Pedobacter heparinus DSM 2366
1	Cellular_org	Pedobacter sp. BAL39
1	Cellular_org	Pelagibaca bermudensis HTCC2601
1	Cellular_org	Pelobacter carbinolicus
1	Cellular_org	Pelobacter carbinolicus DSM 2380
1	Cellular_org	Pelobacter propionicus
1	Cellular_org	Pelobacter propionicus DSM 2379
1	Cellular_org	Pelodictyon luteolum
1	Cellular_org	Pelodictyon phaeoclathratiforme
1	Cellular_org	Pelodictyon phaeoclathratiforme BU-1
1	Cellular_org	Pelotomaculum thermopropionicum
1	Cellular_org	Pelotomaculum thermopropionicum SI
1	Cellular_org	Peptoniphilus duerdenii ATCC BAA-1640
1	Cellular_org	Peptoniphilus harei
1	Cellular_org	Peptoniphilus lacrimalis

1	Cellular_org	Peptoniphilus lacrimalis 315-B
1	Cellular_org	Peptoniphilus sp. oral taxon 386 str. F0131
1	Cellular_org	Peptoniphilus sp. oral taxon 836 str. F0141
1	Cellular_org	Peptostreptococcus anaerobius
1	Cellular_org	Peptostreptococcus anaerobius 653-L
1	Cellular_org	Peptostreptococcus stomatis DSM 17678
1	Cellular_org	Persephonella marina
1	Cellular_org	Persephonella marina EX-H1
1	Cellular_org	Petrotoga mobilis SJ95
1	Cellular_org	Phaeobacter gallaeciensis
1	Cellular_org	Phaeobacter gallaeciensis 2.10
1	Cellular_org	Phaeobacter gallaeciensis BS107
1	Cellular_org	Phenylobacterium zucineum
1	Cellular_org	Phenylobacterium zucineum HLK1
1	Cellular_org	Photobacterium angustum
1	Cellular_org	Photobacterium angustum S14
1	Cellular_org	Photobacterium damselae
1	Cellular_org	Photobacterium damselae subsp. damselae
1	Cellular_org	Photobacterium damselae subsp. damselae CIP 102761
1	Cellular_org	Photobacterium profundum
1	Cellular_org	Photobacterium profundum 3TCK
1	Cellular_org	Photobacterium profundum SS9
1	Cellular_org	Photobacterium sp. SKA34
1	Cellular_org	Photorhabdus asymbiotica
1	Cellular_org	Photorhabdus asymbiotica subsp. asymbiotica
1	Cellular_org	Photorhabdus asymbiotica subsp. asymbiotica ATCC 43949
1	Cellular_org	Photorhabdus luminescens
1	Cellular_org	Photorhabdus luminescens subsp. laumondii
1	Cellular_org	Photorhabdus luminescens subsp. laumondii TTO1
1	Cellular_org	Phytoplasma sp.
1	Cellular_org	Pirellula staleyi
1	Cellular_org	Pirellula staleyi DSM 6068
1	Cellular_org	Planctomyces brasiliensis DSM 5305
1	Cellular_org	Planctomyces limnophilus
1	Cellular_org	Planctomyces limnophilus DSM 3776
1	Cellular_org	Planctomyces maris
1	Cellular_org	Planctomyces maris DSM 8797
1	Cellular_org	Planococcus sp. ZOYM
1	Cellular_org	Plesiocystis pacifica
1	Cellular_org	Plesiocystis pacifica SIR-1
1	Cellular_org	Polaribacter irgensii 23-P
1	Cellular_org	Polaribacter sp. MED152

1	Cellular_org	Polaromonas naphthalenivorans CJ2
1	Cellular_org	Polaromonas sp. JS666
1	Cellular_org	Polynucleobacter necessarius subsp. asymbioticus
1	Cellular_org	Polynucleobacter necessarius subsp. asymbioticus QLW-P1DMWA-1
1	Cellular_org	Polynucleobacter necessarius subsp. necessarius
1	Cellular_org	Polynucleobacter necessarius subsp. necessarius STIR1
1	Cellular_org	Porphyromonas asaccharolytica
1	Cellular_org	Porphyromonas endodontalis
1	Cellular_org	Porphyromonas endodontalis ATCC 35406
1	Cellular_org	Porphyromonas gingivalis
1	Cellular_org	Porphyromonas gingivalis ATCC 33277
1	Cellular_org	Porphyromonas gingivalis W83
1	Cellular_org	Porphyromonas uenonis
1	Cellular_org	Porphyromonas uenonis 60-3
1	Cellular_org	Prevotella amnii
1	Cellular_org	Prevotella amnii CRIS 21A-A
1	Cellular_org	Prevotella bergensis
1	Cellular_org	Prevotella bergensis DSM 17361
1	Cellular_org	Prevotella bivia
1	Cellular_org	Prevotella bivia JCVIHMP010
1	Cellular_org	Prevotella bryantii
1	Cellular_org	Prevotella bryantii B14
1	Cellular_org	Prevotella buccae
1	Cellular_org	Prevotella buccae ATCC 33574
1	Cellular_org	Prevotella buccae D17
1	Cellular_org	Prevotella buccalis
1	Cellular_org	Prevotella buccalis ATCC 35310
1	Cellular_org	Prevotella copri DSM 18205
1	Cellular_org	Prevotella disiens
1	Cellular_org	Prevotella disiens FB035-09AN
1	Cellular_org	Prevotella marshii
1	Cellular_org	Prevotella marshii DSM 16973
1	Cellular_org	Prevotella melaninogenica
1	Cellular_org	Prevotella melaninogenica ATCC 25845
1	Cellular_org	Prevotella melaninogenica D18
1	Cellular_org	Prevotella oralis
1	Cellular_org	Prevotella oralis ATCC 33269
1	Cellular_org	Prevotella oris
1	Cellular_org	Prevotella oris C735
1	Cellular_org	Prevotella oris F0302
1	Cellular_org	Prevotella ruminicola
1	Cellular_org	Prevotella ruminicola 23

1	Cellular_org	Prevotella salivae DSM 15606
1	Cellular_org	Prevotella sp. oral taxon 299 str. F0039
1	Cellular_org	Prevotella sp. oral taxon 317 str. F0108
1	Cellular_org	Prevotella sp. oral taxon 472 str. F0295
1	Cellular_org	Prevotella tannerae ATCC 51259
1	Cellular_org	Prevotella timonensis
1	Cellular_org	Prevotella timonensis CRIS 5C-B1
1	Cellular_org	Prevotella veroralis
1	Cellular_org	Prevotella veroralis F0319
1	Cellular_org	Prochlorococcus marinus
1	Cellular_org	Prochlorococcus marinus str. AS9601
1	Cellular_org	Prochlorococcus marinus str. MIT 9202
1	Cellular_org	Prochlorococcus marinus str. MIT 9211
1	Cellular_org	Prochlorococcus marinus str. MIT 9215
1	Cellular_org	Prochlorococcus marinus str. MIT 9301
1	Cellular_org	Prochlorococcus marinus str. MIT 9303
1	Cellular_org	Prochlorococcus marinus str. MIT 9312
1	Cellular_org	Prochlorococcus marinus str. MIT 9313
1	Cellular_org	Prochlorococcus marinus str. MIT 9515
1	Cellular_org	Prochlorococcus marinus str. NATL1A
1	Cellular_org	Prochlorococcus marinus str. NATL2A
1	Cellular_org	Prochlorococcus marinus subsp. marinus str. CCMP1375
1	Cellular_org	Prochlorococcus marinus subsp. pastoris
1	Cellular_org	Prochlorococcus marinus subsp. pastoris str. CCMP1986
1	Cellular_org	Propionibacterium acidipropionici
1	Cellular_org	Propionibacterium acnes
1	Cellular_org	Propionibacterium acnes J139
1	Cellular_org	Propionibacterium acnes J165
1	Cellular_org	Propionibacterium acnes KPA171202
1	Cellular_org	Propionibacterium acnes SK137
1	Cellular_org	Propionibacterium acnes SK187
1	Cellular_org	Propionibacterium freudenreichii
1	Cellular_org	Propionibacterium freudenreichii subsp. shermanii
1	Cellular_org	Propionibacterium freudenreichii subsp. shermanii CIRM-BIA1
1	Cellular_org	Propionibacterium granulosum
1	Cellular_org	Propionibacterium jensenii
1	Cellular_org	Prosthecochloris aestuarii
1	Cellular_org	Prosthecochloris aestuarii DSM 271
1	Cellular_org	Proteus mirabilis
1	Cellular_org	Proteus mirabilis ATCC 29906
1	Cellular_org	Proteus mirabilis HI4320
1	Cellular_org	Proteus penneri

1	Cellular_org	Proteus penneri ATCC 35198
1	Cellular_org	Proteus vulgaris
1	Cellular_org	Providencia alcalifaciens
1	Cellular_org	Providencia alcalifaciens DSM 30120
1	Cellular_org	Providencia rettgeri
1	Cellular_org	Providencia rettgeri DSM 1131
1	Cellular_org	Providencia rustigianii
1	Cellular_org	Providencia rustigianii DSM 4541
1	Cellular_org	Providencia stuartii
1	Cellular_org	Providencia stuartii ATCC 25827
1	Cellular_org	Pseudoalteromonas atlantica
1	Cellular_org	Pseudoalteromonas atlantica T6c
1	Cellular_org	Pseudoalteromonas haloplanktis
1	Cellular_org	Pseudoalteromonas haloplanktis TAC125
1	Cellular_org	Pseudoalteromonas sp. 643A
1	Cellular_org	Pseudoalteromonas sp. BSi20327
1	Cellular_org	Pseudoalteromonas sp. PS1M3
1	Cellular_org	Pseudoalteromonas sp. SM9913
1	Cellular_org	Pseudoalteromonas tunicata
1	Cellular_org	Pseudoalteromonas tunicata D2
1	Cellular_org	Pseudomonas
1	Cellular_org	Pseudomonas aeruginosa
1	Cellular_org	Pseudomonas aeruginosa 2192
1	Cellular_org	Pseudomonas aeruginosa C3719
1	Cellular_org	Pseudomonas aeruginosa LESB58
1	Cellular_org	Pseudomonas aeruginosa PA7
1	Cellular_org	Pseudomonas aeruginosa PACS2
1	Cellular_org	Pseudomonas aeruginosa PAO1
1	Cellular_org	Pseudomonas aeruginosa PAb1
1	Cellular_org	Pseudomonas aeruginosa UCBPP-PA14
1	Cellular_org	Pseudomonas alcaligenes
1	Cellular_org	Pseudomonas amygdali
1	Cellular_org	Pseudomonas coronafaciens
1	Cellular_org	Pseudomonas entomophila
1	Cellular_org	Pseudomonas entomophila L48
1	Cellular_org	Pseudomonas fluorescens
1	Cellular_org	Pseudomonas fluorescens Pf-5
1	Cellular_org	Pseudomonas fluorescens Pf0-1
1	Cellular_org	Pseudomonas fluorescens SBW25
1	Cellular_org	Pseudomonas fulva
1	Cellular_org	Pseudomonas mendocina
1	Cellular_org	Pseudomonas mendocina ymp
1	Cellular_org	Pseudomonas putida

1	Cellular_org	Pseudomonas putida F1
1	Cellular_org	Pseudomonas putida GB-1
1	Cellular_org	Pseudomonas putida KT2440
1	Cellular_org	Pseudomonas putida W619
1	Cellular_org	Pseudomonas resinovorans
1	Cellular_org	Pseudomonas savastanoi
1	Cellular_org	Pseudomonas savastanoi pv. savastanoi
1	Cellular_org	Pseudomonas savastanoi pv. savastanoi NCPPB 3335
1	Cellular_org	Pseudomonas sp. ADP
1	Cellular_org	Pseudomonas sp. CA10
1	Cellular_org	Pseudomonas sp. CG21
1	Cellular_org	Pseudomonas sp. CT14
1	Cellular_org	Pseudomonas sp. ND6
1	Cellular_org	Pseudomonas sp. S-47
1	Cellular_org	Pseudomonas sp. SLT2001
1	Cellular_org	Pseudomonas sp. UK4
1	Cellular_org	Pseudomonas stutzeri
1	Cellular_org	Pseudomonas stutzeri A1501
1	Cellular_org	Pseudomonas syringae
1	Cellular_org	Pseudomonas syringae pv. aesculi
1	Cellular_org	Pseudomonas syringae pv. aesculi str. 2250
1	Cellular_org	Pseudomonas syringae pv. aesculi str. NCPPB3681
1	Cellular_org	Pseudomonas syringae pv. maculicola
1	Cellular_org	Pseudomonas syringae pv. maculicola str. M6
1	Cellular_org	Pseudomonas syringae pv. oryzae
1	Cellular_org	Pseudomonas syringae pv. oryzae str. 1_6
1	Cellular_org	Pseudomonas syringae pv. phaseolicola
1	Cellular_org	Pseudomonas syringae pv. phaseolicola 1448A
1	Cellular_org	Pseudomonas syringae pv. syringae
1	Cellular_org	Pseudomonas syringae pv. syringae 642
1	Cellular_org	Pseudomonas syringae pv. syringae B728a
1	Cellular_org	Pseudomonas syringae pv. syringae FF5
1	Cellular_org	Pseudomonas syringae pv. tabaci
1	Cellular_org	Pseudomonas syringae pv. tabaci ATCC 11528
1	Cellular_org	Pseudomonas syringae pv. tomato
1	Cellular_org	Pseudomonas syringae pv. tomato K40
1	Cellular_org	Pseudomonas syringae pv. tomato Max13
1	Cellular_org	Pseudomonas syringae pv. tomato NCPPB 1108
1	Cellular_org	Pseudomonas syringae pv. tomato T1
1	Cellular_org	Pseudomonas syringae pv. tomato str. DC3000
1	Cellular_org	Pseudonocardia autotrophica
1	Cellular_org	Pseudovibrio sp. JE062
1	Cellular_org	Psychrobacter arcticus

1	Cellular_org	Psychrobacter arcticus 273-4
1	Cellular_org	Psychrobacter cryohalolentis
1	Cellular_org	Psychrobacter cryohalolentis K5
1	Cellular_org	Psychrobacter sp. PRwf-1
1	Cellular_org	Psychroflexus torquis ATCC 700755
1	Cellular_org	Psychromonas ingrahamii 37
1	Cellular_org	Psychromonas sp. CNPT3
1	Cellular_org	Pyramidobacter piscolens W5455
1	Cellular_org	Rahnella sp. 'WMR15'
1	Cellular_org	Ralstonia eutropha H16
1	Cellular_org	Ralstonia eutropha JMP134
1	Cellular_org	Ralstonia pickettii
1	Cellular_org	Ralstonia pickettii 12D
1	Cellular_org	Ralstonia pickettii 12J
1	Cellular_org	Ralstonia solanacearum
1	Cellular_org	Ralstonia solanacearum CFBP2957
1	Cellular_org	Ralstonia solanacearum GMI1000
1	Cellular_org	Ralstonia solanacearum MolK2
1	Cellular_org	Ralstonia solanacearum PSI07
1	Cellular_org	Ralstonia solanacearum UW551
1	Cellular_org	Ralstonia sp. 5_7_47FAA
1	Cellular_org	Raphidiopsis brookii D9
1	Cellular_org	Renibacterium salmoninarum
1	Cellular_org	Renibacterium salmoninarum ATCC 33209
1	Cellular_org	Rhizobium etli
1	Cellular_org	Rhizobium etli 8C-3
1	Cellular_org	Rhizobium etli Brasil 5
1	Cellular_org	Rhizobium etli CFN 42
1	Cellular_org	Rhizobium etli CIAT 652
1	Cellular_org	Rhizobium etli CIAT 894
1	Cellular_org	Rhizobium etli GR56
1	Cellular_org	Rhizobium etli IE4771
1	Cellular_org	Rhizobium etli Kim 5
1	Cellular_org	Rhizobium leguminosarum
1	Cellular_org	Rhizobium leguminosarum bv. trifolii
1	Cellular_org	Rhizobium leguminosarum bv. trifolii WSM1325
1	Cellular_org	Rhizobium leguminosarum bv. trifolii WSM2304
1	Cellular_org	Rhizobium leguminosarum bv. viciae
1	Cellular_org	Rhizobium leguminosarum bv. viciae 3841
1	Cellular_org	Rhodobacter blasticus
1	Cellular_org	Rhodobacter capsulatus
1	Cellular_org	Rhodobacter capsulatus SB 1003
1	Cellular_org	Rhodobacter sp. SW2

1	Cellular_org	Rhodobacter sphaeroides
1	Cellular_org	Rhodobacter sphaeroides 2.4.1
1	Cellular_org	Rhodobacter sphaeroides ATCC 17025
1	Cellular_org	Rhodobacter sphaeroides ATCC 17029
1	Cellular_org	Rhodobacter sphaeroides KD131
1	Cellular_org	Rhodobacteraceae bacterium KLH11
1	Cellular_org	Rhodobacterales bacterium Y4I
1	Cellular_org	Rhodococcus aetherivorans
1	Cellular_org	Rhodococcus equi
1	Cellular_org	Rhodococcus equi ATCC 33707
1	Cellular_org	Rhodococcus erythropolis
1	Cellular_org	Rhodococcus erythropolis PR4
1	Cellular_org	Rhodococcus erythropolis SK121
1	Cellular_org	Rhodococcus jostii
1	Cellular_org	Rhodococcus jostii RHA1
1	Cellular_org	Rhodococcus opacus
1	Cellular_org	Rhodococcus opacus B4
1	Cellular_org	Rhodococcus rhodochrous
1	Cellular_org	Rhodococcus sp. B264-1
1	Cellular_org	Rhodococcus sp. NS1
1	Cellular_org	Rhodoferax ferrireducens T118
1	Cellular_org	Rhodomicrobium vannielii
1	Cellular_org	Rhodomicrobium vannielii ATCC 17100
1	Cellular_org	Rhodopirellula baltica
1	Cellular_org	Rhodopirellula baltica SH 1
1	Cellular_org	Rhodopseudomonas palustris
1	Cellular_org	Rhodopseudomonas palustris BisA53
1	Cellular_org	Rhodopseudomonas palustris BisB18
1	Cellular_org	Rhodopseudomonas palustris BisB5
1	Cellular_org	Rhodopseudomonas palustris CGA009
1	Cellular_org	Rhodopseudomonas palustris DX-1
1	Cellular_org	Rhodopseudomonas palustris HaA2
1	Cellular_org	Rhodopseudomonas palustris TIE-1
1	Cellular_org	Rhodospirillum centenum
1	Cellular_org	Rhodospirillum centenum SW
1	Cellular_org	Rhodospirillum rubrum
1	Cellular_org	Rhodospirillum rubrum ATCC 11170
1	Cellular_org	Rhodothermus marinus
1	Cellular_org	Rhodothermus marinus DSM 4252
1	Cellular_org	Rickettsia africae
1	Cellular_org	Rickettsia africae ESF-5
1	Cellular_org	Rickettsia akari str. Hartford
1	Cellular_org	Rickettsia bellii

1	Cellular_org	Rickettsia bellii OSU 85-389
1	Cellular_org	Rickettsia bellii RML369-C
1	Cellular_org	Rickettsia canadensis
1	Cellular_org	Rickettsia canadensis str. McKiel
1	Cellular_org	Rickettsia conorii
1	Cellular_org	Rickettsia conorii str. Malish 7
1	Cellular_org	Rickettsia endosymbiont of Ixodes scapularis
1	Cellular_org	Rickettsia felis
1	Cellular_org	Rickettsia felis URRWXCal2
1	Cellular_org	Rickettsia massiliae
1	Cellular_org	Rickettsia massiliae MTU5
1	Cellular_org	Rickettsia monacensis
1	Cellular_org	Rickettsia peacockii
1	Cellular_org	Rickettsia peacockii str. Rustic
1	Cellular_org	Rickettsia prowazekii
1	Cellular_org	Rickettsia prowazekii str. Madrid E
1	Cellular_org	Rickettsia rickettsii
1	Cellular_org	Rickettsia rickettsii str. 'Sheila Smith'
1	Cellular_org	Rickettsia rickettsii str. Iowa
1	Cellular_org	Rickettsia sibirica
1	Cellular_org	Rickettsia sibirica 246
1	Cellular_org	Rickettsia typhi
1	Cellular_org	Rickettsia typhi str. Wilmington
1	Cellular_org	Rickettsiella grylli
1	Cellular_org	Riemerella anatipestifer
1	Cellular_org	Robiginitalea biformata HTCC2501
1	Cellular_org	Roseburia intestinalis
1	Cellular_org	Roseburia intestinalis L1-82
1	Cellular_org	Roseburia inulinivorans
1	Cellular_org	Roseburia inulinivorans DSM 16841
1	Cellular_org	Roseibium sp. TrichSKD4
1	Cellular_org	Roseiflexus castenholzii
1	Cellular_org	Roseiflexus castenholzii DSM 13941
1	Cellular_org	Roseiflexus sp. RS-1
1	Cellular_org	Roseobacter denitrificans
1	Cellular_org	Roseobacter denitrificans OCh 114
1	Cellular_org	Roseobacter litoralis
1	Cellular_org	Roseobacter litoralis Och 149
1	Cellular_org	Roseobacter sp. AzwK-3b
1	Cellular_org	Roseobacter sp. CCS2
1	Cellular_org	Roseobacter sp. GAI101
1	Cellular_org	Roseobacter sp. MED193
1	Cellular_org	Roseobacter sp. SK209-2-6

1	Cellular_org	Roseomonas cervicalis ATCC 49957
1	Cellular_org	Roseovarius nubinhibens
1	Cellular_org	Roseovarius nubinhibens ISM
1	Cellular_org	Roseovarius sp. 217
1	Cellular_org	Roseovarius sp. TM1035
1	Cellular_org	Rothia dentocariosa
1	Cellular_org	Rothia dentocariosa ATCC 17931
1	Cellular_org	Rothia dentocariosa M567
1	Cellular_org	Rothia mucilaginosa ATCC 25296
1	Cellular_org	Rothia mucilaginosa DY-18
1	Cellular_org	Rubrobacter xylanophilus
1	Cellular_org	Rubrobacter xylanophilus DSM 9941
1	Cellular_org	Ruegeria pomeroyi
1	Cellular_org	Ruegeria pomeroyi DSS-3
1	Cellular_org	Ruegeria sp. PR1b
1	Cellular_org	Ruegeria sp. R11
1	Cellular_org	Ruegeria sp. TM1040
1	Cellular_org	Ruminococcaceae bacterium D16
1	Cellular_org	Ruminococcus albus
1	Cellular_org	Ruminococcus albus 7
1	Cellular_org	Ruminococcus albus 8
1	Cellular_org	Ruminococcus flavefaciens
1	Cellular_org	Ruminococcus flavefaciens FD-1
1	Cellular_org	Ruminococcus gnavus
1	Cellular_org	Ruminococcus gnavus ATCC 29149
1	Cellular_org	Ruminococcus lactaris ATCC 29176
1	Cellular_org	Ruminococcus obeum ATCC 29174
1	Cellular_org	Ruminococcus torques ATCC 27756
1	Cellular_org	Saccharomonospora viridis
1	Cellular_org	Saccharomonospora viridis DSM 43017
1	Cellular_org	Saccharophagus degradans 2-40
1	Cellular_org	Saccharopolyspora erythraea
1	Cellular_org	Saccharopolyspora erythraea NRRL 2338
1	Cellular_org	Sagittula stellata
1	Cellular_org	Sagittula stellata E-37
1	Cellular_org	Salinibacter ruber
1	Cellular_org	Salinibacter ruber DSM 13855
1	Cellular_org	Salinibacter ruber M8
1	Cellular_org	Salinispora arenicola
1	Cellular_org	Salinispora arenicola CNS-205
1	Cellular_org	Salinispora tropica
1	Cellular_org	Salinispora tropica CNB-440
1	Cellular_org	Salmonella enterica

1	Cellular_org	Salmonella enterica subsp. arizonae
1	Cellular_org	Salmonella enterica subsp. arizonae serovar 62:z4,z23:--
1	Cellular_org	Salmonella enterica subsp. arizonae serovar 62:z4,z23:-- str. RSK2980
1	Cellular_org	Salmonella enterica subsp. enterica
1	Cellular_org	Salmonella enterica subsp. enterica serovar Agona
1	Cellular_org	Salmonella enterica subsp. enterica serovar Agona str. SL483
1	Cellular_org	Salmonella enterica subsp. enterica serovar Berta
1	Cellular_org	Salmonella enterica subsp. enterica serovar Bovismorbificans
1	Cellular_org	Salmonella enterica subsp. enterica serovar Brandenburg
1	Cellular_org	Salmonella enterica subsp. enterica serovar Choleraesuis
1	Cellular_org	Salmonella enterica subsp. enterica serovar Choleraesuis str. SC-B67
1	Cellular_org	Salmonella enterica subsp. enterica serovar Dublin
1	Cellular_org	Salmonella enterica subsp. enterica serovar Dublin str. CT_02021853
1	Cellular_org	Salmonella enterica subsp. enterica serovar Enteritidis
1	Cellular_org	Salmonella enterica subsp. enterica serovar Enteritidis str. P125109
1	Cellular_org	Salmonella enterica subsp. enterica serovar Gallinarum
1	Cellular_org	Salmonella enterica subsp. enterica serovar Gallinarum str. 287/91
1	Cellular_org	Salmonella enterica subsp. enterica serovar Hadar
1	Cellular_org	Salmonella enterica subsp. enterica serovar Hadar str. RI_05P066
1	Cellular_org	Salmonella enterica subsp. enterica serovar Heidelberg
1	Cellular_org	Salmonella enterica subsp. enterica serovar Heidelberg str. SL476
1	Cellular_org	Salmonella enterica subsp. enterica serovar Heidelberg str. SL486
1	Cellular_org	Salmonella enterica subsp. enterica serovar Javiana
1	Cellular_org	Salmonella enterica subsp. enterica serovar Javiana str. GA_MM04042433
1	Cellular_org	Salmonella enterica subsp. enterica serovar Kentucky
1	Cellular_org	Salmonella enterica subsp. enterica serovar Kentucky str. CDC 191
1	Cellular_org	Salmonella enterica subsp. enterica serovar Kentucky str. CVM29188
1	Cellular_org	Salmonella enterica subsp. enterica serovar Newport
1	Cellular_org	Salmonella enterica subsp. enterica serovar Newport str. SL254

1	Cellular_org	Salmonella enterica subsp. enterica serovar Newport str. SL317
1	Cellular_org	Salmonella enterica subsp. enterica serovar Paratyphi A
1	Cellular_org	Salmonella enterica subsp. enterica serovar Paratyphi A str. AKU_12601
1	Cellular_org	Salmonella enterica subsp. enterica serovar Paratyphi A str. ATCC 9150
1	Cellular_org	Salmonella enterica subsp. enterica serovar Paratyphi B
1	Cellular_org	Salmonella enterica subsp. enterica serovar Paratyphi B str. SPB7
1	Cellular_org	Salmonella enterica subsp. enterica serovar Paratyphi C
1	Cellular_org	Salmonella enterica subsp. enterica serovar Paratyphi C strain RKS4594
1	Cellular_org	Salmonella enterica subsp. enterica serovar Saintpaul
1	Cellular_org	Salmonella enterica subsp. enterica serovar Saintpaul str. SARA23
1	Cellular_org	Salmonella enterica subsp. enterica serovar Saintpaul str. SARA29
1	Cellular_org	Salmonella enterica subsp. enterica serovar Schwarzengrund
1	Cellular_org	Salmonella enterica subsp. enterica serovar Schwarzengrund str. CVM19633
1	Cellular_org	Salmonella enterica subsp. enterica serovar Schwarzengrund str. SL480
1	Cellular_org	Salmonella enterica subsp. enterica serovar Tennessee
1	Cellular_org	Salmonella enterica subsp. enterica serovar Tennessee str. CDC07-0191
1	Cellular_org	Salmonella enterica subsp. enterica serovar Typhi
1	Cellular_org	Salmonella enterica subsp. enterica serovar Typhi str. 404ty
1	Cellular_org	Salmonella enterica subsp. enterica serovar Typhi str. AG3
1	Cellular_org	Salmonella enterica subsp. enterica serovar Typhi str. CT18
1	Cellular_org	Salmonella enterica subsp. enterica serovar Typhi str. E00-7866
1	Cellular_org	Salmonella enterica subsp. enterica serovar Typhi str. E01-6750
1	Cellular_org	Salmonella enterica subsp. enterica serovar Typhi str. E02-1180
1	Cellular_org	Salmonella enterica subsp. enterica serovar Typhi str. E98-0664
1	Cellular_org	Salmonella enterica subsp. enterica serovar Typhi str. E98-2068

1	Cellular_org	Salmonella enterica subsp. enterica serovar Typhi str. E98-3139
1	Cellular_org	Salmonella enterica subsp. enterica serovar Typhi str. J185
1	Cellular_org	Salmonella enterica subsp. enterica serovar Typhi str. M223
1	Cellular_org	Salmonella enterica subsp. enterica serovar Typhi str. Ty2
1	Cellular_org	Salmonella enterica subsp. enterica serovar Typhimurium
1	Cellular_org	Salmonella enterica subsp. enterica serovar Typhimurium str. LT2
1	Cellular_org	Salmonella enterica subsp. enterica serovar Virchow
1	Cellular_org	Salmonella enterica subsp. enterica serovar Virchow str. SL491
1	Cellular_org	Salmonella enterica subsp. enterica serovar Weltevreden
1	Cellular_org	Salmonella enterica subsp. enterica serovar Weltevreden str. HI_N05-537
1	Cellular_org	Salmonella enterica subsp. enterica serovar Westhampton
1	Cellular_org	Sanguibacter keddieii DSM 10542
1	Cellular_org	Scardovia inopinata
1	Cellular_org	Scardovia inopinata F0304
1	Cellular_org	Sebaldella termitidis ATCC 33386
1	Cellular_org	Segniliparus rotundus DSM 44985
1	Cellular_org	Segniliparus rugosus
1	Cellular_org	Selenomonas flueggei ATCC 43531
1	Cellular_org	Selenomonas noxia ATCC 43541
1	Cellular_org	Selenomonas ruminantium
1	Cellular_org	Selenomonas sp. oral taxon 149 str. 67H29BP
1	Cellular_org	Selenomonas sputigena ATCC 35185
1	Cellular_org	Serratia entomophila
1	Cellular_org	Serratia marcescens
1	Cellular_org	Serratia odorifera
1	Cellular_org	Serratia odorifera 4Rx13
1	Cellular_org	Serratia odorifera DSM 4582
1	Cellular_org	Serratia proteamaculans
1	Cellular_org	Serratia proteamaculans 568
1	Cellular_org	Shewanella amazonensis SB2B
1	Cellular_org	Shewanella baltica
1	Cellular_org	Shewanella baltica BA175
1	Cellular_org	Shewanella baltica OS155
1	Cellular_org	Shewanella baltica OS183
1	Cellular_org	Shewanella baltica OS185
1	Cellular_org	Shewanella baltica OS195
1	Cellular_org	Shewanella baltica OS223

1	Cellular_org	Shewanella benthica
1	Cellular_org	Shewanella benthica KT99
1	Cellular_org	Shewanella denitrificans OS217
1	Cellular_org	Shewanella frigidimarina
1	Cellular_org	Shewanella frigidimarina NCIMB 400
1	Cellular_org	Shewanella halifaxensis HAW-EB4
1	Cellular_org	Shewanella loihica PV-4
1	Cellular_org	Shewanella oneidensis
1	Cellular_org	Shewanella oneidensis MR-1
1	Cellular_org	Shewanella pealeana
1	Cellular_org	Shewanella pealeana ATCC 700345
1	Cellular_org	Shewanella piezotolerans WP3
1	Cellular_org	Shewanella putrefaciens
1	Cellular_org	Shewanella putrefaciens CN-32
1	Cellular_org	Shewanella sediminis
1	Cellular_org	Shewanella sediminis HAW-EB3
1	Cellular_org	Shewanella sp. 33B
1	Cellular_org	Shewanella sp. ANA-3
1	Cellular_org	Shewanella sp. MR-4
1	Cellular_org	Shewanella sp. MR-7
1	Cellular_org	Shewanella sp. W3-18-1
1	Cellular_org	Shewanella violacea
1	Cellular_org	Shewanella violacea DSS12
1	Cellular_org	Shewanella woodyi
1	Cellular_org	Shewanella woodyi ATCC 51908
1	Cellular_org	Shigella boydii
1	Cellular_org	Shigella boydii CDC 3083-94
1	Cellular_org	Shigella boydii Sb227
1	Cellular_org	Shigella dysenteriae
1	Cellular_org	Shigella dysenteriae 1012
1	Cellular_org	Shigella dysenteriae 1617
1	Cellular_org	Shigella dysenteriae Sd197
1	Cellular_org	Shigella flexneri
1	Cellular_org	Shigella flexneri 2a
1	Cellular_org	Shigella flexneri 2a str. 2457T
1	Cellular_org	Shigella flexneri 2a str. 301
1	Cellular_org	Shigella flexneri 5
1	Cellular_org	Shigella flexneri 5 str. 8401
1	Cellular_org	Shigella flexneri 5a
1	Cellular_org	Shigella sonnei
1	Cellular_org	Shigella sonnei Ss046
1	Cellular_org	Shigella sp. D9
1	Cellular_org	Shuttleworthia satelles DSM 14600

1	Cellular_org	Sideroxydans lithotrophicus ES-1
1	Cellular_org	Silicibacter lacuscaerulensis ITI-1157
1	Cellular_org	Silicibacter sp. TrichCH4B
1	Cellular_org	Simonsiella muelleri ATCC 29453
1	Cellular_org	Sinorhizobium fredii
1	Cellular_org	Sinorhizobium medicae
1	Cellular_org	Sinorhizobium medicae WSM419
1	Cellular_org	Sinorhizobium meliloti
1	Cellular_org	Sinorhizobium meliloti 1021
1	Cellular_org	Sinorhizobium meliloti AK83
1	Cellular_org	Sinorhizobium meliloti BL225C
1	Cellular_org	Sinorhizobium meliloti SM11
1	Cellular_org	Slackia exigua ATCC 700122
1	Cellular_org	Slackia heliotrinireducens DSM 20476
1	Cellular_org	Sodalis glossinidius
1	Cellular_org	Sodalis glossinidius str. 'morsitans'
1	Cellular_org	Sorangium cellulosum
1	Cellular_org	Sorangium cellulosum 'So ce 56'
1	Cellular_org	Sphaerobacter thermophilus DSM 20745
1	Cellular_org	Sphingobacterium spiritivorum
1	Cellular_org	Sphingobacterium spiritivorum ATCC 33300
1	Cellular_org	Sphingobacterium spiritivorum ATCC 33861
1	Cellular_org	Sphingobium chlorophenolicum
1	Cellular_org	Sphingobium chlorophenolicum L-1
1	Cellular_org	Sphingobium japonicum
1	Cellular_org	Sphingobium japonicum UT26S
1	Cellular_org	Sphingobium xenophagum
1	Cellular_org	Sphingobium yanoikuyae
1	Cellular_org	Sphingomonas sp. A1
1	Cellular_org	Sphingomonas sp. KA1
1	Cellular_org	Sphingomonas sp. MM-1
1	Cellular_org	Sphingomonas sp. SKA58
1	Cellular_org	Sphingomonas wittichii RW1
1	Cellular_org	Sphingopyxis alaskensis RB2256
1	Cellular_org	Sphingopyxis macrogoltabida
1	Cellular_org	Spirochaeta smaragdinae DSM 11293
1	Cellular_org	Spirochaeta thermophila
1	Cellular_org	Spirochaeta thermophila DSM 6192
1	Cellular_org	Spiroplasma citri
1	Cellular_org	Spiroplasma kunkelii
1	Cellular_org	Spiroplasma kunkelii CR2-3x
1	Cellular_org	Spirosoma linguale DSM 74
1	Cellular_org	Sporosarcina ureae

1	Cellular_org	Stackebrandtia nassauensis DSM 44728
1	Cellular_org	Staphylococcus aureus
1	Cellular_org	Staphylococcus aureus 930918-3
1	Cellular_org	Staphylococcus aureus A10102
1	Cellular_org	Staphylococcus aureus A5937
1	Cellular_org	Staphylococcus aureus A5948
1	Cellular_org	Staphylococcus aureus A6224
1	Cellular_org	Staphylococcus aureus A6300
1	Cellular_org	Staphylococcus aureus A8115
1	Cellular_org	Staphylococcus aureus A8117
1	Cellular_org	Staphylococcus aureus A8796
1	Cellular_org	Staphylococcus aureus A8819
1	Cellular_org	Staphylococcus aureus A9299
1	Cellular_org	Staphylococcus aureus A9635
1	Cellular_org	Staphylococcus aureus A9719
1	Cellular_org	Staphylococcus aureus A9754
1	Cellular_org	Staphylococcus aureus A9763
1	Cellular_org	Staphylococcus aureus A9765
1	Cellular_org	Staphylococcus aureus A9781
1	Cellular_org	Staphylococcus aureus D30
1	Cellular_org	Staphylococcus aureus RF122
1	Cellular_org	Staphylococcus aureus subsp. aureus
1	Cellular_org	Staphylococcus aureus subsp. aureus 132
1	Cellular_org	Staphylococcus aureus subsp. aureus 55/2053
1	Cellular_org	Staphylococcus aureus subsp. aureus 58-424
1	Cellular_org	Staphylococcus aureus subsp. aureus 65-1322
1	Cellular_org	Staphylococcus aureus subsp. aureus 68-397
1	Cellular_org	Staphylococcus aureus subsp. aureus A017934/97
1	Cellular_org	Staphylococcus aureus subsp. aureus ATCC 51811
1	Cellular_org	Staphylococcus aureus subsp. aureus ATCC BAA-39
1	Cellular_org	Staphylococcus aureus subsp. aureus Btn1260
1	Cellular_org	Staphylococcus aureus subsp. aureus C101
1	Cellular_org	Staphylococcus aureus subsp. aureus C160
1	Cellular_org	Staphylococcus aureus subsp. aureus C427
1	Cellular_org	Staphylococcus aureus subsp. aureus COL
1	Cellular_org	Staphylococcus aureus subsp. aureus D139
1	Cellular_org	Staphylococcus aureus subsp. aureus E1410
1	Cellular_org	Staphylococcus aureus subsp. aureus ED98
1	Cellular_org	Staphylococcus aureus subsp. aureus EMRSA16
1	Cellular_org	Staphylococcus aureus subsp. aureus H19
1	Cellular_org	Staphylococcus aureus subsp. aureus JH1
1	Cellular_org	Staphylococcus aureus subsp. aureus JH9
1	Cellular_org	Staphylococcus aureus subsp. aureus M1015

1	Cellular_org	Staphylococcus aureus subsp. aureus M809
1	Cellular_org	Staphylococcus aureus subsp. aureus M876
1	Cellular_org	Staphylococcus aureus subsp. aureus M899
1	Cellular_org	Staphylococcus aureus subsp. aureus MN8
1	Cellular_org	Staphylococcus aureus subsp. aureus MR1
1	Cellular_org	Staphylococcus aureus subsp. aureus MRSA252
1	Cellular_org	Staphylococcus aureus subsp. aureus MSSA476
1	Cellular_org	Staphylococcus aureus subsp. aureus MW2
1	Cellular_org	Staphylococcus aureus subsp. aureus Mu3
1	Cellular_org	Staphylococcus aureus subsp. aureus Mu50
1	Cellular_org	Staphylococcus aureus subsp. aureus Mu50-omega
1	Cellular_org	Staphylococcus aureus subsp. aureus N315
1	Cellular_org	Staphylococcus aureus subsp. aureus NCTC 8325
1	Cellular_org	Staphylococcus aureus subsp. aureus ST398
1	Cellular_org	Staphylococcus aureus subsp. aureus TCH130
1	Cellular_org	Staphylococcus aureus subsp. aureus TCH70
1	Cellular_org	Staphylococcus aureus subsp. aureus USA300_FPR3757
1	Cellular_org	Staphylococcus aureus subsp. aureus USA300_TCH1516
1	Cellular_org	Staphylococcus aureus subsp. aureus USA300_TCH959
1	Cellular_org	Staphylococcus aureus subsp. aureus WBG10049
1	Cellular_org	Staphylococcus aureus subsp. aureus WW2703/97
1	Cellular_org	Staphylococcus aureus subsp. aureus str. CF-Marseille
1	Cellular_org	Staphylococcus aureus subsp. aureus str. JKD6009
1	Cellular_org	Staphylococcus aureus subsp. aureus str. Newman
1	Cellular_org	Staphylococcus capitis
1	Cellular_org	Staphylococcus capitis SK14
1	Cellular_org	Staphylococcus caprae
1	Cellular_org	Staphylococcus carnosus
1	Cellular_org	Staphylococcus carnosus subsp. carnosus
1	Cellular_org	Staphylococcus carnosus subsp. carnosus TM300
1	Cellular_org	Staphylococcus chromogenes
1	Cellular_org	Staphylococcus epidermidis
1	Cellular_org	Staphylococcus epidermidis ATCC 12228
1	Cellular_org	Staphylococcus epidermidis BCM-HMP0060
1	Cellular_org	Staphylococcus epidermidis M23864:W1
1	Cellular_org	Staphylococcus epidermidis M23864:W2(grey)
1	Cellular_org	Staphylococcus epidermidis RP62A
1	Cellular_org	Staphylococcus epidermidis SK135
1	Cellular_org	Staphylococcus epidermidis W23144
1	Cellular_org	Staphylococcus haemolyticus
1	Cellular_org	Staphylococcus haemolyticus JCSC1435
1	Cellular_org	Staphylococcus hominis
1	Cellular_org	Staphylococcus hominis SK119

1	Cellular_org	Staphylococcus hominis subsp. hominis
1	Cellular_org	Staphylococcus lentus
1	Cellular_org	Staphylococcus lugdunensis
1	Cellular_org	Staphylococcus lugdunensis HKU09-01
1	Cellular_org	Staphylococcus pasteuri
1	Cellular_org	Staphylococcus pseudintermedius
1	Cellular_org	Staphylococcus saprophyticus
1	Cellular_org	Staphylococcus saprophyticus subsp. saprophyticus
1	Cellular_org	Staphylococcus saprophyticus subsp. saprophyticus ATCC 15305
1	Cellular_org	Staphylococcus sciuri
1	Cellular_org	Staphylococcus sciuri subsp. sciuri
1	Cellular_org	Staphylococcus simulans
1	Cellular_org	Staphylococcus simulans bv. staphylolyticus
1	Cellular_org	Staphylococcus sp. 693-2
1	Cellular_org	Staphylococcus warneri
1	Cellular_org	Staphylococcus warneri L37603
1	Cellular_org	Starkeya novella DSM 506
1	Cellular_org	Stenotrophomonas maltophilia
1	Cellular_org	Stenotrophomonas maltophilia K279a
1	Cellular_org	Stenotrophomonas maltophilia R551-3
1	Cellular_org	Stenotrophomonas sp. SKA14
1	Cellular_org	Stigmatella aurantiaca
1	Cellular_org	Stigmatella aurantiaca DW4/3-1
1	Cellular_org	Streptobacillus moniliformis
1	Cellular_org	Streptobacillus moniliformis DSM 12112
1	Cellular_org	Streptococcus
1	Cellular_org	Streptococcus agalactiae
1	Cellular_org	Streptococcus agalactiae 18RS21
1	Cellular_org	Streptococcus agalactiae 2603V/R
1	Cellular_org	Streptococcus agalactiae 515
1	Cellular_org	Streptococcus agalactiae A909
1	Cellular_org	Streptococcus agalactiae CJB111
1	Cellular_org	Streptococcus agalactiae COH1
1	Cellular_org	Streptococcus agalactiae H36B
1	Cellular_org	Streptococcus agalactiae NEM316
1	Cellular_org	Streptococcus agalactiae serogroup III
1	Cellular_org	Streptococcus anginosus
1	Cellular_org	Streptococcus australis
1	Cellular_org	Streptococcus bovis ATCC 700338
1	Cellular_org	Streptococcus cristatus
1	Cellular_org	Streptococcus cristatus ATCC 51100
1	Cellular_org	Streptococcus downei

1	Cellular_org	Streptococcus dysgalactiae
1	Cellular_org	Streptococcus dysgalactiae subsp. equisimilis
1	Cellular_org	Streptococcus dysgalactiae subsp. equisimilis GGS_124
1	Cellular_org	Streptococcus equi
1	Cellular_org	Streptococcus equi subsp. equi
1	Cellular_org	Streptococcus equi subsp. equi 4047
1	Cellular_org	Streptococcus equi subsp. zooepidemicus
1	Cellular_org	Streptococcus equi subsp. zooepidemicus MGCS10565
1	Cellular_org	Streptococcus equinus
1	Cellular_org	Streptococcus gallolyticus
1	Cellular_org	Streptococcus gallolyticus UCN34
1	Cellular_org	Streptococcus gallolyticus subsp. gallolyticus
1	Cellular_org	Streptococcus gallolyticus subsp. gallolyticus TX20005
1	Cellular_org	Streptococcus gordonii
1	Cellular_org	Streptococcus gordonii str. Challis
1	Cellular_org	Streptococcus gordonii str. Challis substr. CH1
1	Cellular_org	Streptococcus infantarius
1	Cellular_org	Streptococcus infantarius subsp. infantarius
1	Cellular_org	Streptococcus infantarius subsp. infantarius ATCC BAA-102
1	Cellular_org	Streptococcus infantis
1	Cellular_org	Streptococcus infantis ATCC 700779
1	Cellular_org	Streptococcus infantis SK1302
1	Cellular_org	Streptococcus mitis
1	Cellular_org	Streptococcus mitis ATCC 6249
1	Cellular_org	Streptococcus mitis B6
1	Cellular_org	Streptococcus mitis NCTC 12261
1	Cellular_org	Streptococcus mitis SK321
1	Cellular_org	Streptococcus mitis SK564
1	Cellular_org	Streptococcus mitis SK597
1	Cellular_org	Streptococcus mutans
1	Cellular_org	Streptococcus mutans NN2025
1	Cellular_org	Streptococcus mutans UA159
1	Cellular_org	Streptococcus oralis
1	Cellular_org	Streptococcus oralis ATCC 35037
1	Cellular_org	Streptococcus parasanguinis
1	Cellular_org	Streptococcus parasanguinis ATCC 15912
1	Cellular_org	Streptococcus peroris
1	Cellular_org	Streptococcus peroris ATCC 700780
1	Cellular_org	Streptococcus pneumoniae
1	Cellular_org	Streptococcus pneumoniae 670-6B
1	Cellular_org	Streptococcus pneumoniae 70585
1	Cellular_org	Streptococcus pneumoniae AP200

1	Cellular_org	Streptococcus pneumoniae ATCC 700669
1	Cellular_org	Streptococcus pneumoniae BS397
1	Cellular_org	Streptococcus pneumoniae BS455
1	Cellular_org	Streptococcus pneumoniae BS457
1	Cellular_org	Streptococcus pneumoniae BS458
1	Cellular_org	Streptococcus pneumoniae CCRI 1974
1	Cellular_org	Streptococcus pneumoniae CCRI 1974M2
1	Cellular_org	Streptococcus pneumoniae CDC0288-04
1	Cellular_org	Streptococcus pneumoniae CDC1087-00
1	Cellular_org	Streptococcus pneumoniae CDC1873-00
1	Cellular_org	Streptococcus pneumoniae CDC3059-06
1	Cellular_org	Streptococcus pneumoniae CGSP14
1	Cellular_org	Streptococcus pneumoniae D39
1	Cellular_org	Streptococcus pneumoniae G54
1	Cellular_org	Streptococcus pneumoniae Hungary19A-6
1	Cellular_org	Streptococcus pneumoniae JJA
1	Cellular_org	Streptococcus pneumoniae MLV-016
1	Cellular_org	Streptococcus pneumoniae P1031
1	Cellular_org	Streptococcus pneumoniae R6
1	Cellular_org	Streptococcus pneumoniae SP-BS293
1	Cellular_org	Streptococcus pneumoniae SP11-BS70
1	Cellular_org	Streptococcus pneumoniae SP14-BS292
1	Cellular_org	Streptococcus pneumoniae SP14-BS69
1	Cellular_org	Streptococcus pneumoniae SP18-BS74
1	Cellular_org	Streptococcus pneumoniae SP19-BS75
1	Cellular_org	Streptococcus pneumoniae SP195
1	Cellular_org	Streptococcus pneumoniae SP23-BS72
1	Cellular_org	Streptococcus pneumoniae SP3-BS71
1	Cellular_org	Streptococcus pneumoniae SP6-BS73
1	Cellular_org	Streptococcus pneumoniae SP9-BS68
1	Cellular_org	Streptococcus pneumoniae TCH8431/19A
1	Cellular_org	Streptococcus pneumoniae TIGR4
1	Cellular_org	Streptococcus pneumoniae Taiwan19F-14
1	Cellular_org	Streptococcus pneumoniae str. Canada MDR_19A
1	Cellular_org	Streptococcus pneumoniae str. Canada MDR_19F
1	Cellular_org	Streptococcus pyogenes
1	Cellular_org	Streptococcus pyogenes ATCC 10782
1	Cellular_org	Streptococcus pyogenes M1 GAS
1	Cellular_org	Streptococcus pyogenes M49 591
1	Cellular_org	Streptococcus pyogenes MGAS10270
1	Cellular_org	Streptococcus pyogenes MGAS10394
1	Cellular_org	Streptococcus pyogenes MGAS10750
1	Cellular_org	Streptococcus pyogenes MGAS2096

1	Cellular_org	Streptococcus pyogenes MGAS315
1	Cellular_org	Streptococcus pyogenes MGAS5005
1	Cellular_org	Streptococcus pyogenes MGAS6180
1	Cellular_org	Streptococcus pyogenes MGAS8232
1	Cellular_org	Streptococcus pyogenes MGAS9429
1	Cellular_org	Streptococcus pyogenes NZ131
1	Cellular_org	Streptococcus pyogenes SSI-1
1	Cellular_org	Streptococcus pyogenes serotype M12
1	Cellular_org	Streptococcus pyogenes serotype M3
1	Cellular_org	Streptococcus pyogenes serotype M5
1	Cellular_org	Streptococcus pyogenes serotype M6
1	Cellular_org	Streptococcus pyogenes str. Manfredo
1	Cellular_org	Streptococcus salivarius
1	Cellular_org	Streptococcus salivarius SK126
1	Cellular_org	Streptococcus sanguinis
1	Cellular_org	Streptococcus sanguinis SK36
1	Cellular_org	Streptococcus sp. 2_1_36FAA
1	Cellular_org	Streptococcus sp. C150
1	Cellular_org	Streptococcus sp. C300
1	Cellular_org	Streptococcus sp. M143
1	Cellular_org	Streptococcus sp. oral taxon 071 str. 73H25AP
1	Cellular_org	Streptococcus suis
1	Cellular_org	Streptococcus suis 05HAS68
1	Cellular_org	Streptococcus suis 05ZYH33
1	Cellular_org	Streptococcus suis 89/1591
1	Cellular_org	Streptococcus suis 98HAH33
1	Cellular_org	Streptococcus suis BM407
1	Cellular_org	Streptococcus suis P1/7
1	Cellular_org	Streptococcus suis SC84
1	Cellular_org	Streptococcus thermophilus
1	Cellular_org	Streptococcus thermophilus CNRZ1066
1	Cellular_org	Streptococcus thermophilus LMD-9
1	Cellular_org	Streptococcus thermophilus LMG 18311
1	Cellular_org	Streptococcus uberis
1	Cellular_org	Streptococcus uberis 0140J
1	Cellular_org	Streptococcus vestibularis
1	Cellular_org	Streptococcus vestibularis ATCC 49124
1	Cellular_org	Streptomyces albulus
1	Cellular_org	Streptomyces albus
1	Cellular_org	Streptomyces albus J1074
1	Cellular_org	Streptomyces avermitilis
1	Cellular_org	Streptomyces avermitilis MA-4680
1	Cellular_org	Streptomyces clavuligerus

1	Cellular_org	Streptomyces clavuligerus ATCC 27064
1	Cellular_org	Streptomyces coelicolor
1	Cellular_org	Streptomyces coelicolor A3(2)
1	Cellular_org	Streptomyces cyaneus
1	Cellular_org	Streptomyces filamentosus
1	Cellular_org	Streptomyces flavovirens
1	Cellular_org	Streptomyces ghanaensis
1	Cellular_org	Streptomyces ghanaensis ATCC 14672
1	Cellular_org	Streptomyces griseoflavus
1	Cellular_org	Streptomyces griseoflavus Tu4000
1	Cellular_org	Streptomyces griseus
1	Cellular_org	Streptomyces griseus subsp. griseus
1	Cellular_org	Streptomyces griseus subsp. griseus NBRC 13350
1	Cellular_org	Streptomyces hygroscopicus
1	Cellular_org	Streptomyces hygroscopicus ATCC 53653
1	Cellular_org	Streptomyces laurentii
1	Cellular_org	Streptomyces lavendulae
1	Cellular_org	Streptomyces lividans
1	Cellular_org	Streptomyces lividans TK24
1	Cellular_org	Streptomyces natalensis
1	Cellular_org	Streptomyces phaeochromogenes
1	Cellular_org	Streptomyces pristinaespiralis
1	Cellular_org	Streptomyces pristinaespiralis ATCC 25486
1	Cellular_org	Streptomyces rochei
1	Cellular_org	Streptomyces roseosporus NRRL 11379
1	Cellular_org	Streptomyces roseosporus NRRL 15998
1	Cellular_org	Streptomyces scabiei
1	Cellular_org	Streptomyces scabiei 87.22
1	Cellular_org	Streptomyces sp. 44030
1	Cellular_org	Streptomyces sp. 44414
1	Cellular_org	Streptomyces sp. AA4
1	Cellular_org	Streptomyces sp. ACT-1
1	Cellular_org	Streptomyces sp. ACTE
1	Cellular_org	Streptomyces sp. C
1	Cellular_org	Streptomyces sp. EN27
1	Cellular_org	Streptomyces sp. F11
1	Cellular_org	Streptomyces sp. FQ1
1	Cellular_org	Streptomyces sp. FR1
1	Cellular_org	Streptomyces sp. HK1
1	Cellular_org	Streptomyces sp. Mg1
1	Cellular_org	Streptomyces sp. SPB74
1	Cellular_org	Streptomyces sp. SPB78
1	Cellular_org	Streptomyces sp. W9

1	Cellular_org	Streptomyces sp. Y27
1	Cellular_org	Streptomyces sp. ZL12
1	Cellular_org	Streptomyces sp. e14
1	Cellular_org	Streptomyces sp. x3
1	Cellular_org	Streptomyces sviceus ATCC 29083
1	Cellular_org	Streptomyces venezuelae
1	Cellular_org	Streptomyces violaceoruber
1	Cellular_org	Streptomyces violaceusniger
1	Cellular_org	Streptomyces violaceusniger Tu 4113
1	Cellular_org	Streptomyces viridochromogenes
1	Cellular_org	Streptomyces viridochromogenes DSM 40736
1	Cellular_org	Streptosporangium roseum
1	Cellular_org	Streptosporangium roseum DSM 43021
1	Cellular_org	Subdoligranulum variabile DSM 15176
1	Cellular_org	Sulfitobacter sp. EE-36
1	Cellular_org	Sulfitobacter sp. NAS-14.1
1	Cellular_org	Sulfurihydrogenibium azorense
1	Cellular_org	Sulfurihydrogenibium azorense Az-Fu1
1	Cellular_org	Sulfurihydrogenibium sp. YO3AOP1
1	Cellular_org	Sulfurihydrogenibium yellowstonense
1	Cellular_org	Sulfurihydrogenibium yellowstonense SS-5
1	Cellular_org	Sulfurimonas autotrophica
1	Cellular_org	Sulfurimonas autotrophica DSM 16294
1	Cellular_org	Sulfurimonas denitrificans
1	Cellular_org	Sulfurimonas denitrificans DSM 1251
1	Cellular_org	Sulfurospirillum deleyianum
1	Cellular_org	Sulfurospirillum deleyianum DSM 6946
1	Cellular_org	Sulfurovum sp. NBC37-1
1	Cellular_org	Symbiobacterium thermophilum
1	Cellular_org	Symbiobacterium thermophilum IAM 14863
1	Cellular_org	Synechococcus
1	Cellular_org	Synechococcus elongatus
1	Cellular_org	Synechococcus elongatus PCC 6301
1	Cellular_org	Synechococcus elongatus PCC 7942
1	Cellular_org	Synechococcus sp.
1	Cellular_org	Synechococcus sp. BL107
1	Cellular_org	Synechococcus sp. CB0101
1	Cellular_org	Synechococcus sp. CB0205
1	Cellular_org	Synechococcus sp. CC9311
1	Cellular_org	Synechococcus sp. CC9605
1	Cellular_org	Synechococcus sp. CC9902
1	Cellular_org	Synechococcus sp. JA-2-3B'a(2-13)
1	Cellular_org	Synechococcus sp. JA-3-3Ab

1	Cellular_org	Synechococcus sp. PCC 7002
1	Cellular_org	Synechococcus sp. PCC 7335
1	Cellular_org	Synechococcus sp. RCC307
1	Cellular_org	Synechococcus sp. RS9916
1	Cellular_org	Synechococcus sp. RS9917
1	Cellular_org	Synechococcus sp. WH 5701
1	Cellular_org	Synechococcus sp. WH 7803
1	Cellular_org	Synechococcus sp. WH 7805
1	Cellular_org	Synechococcus sp. WH 8102
1	Cellular_org	Synechococcus sp. WH 8109
1	Cellular_org	Synechocystis
1	Cellular_org	Synechocystis sp. PCC 6803
1	Cellular_org	Syntrophobacter fumaroxidans MPOB
1	Cellular_org	Syntrophomonas wolfei subsp. wolfei
1	Cellular_org	Syntrophomonas wolfei subsp. wolfei str. Goettingen
1	Cellular_org	Syntrophothermus lipocalidus DSM 12680
1	Cellular_org	Syntrophus aciditrophicus SB
1	Cellular_org	Teredinibacter turnerae
1	Cellular_org	Teredinibacter turnerae T7901
1	Cellular_org	Tetragenococcus halophilus
1	Cellular_org	Thalassiobium sp. R2A62
1	Cellular_org	Thauera sp. MZ1T
1	Cellular_org	Thermaerobacter marianensis DSM 12885
1	Cellular_org	Thermanaerovibrio acidaminovorans DSM 6589
1	Cellular_org	Thermincola potens JR
1	Cellular_org	Thermoanaerobacter brockii
1	Cellular_org	Thermoanaerobacter brockii subsp. finnii Ako-1
1	Cellular_org	Thermoanaerobacter ethanolicus
1	Cellular_org	Thermoanaerobacter ethanolicus CCSD1
1	Cellular_org	Thermoanaerobacter italicus Ab9
1	Cellular_org	Thermoanaerobacter mathranii
1	Cellular_org	Thermoanaerobacter mathranii subsp. mathranii str. A3
1	Cellular_org	Thermoanaerobacter pseudethanolicus ATCC 33223
1	Cellular_org	Thermoanaerobacter sp. X513
1	Cellular_org	Thermoanaerobacter sp. X514
1	Cellular_org	Thermoanaerobacter sp. X561
1	Cellular_org	Thermoanaerobacter tengcongensis MB4
1	Cellular_org	Thermoanaerobacter wiegelii Rt8.B1
1	Cellular_org	Thermoanaerobacterium thermosaccharolyticum
1	Cellular_org	Thermoanaerobacterium thermosaccharolyticum DSM 571
1	Cellular_org	Thermoanaerobacterium xylanolyticum LX-11
1	Cellular_org	Thermobaculum terrenum ATCC BAA-798

1	Cellular_org	Thermobifida fusca
1	Cellular_org	Thermobifida fusca YX
1	Cellular_org	Thermobispora bispora
1	Cellular_org	Thermobispora bispora DSM 43833
1	Cellular_org	Thermocrinis albus DSM 14484
1	Cellular_org	Thermodesulfovibrio yellowstonii
1	Cellular_org	Thermodesulfovibrio yellowstonii DSM 11347
1	Cellular_org	Thermomicrobium roseum
1	Cellular_org	Thermomicrobium roseum DSM 5159
1	Cellular_org	Thermomonospora curvata
1	Cellular_org	Thermomonospora curvata DSM 43183
1	Cellular_org	Thermosediminibacter oceani DSM 16646
1	Cellular_org	Thermosinus carboxydivorans Nor1
1	Cellular_org	Thermosipho africanus TCF52B
1	Cellular_org	Thermosipho melanesiensis BI429
1	Cellular_org	Thermosynechococcus elongatus
1	Cellular_org	Thermosynechococcus elongatus BP-1
1	Cellular_org	Thermotoga lettingae TMO
1	Cellular_org	Thermotoga maritima
1	Cellular_org	Thermotoga maritima MSB8
1	Cellular_org	Thermotoga naphthophila
1	Cellular_org	Thermotoga naphthophila RKU-10
1	Cellular_org	Thermotoga neapolitana
1	Cellular_org	Thermotoga neapolitana DSM 4359
1	Cellular_org	Thermotoga petrophila
1	Cellular_org	Thermotoga petrophila RKU-1
1	Cellular_org	Thermotoga sp. RQ2
1	Cellular_org	Thermotogales bacterium mesG1.Ag.4.2
1	Cellular_org	Thermovibrio ammonificans
1	Cellular_org	Thermus aquaticus
1	Cellular_org	Thermus aquaticus Y51MC23
1	Cellular_org	Thermus scotoductus
1	Cellular_org	Thermus scotoductus SA-01
1	Cellular_org	Thermus sp. 4C
1	Cellular_org	Thermus thermophilus
1	Cellular_org	Thermus thermophilus HB27
1	Cellular_org	Thermus thermophilus HB8
1	Cellular_org	Thioalkalivibrio sp. HL-EbGR7
1	Cellular_org	Thioalkalivibrio sp. K90mix
1	Cellular_org	Thiobacillus denitrificans
1	Cellular_org	Thiobacillus denitrificans ATCC 25259
1	Cellular_org	Thiomicrospira crunogena
1	Cellular_org	Thiomicrospira crunogena XCL-2

1	Cellular_org	Thiomonas intermedia K12
1	Cellular_org	Thiomonas sp. 3As
1	Cellular_org	Tolumonas auensis DSM 9187
1	Cellular_org	Tomato big bud phytoplasma
1	Cellular_org	Treponema denticola
1	Cellular_org	Treponema denticola ATCC 35405
1	Cellular_org	Treponema pallidum
1	Cellular_org	Treponema pallidum subsp. pallidum SS14
1	Cellular_org	Treponema pallidum subsp. pallidum str. Nichols
1	Cellular_org	Treponema phagedenis
1	Cellular_org	Treponema vincentii
1	Cellular_org	Treponema vincentii ATCC 35580
1	Cellular_org	Trichodesmium erythraeum
1	Cellular_org	Trichodesmium erythraeum IMS101
1	Cellular_org	Tropheryma whipplei
1	Cellular_org	Tropheryma whipplei TW08/27
1	Cellular_org	Tropheryma whipplei str. Twist
1	Cellular_org	Truepera radiovictrix DSM 17093
1	Cellular_org	Tsukamurella paurometabola
1	Cellular_org	Tsukamurella paurometabola DSM 20162
1	Cellular_org	Ureaplasma parvum
1	Cellular_org	Ureaplasma parvum serovar 1 str. ATCC 27813
1	Cellular_org	Ureaplasma parvum serovar 14 str. ATCC 33697
1	Cellular_org	Ureaplasma parvum serovar 3
1	Cellular_org	Ureaplasma parvum serovar 3 str. ATCC 27815
1	Cellular_org	Ureaplasma parvum serovar 3 str. ATCC 700970
1	Cellular_org	Ureaplasma parvum serovar 6
1	Cellular_org	Ureaplasma parvum serovar 6 str. ATCC 27818
1	Cellular_org	Ureaplasma urealyticum
1	Cellular_org	Ureaplasma urealyticum serovar 10 str. ATCC 33699
1	Cellular_org	Ureaplasma urealyticum serovar 11 str. ATCC 33695
1	Cellular_org	Ureaplasma urealyticum serovar 12 str. ATCC 33696
1	Cellular_org	Ureaplasma urealyticum serovar 13 str. ATCC 33698
1	Cellular_org	Ureaplasma urealyticum serovar 2 str. ATCC 27814
1	Cellular_org	Ureaplasma urealyticum serovar 4 str. ATCC 27816
1	Cellular_org	Ureaplasma urealyticum serovar 5 str. ATCC 27817
1	Cellular_org	Ureaplasma urealyticum serovar 7 str. ATCC 27819
1	Cellular_org	Ureaplasma urealyticum serovar 8 str. ATCC 27618
1	Cellular_org	Ureaplasma urealyticum serovar 9 str. ATCC 33175
1	Cellular_org	Variovorax paradoxus
1	Cellular_org	Variovorax paradoxus S110
1	Cellular_org	Veillonella atypica
1	Cellular_org	Veillonella atypica ACS-049-V-Sch6

1	Cellular_org	Veillonella atypica ACS-134-V-Col7a
1	Cellular_org	Veillonella dispar
1	Cellular_org	Veillonella dispar ATCC 17748
1	Cellular_org	Veillonella parvula
1	Cellular_org	Veillonella parvula ATCC 17745
1	Cellular_org	Veillonella parvula DSM 2008
1	Cellular_org	Veillonella sp. 3_1_44
1	Cellular_org	Veillonella sp. 6_1_27
1	Cellular_org	Verminephrobacter eiseniae
1	Cellular_org	Verminephrobacter eiseniae EF01-2
1	Cellular_org	Verrucomicrobiae bacterium DG1235
1	Cellular_org	Verrucomicrobium spinosum
1	Cellular_org	Verrucomicrobium spinosum DSM 4136
1	Cellular_org	Vibrio alginolyticus
1	Cellular_org	Vibrio alginolyticus 12G01
1	Cellular_org	Vibrio alginolyticus 40B
1	Cellular_org	Vibrio brasiliensis
1	Cellular_org	Vibrio caribbenthicus ATCC BAA-2122
1	Cellular_org	Vibrio cholerae
1	Cellular_org	Vibrio cholerae 12129(1)
1	Cellular_org	Vibrio cholerae 1587
1	Cellular_org	Vibrio cholerae 2740-80
1	Cellular_org	Vibrio cholerae 623-39
1	Cellular_org	Vibrio cholerae AM-19226
1	Cellular_org	Vibrio cholerae B33
1	Cellular_org	Vibrio cholerae BX 330286
1	Cellular_org	Vibrio cholerae CT 5369-93
1	Cellular_org	Vibrio cholerae INDRE 91/1
1	Cellular_org	Vibrio cholerae M66-2
1	Cellular_org	Vibrio cholerae MAK 757
1	Cellular_org	Vibrio cholerae MJ-1236
1	Cellular_org	Vibrio cholerae MO10
1	Cellular_org	Vibrio cholerae MZO-2
1	Cellular_org	Vibrio cholerae MZO-3
1	Cellular_org	Vibrio cholerae NCTC 8457
1	Cellular_org	Vibrio cholerae O1
1	Cellular_org	Vibrio cholerae O1 biovar El Tor
1	Cellular_org	Vibrio cholerae O1 biovar El Tor str. N16961
1	Cellular_org	Vibrio cholerae O395
1	Cellular_org	Vibrio cholerae RC27
1	Cellular_org	Vibrio cholerae RC385
1	Cellular_org	Vibrio cholerae RC9
1	Cellular_org	Vibrio cholerae TM 11079-80

1	Cellular_org	Vibrio cholerae TMA 21
1	Cellular_org	Vibrio cholerae V51
1	Cellular_org	Vibrio cholerae V52
1	Cellular_org	Vibrio cholerae bv. albensis
1	Cellular_org	Vibrio cholerae bv. albensis VL426
1	Cellular_org	Vibrio coralliilyticus
1	Cellular_org	Vibrio coralliilyticus ATCC BAA-450
1	Cellular_org	Vibrio fischeri ES114
1	Cellular_org	Vibrio fischeri MJ11
1	Cellular_org	Vibrio fluvialis
1	Cellular_org	Vibrio furnissii
1	Cellular_org	Vibrio furnissii CIP 102972
1	Cellular_org	Vibrio harveyi
1	Cellular_org	Vibrio harveyi 1DA3
1	Cellular_org	Vibrio harveyi ATCC BAA-1116
1	Cellular_org	Vibrio harveyi HY01
1	Cellular_org	Vibrio metschnikovii
1	Cellular_org	Vibrio metschnikovii CIP 69.14
1	Cellular_org	Vibrio mimicus
1	Cellular_org	Vibrio mimicus VM223
1	Cellular_org	Vibrio mimicus VM573
1	Cellular_org	Vibrio mimicus VM603
1	Cellular_org	Vibrio nigripulchritudo
1	Cellular_org	Vibrio orientalis
1	Cellular_org	Vibrio orientalis CIP 102891
1	Cellular_org	Vibrio parahaemolyticus
1	Cellular_org	Vibrio parahaemolyticus 16
1	Cellular_org	Vibrio parahaemolyticus AN-5034
1	Cellular_org	Vibrio parahaemolyticus AQ3810
1	Cellular_org	Vibrio parahaemolyticus AQ4037
1	Cellular_org	Vibrio parahaemolyticus K5030
1	Cellular_org	Vibrio parahaemolyticus Peru-466
1	Cellular_org	Vibrio parahaemolyticus RIMD 2210633
1	Cellular_org	Vibrio shilonii
1	Cellular_org	Vibrio shilonii AK1
1	Cellular_org	Vibrio sinaloensis
1	Cellular_org	Vibrio sp. 09022
1	Cellular_org	Vibrio sp. 0908
1	Cellular_org	Vibrio sp. 23023
1	Cellular_org	Vibrio sp. 41
1	Cellular_org	Vibrio sp. AND4
1	Cellular_org	Vibrio sp. Ex25
1	Cellular_org	Vibrio sp. MED222

1	Cellular_org	Vibrio sp. RC341
1	Cellular_org	Vibrio sp. RC586
1	Cellular_org	Vibrio sp. TC68
1	Cellular_org	Vibrio splendidus
1	Cellular_org	Vibrio splendidus 12B01
1	Cellular_org	Vibrio splendidus LGP32
1	Cellular_org	Vibrio tapetis
1	Cellular_org	Vibrio vulnificus
1	Cellular_org	Vibrio vulnificus CMCP6
1	Cellular_org	Vibrio vulnificus YJ016
1	Cellular_org	Vibrionales bacterium SWAT-3
1	Cellular_org	Victivallis vadensis ATCC BAA-548
1	Cellular_org	Waddlia chondrophila
1	Cellular_org	Waddlia chondrophila WSU 86-1044
1	Cellular_org	Weissella cibaria
1	Cellular_org	Weissella paramesenteroides
1	Cellular_org	Weissella paramesenteroides ATCC 33313
1	Cellular_org	Wigglesworthia glossinidia
1	Cellular_org	Wigglesworthia glossinidia endosymbiont of Glossina brevipalpis
1	Cellular_org	Wolbachia endosymbiont of Brugia malayi
1	Cellular_org	Wolbachia endosymbiont of Culex quinquefasciatus
1	Cellular_org	Wolbachia endosymbiont of Culex quinquefasciatus JHB
1	Cellular_org	Wolbachia endosymbiont of Culex quinquefasciatus Pel
1	Cellular_org	Wolbachia endosymbiont of Drosophila ananassae
1	Cellular_org	Wolbachia endosymbiont of Drosophila melanogaster
1	Cellular_org	Wolbachia endosymbiont of Drosophila simulans
1	Cellular_org	Wolbachia endosymbiont of Drosophila willistoni
1	Cellular_org	Wolbachia endosymbiont of Drosophila willistoni TSC#14030-0811.24
1	Cellular_org	Wolbachia endosymbiont of Muscidifurax uniraptor
1	Cellular_org	Wolbachia endosymbiont strain TRS of Brugia malayi
1	Cellular_org	Wolbachia sp. wRi
1	Cellular_org	Wolinella succinogenes
1	Cellular_org	Wolinella succinogenes DSM 1740
1	Cellular_org	Xanthobacter autotrophicus
1	Cellular_org	Xanthobacter autotrophicus Py2
1	Cellular_org	Xanthomonas albilineans
1	Cellular_org	Xanthomonas axonopodis
1	Cellular_org	Xanthomonas axonopodis pv. citri
1	Cellular_org	Xanthomonas axonopodis pv. citri str. 306
1	Cellular_org	Xanthomonas axonopodis pv. glycines
1	Cellular_org	Xanthomonas campestris

1	Cellular_org	Xanthomonas campestris pv. campestris
1	Cellular_org	Xanthomonas campestris pv. campestris str. 8004
1	Cellular_org	Xanthomonas campestris pv. campestris str. ATCC 33913
1	Cellular_org	Xanthomonas campestris pv. campestris str. B100
1	Cellular_org	Xanthomonas campestris pv. musacearum
1	Cellular_org	Xanthomonas campestris pv. musacearum NCPPB4381
1	Cellular_org	Xanthomonas campestris pv. vasculorum
1	Cellular_org	Xanthomonas campestris pv. vasculorum NCPPB702
1	Cellular_org	Xanthomonas campestris pv. vesicatoria str. 85-10
1	Cellular_org	Xanthomonas citri
1	Cellular_org	Xanthomonas euvesicatoria
1	Cellular_org	Xanthomonas fuscans
1	Cellular_org	Xanthomonas fuscans subsp. aurantifolii
1	Cellular_org	Xanthomonas fuscans subsp. aurantifolii str. ICPB 10535
1	Cellular_org	Xanthomonas fuscans subsp. aurantifolii str. ICPB 11122
1	Cellular_org	Xanthomonas oryzae
1	Cellular_org	Xanthomonas oryzae pv. oryzae
1	Cellular_org	Xanthomonas oryzae pv. oryzae KACC10331
1	Cellular_org	Xanthomonas oryzae pv. oryzae MAFF 311018
1	Cellular_org	Xanthomonas oryzae pv. oryzae PXO99A
1	Cellular_org	Xanthomonas oryzae pv. oryzicola
1	Cellular_org	Xanthomonas oryzae pv. oryzicola BLS256
1	Cellular_org	Xenorhabdus bovienii
1	Cellular_org	Xenorhabdus bovienii SS-2004
1	Cellular_org	Xenorhabdus nematophila
1	Cellular_org	Xenorhabdus nematophila ATCC 19061
1	Cellular_org	Xylanimonas cellulosilytica DSM 15894
1	Cellular_org	Xylella fastidiosa
1	Cellular_org	Xylella fastidiosa 9a5c
1	Cellular_org	Xylella fastidiosa Dixon
1	Cellular_org	Xylella fastidiosa M12
1	Cellular_org	Xylella fastidiosa M23
1	Cellular_org	Xylella fastidiosa Temecula1
1	Cellular_org	Xylella fastidiosa subsp. sandyi
1	Cellular_org	Yersinia aldovae
1	Cellular_org	Yersinia aldovae ATCC 35236
1	Cellular_org	Yersinia bercovieri
1	Cellular_org	Yersinia bercovieri ATCC 43970
1	Cellular_org	Yersinia enterocolitica
1	Cellular_org	Yersinia enterocolitica (type O:9)
1	Cellular_org	Yersinia enterocolitica subsp. enterocolitica
1	Cellular_org	Yersinia enterocolitica subsp. enterocolitica 8081
1	Cellular_org	Yersinia frederiksenii

1	Cellular_org	Yersinia frederiksenii ATCC 33641
1	Cellular_org	Yersinia intermedia
1	Cellular_org	Yersinia intermedia ATCC 29909
1	Cellular_org	Yersinia kristensenii
1	Cellular_org	Yersinia kristensenii ATCC 33638
1	Cellular_org	Yersinia mollaretii
1	Cellular_org	Yersinia mollaretii ATCC 43969
1	Cellular_org	Yersinia pestis
1	Cellular_org	Yersinia pestis Angola
1	Cellular_org	Yersinia pestis Antiqua
1	Cellular_org	Yersinia pestis CA88-4125
1	Cellular_org	Yersinia pestis CO92
1	Cellular_org	Yersinia pestis FV-1
1	Cellular_org	Yersinia pestis KIM 10
1	Cellular_org	Yersinia pestis KIM D27
1	Cellular_org	Yersinia pestis Nepal516
1	Cellular_org	Yersinia pestis Pestoides A
1	Cellular_org	Yersinia pestis Pestoides F
1	Cellular_org	Yersinia pestis Z176003
1	Cellular_org	Yersinia pestis biovar Antiqua str. B42003004
1	Cellular_org	Yersinia pestis biovar Antiqua str. E1979001
1	Cellular_org	Yersinia pestis biovar Antiqua str. UG05-0454
1	Cellular_org	Yersinia pestis biovar Mediaevalis str. K1973002
1	Cellular_org	Yersinia pestis biovar Microtus str. 91001
1	Cellular_org	Yersinia pestis biovar Orientalis str. F1991016
1	Cellular_org	Yersinia pestis biovar Orientalis str. IP275
1	Cellular_org	Yersinia pestis biovar Orientalis str. India 195
1	Cellular_org	Yersinia pestis biovar Orientalis str. MG05-1020
1	Cellular_org	Yersinia pestis biovar Orientalis str. PEXU2
1	Cellular_org	Yersinia pseudotuberculosis
1	Cellular_org	Yersinia pseudotuberculosis IP 31758
1	Cellular_org	Yersinia pseudotuberculosis IP 32953
1	Cellular_org	Yersinia pseudotuberculosis PB1/+
1	Cellular_org	Yersinia pseudotuberculosis YPIII
1	Cellular_org	Yersinia rohdei
1	Cellular_org	Yersinia rohdei ATCC 43380
1	Cellular_org	Yersinia ruckeri
1	Cellular_org	Yersinia ruckeri ATCC 29473
1	Cellular_org	Zunongwangia profunda SM-A87
1	Cellular_org	Zymomonas mobilis
1	Cellular_org	Zymomonas mobilis subsp. mobilis
1	Cellular_org	Zymomonas mobilis subsp. mobilis ATCC 10988
1	Cellular_org	Zymomonas mobilis subsp. mobilis CP4

1	Cellular_org	Zymomonas mobilis subsp. mobilis NCIMB 11163
1	Cellular_org	Zymomonas mobilis subsp. mobilis ZM4
1	Cellular_org	alpha proteobacterium BAL199
1	Cellular_org	alpha proteobacterium HIMB114
1	Cellular_org	alpha proteobacterium endosymbiont of Amoeba proteus
1	Cellular_org	bacterium Ellin514
1	Cellular_org	beta proteobacterium KB13
1	Cellular_org	candidate division TM7 genomosp. GTL1
1	Cellular_org	candidate division TM7 single-cell isolate TM7a
1	Cellular_org	candidate division TM7 single-cell isolate TM7b
1	Cellular_org	candidate division TM7 single-cell isolate TM7c
1	Cellular_org	cyanobacterium UCYN-A
1	Cellular_org	delta proteobacterium MLMS-1
1	Cellular_org	delta proteobacterium NaphS2
1	Cellular_org	endophytic bacterium LOB-07
1	Cellular_org	gamma proteobacterium HTCC2207
1	Cellular_org	gamma proteobacterium HTCC5015
1	Cellular_org	gamma proteobacterium HdN1
1	Cellular_org	gamma proteobacterium NOR5-3
1	Cellular_org	gamma proteobacterium NOR51-B
1	Cellular_org	marine actinobacterium PHSC20C1
1	Cellular_org	marine gamma proteobacterium HTCC2080
1	Cellular_org	marine gamma proteobacterium HTCC2143
1	Cellular_org	marine gamma proteobacterium HTCC2148
1	Cellular_org	uncultured Termite group 1 bacterium
1	Cellular_org	uncultured Termite group 1 bacterium phylotype Rs-D17
1	Cellular_org	uncultured bacterium
1	Cellular_org	uncultured eubacterium pIE1115
1	Cellular_org	unidentified eubacterium SCB49
1	Cellular_org	Acidianus ambivalens
1	Cellular_org	Acidianus hospitalis
1	Cellular_org	Acidilobus saccharovorans 345-15
1	Cellular_org	Aciduliprofundum boonei T469
1	Cellular_org	Aeropyrum pernix
1	Cellular_org	Aeropyrum pernix K1
1	Cellular_org	Archaeoglobus fulgidus
1	Cellular_org	Archaeoglobus fulgidus DSM 4304
1	Cellular_org	Archaeoglobus profundus
1	Cellular_org	Archaeoglobus profundus DSM 5631
1	Cellular_org	Caldivirga maquilingensis
1	Cellular_org	Caldivirga maquilingensis IC-167
1	Cellular_org	Candidatus Korarchaeum cryptofilum OPF8
1	Cellular_org	Cenarchaeum symbiosum

1	Cellular_org	Cenarchaeum symbiosum A
1	Cellular_org	Desulfurococcus
1	Cellular_org	Desulfurococcus kamchatkensis 1221n
1	Cellular_org	Desulfurococcus mucosus
1	Cellular_org	Ferroglobus placidus
1	Cellular_org	Ferroglobus placidus DSM 10642
1	Cellular_org	Ferroplasma acidarmanus fer1
1	Cellular_org	Haladaptatus paucihalophilus DX253
1	Cellular_org	Halalkalicoccus jeotgali B3
1	Cellular_org	Haloarcula
1	Cellular_org	Haloarcula marismortui
1	Cellular_org	Haloarcula marismortui ATCC 43049
1	Cellular_org	Haloarcula sp. AS7094
1	Cellular_org	Halobacterium salinarum
1	Cellular_org	Halobacterium salinarum R1
1	Cellular_org	Halobacterium sp. GN101
1	Cellular_org	Halobacterium sp. NRC-1
1	Cellular_org	Haloferax volcanii
1	Cellular_org	Haloferax volcanii DS2
1	Cellular_org	Halogeometricum borinquense
1	Cellular_org	Halogeometricum borinquense DSM 11551
1	Cellular_org	Halomicrobium mukohataei
1	Cellular_org	Halomicrobium mukohataei DSM 12286
1	Cellular_org	Haloquadratum walsbyi
1	Cellular_org	Haloquadratum walsbyi DSM 16790
1	Cellular_org	Halorhabdus utahensis
1	Cellular_org	Halorhabdus utahensis DSM 12940
1	Cellular_org	Halorubrum lacusprofundi
1	Cellular_org	Halorubrum lacusprofundi ATCC 49239
1	Cellular_org	Halorubrum saccharovorum
1	Cellular_org	Haloterrigena thermotolerans
1	Cellular_org	Haloterrigena turkmenica
1	Cellular_org	Haloterrigena turkmenica DSM 5511
1	Cellular_org	Hyperthermus butylicus
1	Cellular_org	Hyperthermus butylicus DSM 5456
1	Cellular_org	Ignicoccus hospitalis KIN4/I
1	Cellular_org	Ignisphaera aggregans
1	Cellular_org	Ignisphaera aggregans DSM 17230
1	Cellular_org	Metallosphaera sedula
1	Cellular_org	Metallosphaera sedula DSM 5348
1	Cellular_org	Methanobrevibacter ruminantium
1	Cellular_org	Methanobrevibacter ruminantium M1
1	Cellular_org	Methanobrevibacter smithii

1	Cellular_org	Methanobrevibacter smithii ATCC 35061
1	Cellular_org	Methanobrevibacter smithii DSM 2374
1	Cellular_org	Methanobrevibacter smithii DSM 2375
1	Cellular_org	Methanocaldococcus fervens AG86
1	Cellular_org	Methanocaldococcus infernus
1	Cellular_org	Methanocaldococcus infernus ME
1	Cellular_org	Methanocaldococcus jannaschii
1	Cellular_org	Methanocaldococcus jannaschii DSM 2661
1	Cellular_org	Methanocaldococcus sp. FS406-22
1	Cellular_org	Methanocaldococcus vulcanius M7
1	Cellular_org	Methanocella paludicola SANAE
1	Cellular_org	Methanococcoides burtonii
1	Cellular_org	Methanococcoides burtonii DSM 6242
1	Cellular_org	Methanococcus aeolicus
1	Cellular_org	Methanococcus aeolicus Nankai-3
1	Cellular_org	Methanococcus maripaludis
1	Cellular_org	Methanococcus maripaludis C5
1	Cellular_org	Methanococcus maripaludis C6
1	Cellular_org	Methanococcus maripaludis C7
1	Cellular_org	Methanococcus maripaludis S2
1	Cellular_org	Methanococcus vannielii
1	Cellular_org	Methanococcus vannielii SB
1	Cellular_org	Methanococcus voltae
1	Cellular_org	Methanococcus voltae A3
1	Cellular_org	Methanocorpusculum labreanum
1	Cellular_org	Methanocorpusculum labreanum Z
1	Cellular_org	Methanoculleus marisnigri JR1
1	Cellular_org	Methanohalobium evestigatum
1	Cellular_org	Methanohalobium evestigatum Z-7303
1	Cellular_org	Methanohalophilus mahii
1	Cellular_org	Methanohalophilus mahii DSM 5219
1	Cellular_org	Methanoplanus petrolearius DSM 11571
1	Cellular_org	Methanopyrus kandleri
1	Cellular_org	Methanopyrus kandleri AV19
1	Cellular_org	Methanosaeta thermophila PT
1	Cellular_org	Methanosarcina acetivorans
1	Cellular_org	Methanosarcina acetivorans C2A
1	Cellular_org	Methanosarcina barkeri
1	Cellular_org	Methanosarcina barkeri str. Fusaro
1	Cellular_org	Methanosarcina mazei
1	Cellular_org	Methanosarcina mazei Go1
1	Cellular_org	Methanosphaera stadtmanae
1	Cellular_org	Methanosphaera stadtmanae DSM 3091

1	Cellular_org	Methanosphaerula palustris
1	Cellular_org	Methanosphaerula palustris E1-9c
1	Cellular_org	Methanospirillum hungatei JF-1
1	Cellular_org	Methanothermobacter marburgensis
1	Cellular_org	Methanothermobacter marburgensis str. Marburg
1	Cellular_org	Methanothermobacter thermautotrophicus
1	Cellular_org	Methanothermobacter thermautotrophicus str. Delta H
1	Cellular_org	Methanothermococcus okinawensis
1	Cellular_org	Methanothermococcus okinawensis IH1
1	Cellular_org	Methanothermus fervidus
1	Cellular_org	Methanothermus fervidus DSM 2088
1	Cellular_org	Nanoarchaeum equitans Kin4-M
1	Cellular_org	Natrialba magadii
1	Cellular_org	Natrialba magadii ATCC 43099
1	Cellular_org	Natrinema sp. CX2021
1	Cellular_org	Natronobacterium sp. AS-7091
1	Cellular_org	Natronomonas pharaonis
1	Cellular_org	Natronomonas pharaonis DSM 2160
1	Cellular_org	Nitrosopumilus maritimus
1	Cellular_org	Nitrosopumilus maritimus SCM1
1	Cellular_org	Picrophilus torridus
1	Cellular_org	Picrophilus torridus DSM 9790
1	Cellular_org	Pyrobaculum aerophilum
1	Cellular_org	Pyrobaculum aerophilum str. IM2
1	Cellular_org	Pyrobaculum arsenaticum
1	Cellular_org	Pyrobaculum arsenaticum DSM 13514
1	Cellular_org	Pyrobaculum calidifontis
1	Cellular_org	Pyrobaculum calidifontis JCM 11548
1	Cellular_org	Pyrobaculum islandicum
1	Cellular_org	Pyrobaculum islandicum DSM 4184
1	Cellular_org	Pyrococcus abyssi
1	Cellular_org	Pyrococcus abyssi GE5
1	Cellular_org	Pyrococcus furiosus
1	Cellular_org	Pyrococcus furiosus DSM 3638
1	Cellular_org	Pyrococcus horikoshii
1	Cellular_org	Pyrococcus horikoshii OT3
1	Cellular_org	Pyrococcus sp. 12/1
1	Cellular_org	Pyrococcus sp. JT1
1	Cellular_org	Staphylothermus hellenicus
1	Cellular_org	Staphylothermus hellenicus DSM 12710
1	Cellular_org	Staphylothermus marinus
1	Cellular_org	Staphylothermus marinus F1
1	Cellular_org	Sulfolobus acidocaldarius

1	Cellular_org	Sulfolobus acidocaldarius DSM 639
1	Cellular_org	Sulfolobus islandicus
1	Cellular_org	Sulfolobus islandicus L.D.8.5
1	Cellular_org	Sulfolobus islandicus L.S.2.15
1	Cellular_org	Sulfolobus islandicus M.14.25
1	Cellular_org	Sulfolobus islandicus M.16.27
1	Cellular_org	Sulfolobus islandicus M.16.4
1	Cellular_org	Sulfolobus islandicus Y.G.57.14
1	Cellular_org	Sulfolobus islandicus Y.N.15.51
1	Cellular_org	Sulfolobus neozealandicus
1	Cellular_org	Sulfolobus solfataricus
1	Cellular_org	Sulfolobus solfataricus 98/2
1	Cellular_org	Sulfolobus solfataricus P2
1	Cellular_org	Sulfolobus sp. NOB8H2
1	Cellular_org	Sulfolobus tengchongensis
1	Cellular_org	Sulfolobus tokodaii
1	Cellular_org	Sulfolobus tokodaii str. 7
1	Cellular_org	Thermococcus barophilus MP
1	Cellular_org	Thermococcus gammatolerans EJ3
1	Cellular_org	Thermococcus kodakarensis KOD1
1	Cellular_org	Thermococcus nautilus
1	Cellular_org	Thermococcus onnurineus
1	Cellular_org	Thermococcus onnurineus NA1
1	Cellular_org	Thermococcus sibiricus MM 739
1	Cellular_org	Thermococcus sp. 26/2
1	Cellular_org	Thermococcus sp. AM4
1	Cellular_org	Thermococcus sp. AMT11
1	Cellular_org	Thermofilum pendens
1	Cellular_org	Thermofilum pendens Hrk 5
1	Cellular_org	Thermoplasma acidophilum
1	Cellular_org	Thermoplasma acidophilum DSM 1728
1	Cellular_org	Thermoplasma volcanium
1	Cellular_org	Thermoplasma volcanium GSS1
1	Cellular_org	Thermoproteus neutrophilus
1	Cellular_org	Thermoproteus neutrophilus V24Sta
1	Cellular_org	Thermosphaera aggregans DSM 11486
1	Cellular_org	Vulcanisaeta distributa
1	Cellular_org	Vulcanisaeta distributa DSM 14429
1	Cellular_org	uncultured methanogenic archaeon RC-I
2	Eukaryota	Arabidopsis thaliana
2	Eukaryota	Chlamydomonas reinhardtii
2	Eukaryota	Cucumis sativus
2	Eukaryota	Cyanidioschyzon merolae strain 10D

2	Eukaryota	Cyanophora paradoxa
2	Eukaryota	Dictyostelium discoideum AX4
2	Eukaryota	Entamoeba histolytica HM-1:IMSS
2	Eukaryota	Giardia lamblia ATCC 50803
2	Eukaryota	Leishmania braziliensis MHOM/BR/75/M2904
2	Eukaryota	Paramecium tetraurelia strain d4-2
2	Eukaryota	Physcomitrella patens subsp. patens
2	Eukaryota	Phytophthora infestans T30-4
2	Eukaryota	Picea sitchensis
2	Eukaryota	Plasmodium falciparum 3D7
2	Eukaryota	Tetrahymena thermophila
2	Eukaryota	Thalassiosira pseudonana CCMP1335
2	Eukaryota	Toxoplasma gondii ME49
2	Eukaryota	Trichomonas vaginalis G3
2	Eukaryota	Trypanosoma brucei TREU927
3	Opisthokonts	Ashbya gossypii ATCC 10895
3	Opisthokonts	Aspergillus fumigatus Af293
3	Opisthokonts	Aspergillus nidulans FGSC A4
3	Opisthokonts	Candida dubliniensis CD36
3	Opisthokonts	Candida glabrata CBS 138
3	Opisthokonts	Cryptococcus neoformans var. neoformans JEC21
3	Opisthokonts	Debaryomyces hansenii CBS767
3	Opisthokonts	Encephalitozoon cuniculi GB-M1
3	Opisthokonts	Gibberella zeae PH-1
3	Opisthokonts	Kluyveromyces lactis NRRL Y-1140
3	Opisthokonts	Magnaporthe oryzae 70-15
3	Opisthokonts	Monosiga brevicollis MX1
3	Opisthokonts	Neurospora crassa OR74A
3	Opisthokonts	Pichia pastoris GS115
3	Opisthokonts	Saccharomyces cerevisiae S288c
3	Opisthokonts	Scheffersomyces stipitis CBS 6054
3	Opisthokonts	Schizosaccharomyces pombe
3	Opisthokonts	Ustilago maydis 521
3	Opisthokonts	Yarrowia lipolytica CLIB122
4	Metazoa	Amphimedon queenslandica
4	Metazoa	Tetranychus urticae
5	Eumetazoa	Hydra magnipapillata
5	Eumetazoa	Nematostella vectensis
5	Eumetazoa	Trichoplax adhaerens
6	Bilateria	Bos taurus
6	Bilateria	Canis lupus familiaris
6	Bilateria	Danio rerio
6	Bilateria	Gallus gallus

6	Bilateria	Homo sapiens
6	Bilateria	Mus musculus
6	Bilateria	Pan troglodytes
6	Bilateria	Rattus norvegicus
6	Bilateria	Saccoglossus kowalevskii
6	Bilateria	Strongylocentrotus purpuratus
6	Bilateria	Tetraodon nigroviridis
6	Bilateria	Xenopus laevis
7	Protostomia	Acyrthosiphon pisum
7	Protostomia	Apis mellifera
7	Protostomia	Caenorhabditis elegans
7	Protostomia	Daphnia pulex
7	Protostomia	Drosophila melanogaster
8	Lophotrochozoa /Annelida	Capitella teleta
8	Lophotrochozoa /Annelida	Helobdella robusta
8	Lophotrochozoa /Annelida	Schistosoma japonicum
9	Mollusca/Gastro poda	Aplysia californica
9	Mollusca/Gastro poda	Lottia gigantea
10	Mollusca/Bivalvi a	Pinctada fucata
10	Mollusca/Bivalvi a	Crassostrea gigas
